# Supplementary material for: FAK activity in cancer‐associated fibroblasts is a prognostic marker and a druggable key metastatic player in pancreatic cancer
Source: EMBO Mol Med. 2020 Oct 7;12(11):e12010. doi: 10.15252/emmm.202012010 (PMC7645544; doi:10.15252/emmm.202012010)
Supplement: Supplementary file 1 — Appendix [file EMMM-12-e12010-s001.pdf]

Table of Content Appendix

Appendix Figure S1: pY397 FAK antibody (clone 31H5L17) validation.

Appendix Figure S2: Tumour cells and stroma identification using Definiens® software.

Appendix Figure S3: Representative flow cytometry plots of gating strategy.

Appendix Table S1: Exact p-values

Legend Appendix Figures and Table

Appendix Figure S1: pY397 FAK antibody (clone 31H5L17) validation.

(**A**) Representative images of phosphorylated FAK on tyrosine (Y) 397 (pY397 FAK) antibody immunohistochemistry staining in serial sections of human adjacent pancreas (Ct1) and human PDAC tissues. Left: classical immunohistochemistry, Middle: pY397 FAK antibody was pre-incubated for 12h with specific phosphopetide before used on slides. The total staining disappearance validates the antibody. Right: samples were treated with lambda-phosphatase before been exposed to pY397 FAK antibody.

(**B**) Left: Western blot performed on 50 µg of proteins from fibroblast (FAK wild type MEFs) lysates, pre-treated or not with lambda-phosphatase or Na3VO4 phosphatase inhibitor using pY397 FAK antibody. Right: pY397 FAK antibody was pre-incubated for 12h with specific phosphopetide before been used. (**C**) Western blot performed on 20 µg of proteins from FAK wild-type (WT) or FAK knock-out (KO: MEFs were isolated from murine homozygous *Fak* floxed embryos and treated with an adenovirus expressing the cre-recombinase (adeno-cre) (Lim et al, 2010)) lysates using pY397 FAK antibody. The disappearance of the 125 kDa band validates the antibody specificity. The presence of non-specific bands do not bother western blot analysis as they are around 50-60 kDa

Appendix Figure S2: Tumour cells and stroma identification using Definiens® software.

(**A**) Representative immunohistochemistry of pan-CK (pan-cytokeratin), pan-CK plus α-SMA, pY397 FAK and FAP-α in serial sections of 2 different PDAC tissues. For each patient, left: IHC staining; right: Definiens® ROI detection of tumour cells (dark blue), stroma (orange) and other (light blue/grey). Scale bar, 100 µm.

(**B**) Quantification of definiens-identified areas based on pan-CK (pan-cytokeratin), pan-CK plus α-SMA, pY397 FAK and FAP-α staining showing that independently on marker used, Definiens software is able to properly discriminate CAFs (α-SMA and/or FAP-α positive cells) from other cells (including isolated tumour cells, positive for pan-cytokeratin) based on cell morphology.

Appendix Figure S3: Representative flow cytometry plots of gating strategy.

(**A-B**) Representative flow cytometry plots of gating strategy for myeloid (**A**) and lymphoid cell (**B**) identification on mouse primary tumours. CD45: Total immune cells ; CD3 +: T lymphocyte CD3 cells;

CD4 +: T lymphocyte CD4 cells; CD8 +: Cytotoxic T lymphocyte CD8 cells; FOXP3 +: Forkhead box P3 T regulator lymphocytes, Tregs cell marker ; CD19: B lymphocytes ; NK1.1: Naturals Killers and Natural killers T (NK1.1+/CD3+) ; F480 +: Total macrophages ; F480 + / Nos2 +: Nitric oxide synthase, macrophage M1 marker ; F480+ / CD206 +: Macrophages M2 marker. Data processed using FlowJo software (Beckman Dickinson).

Appendix Table S1: Exact p-values

Appendix Figure S1

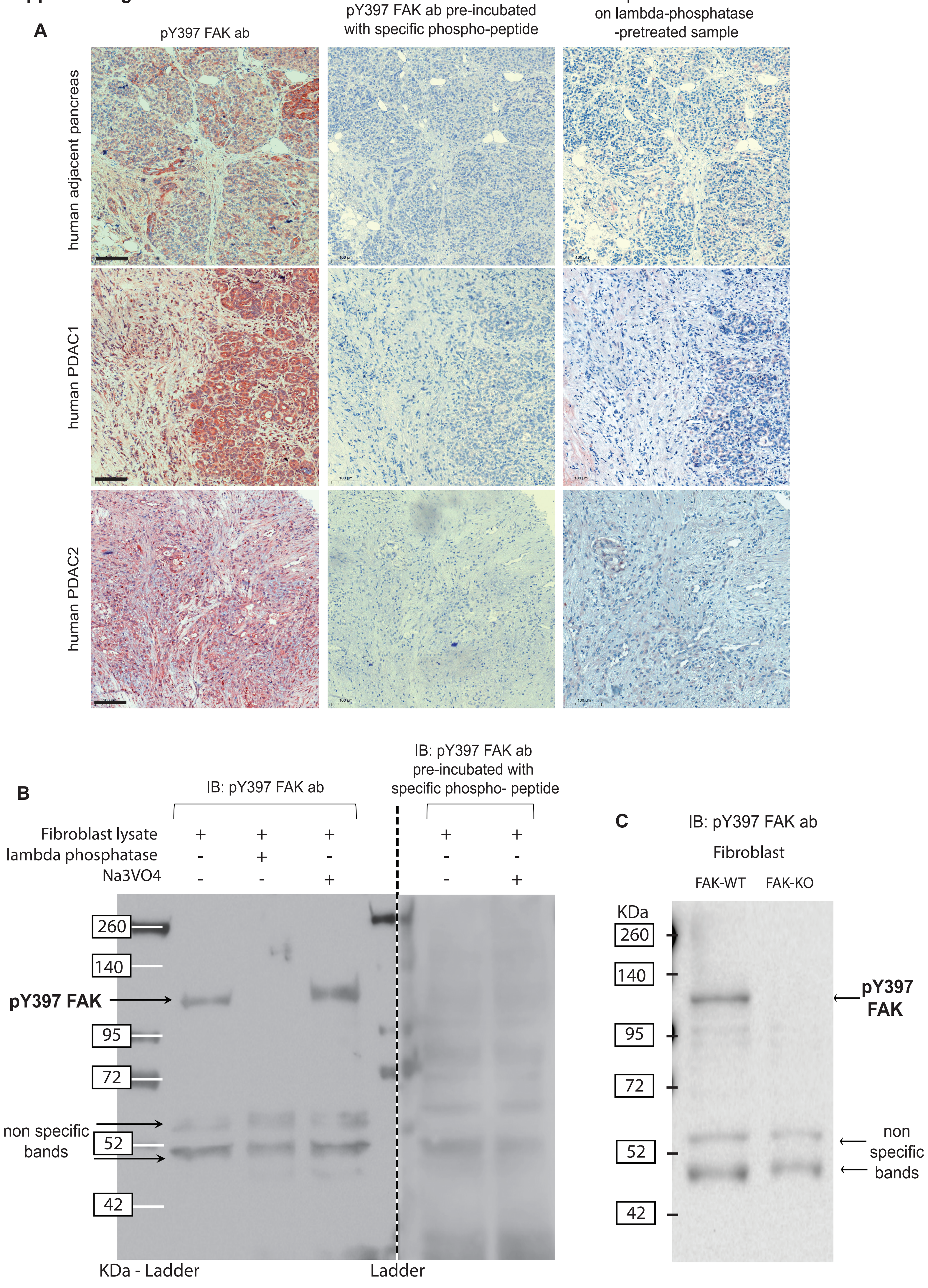

Appendix Figure S2

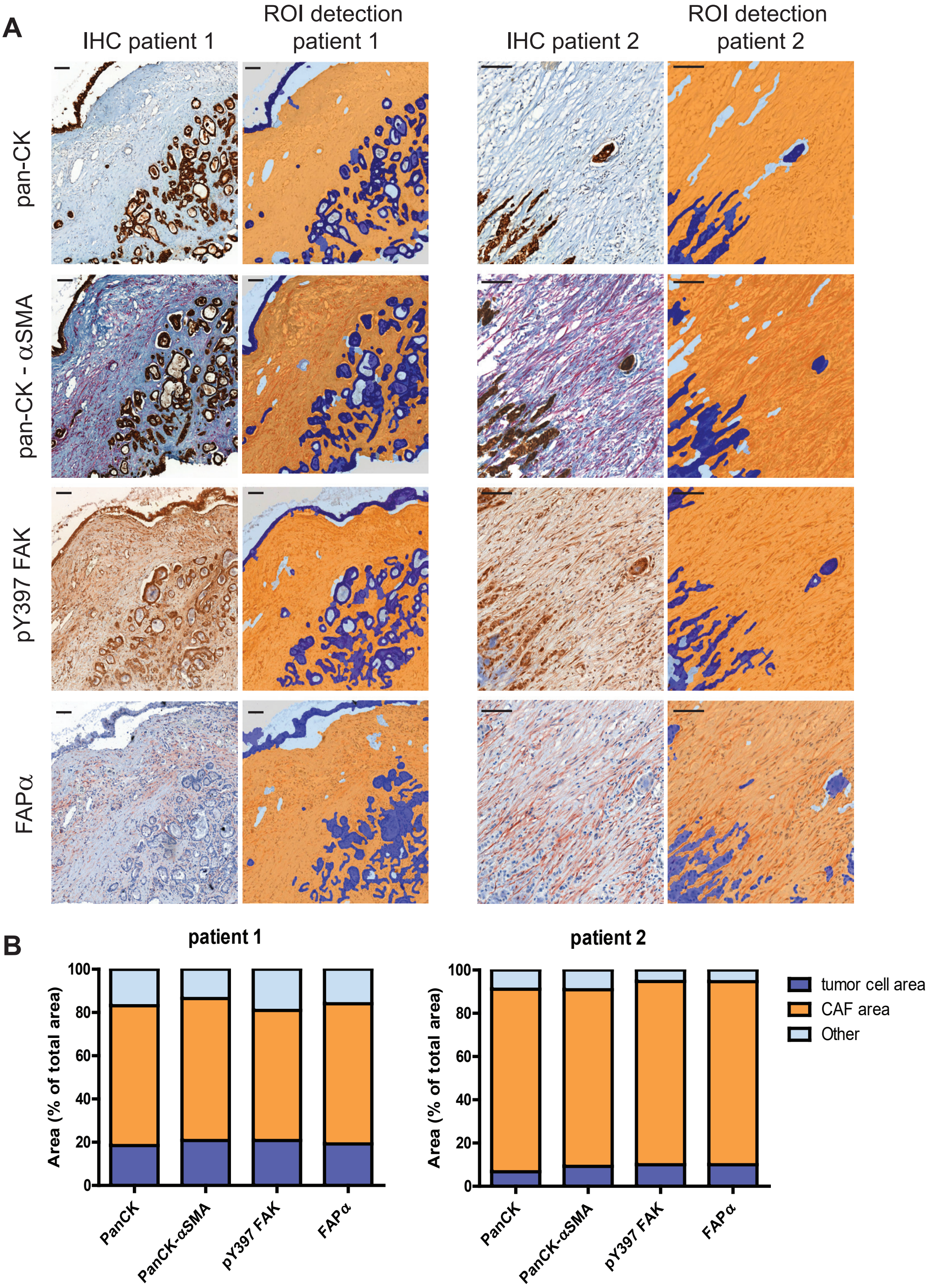

Appendix Figure S3

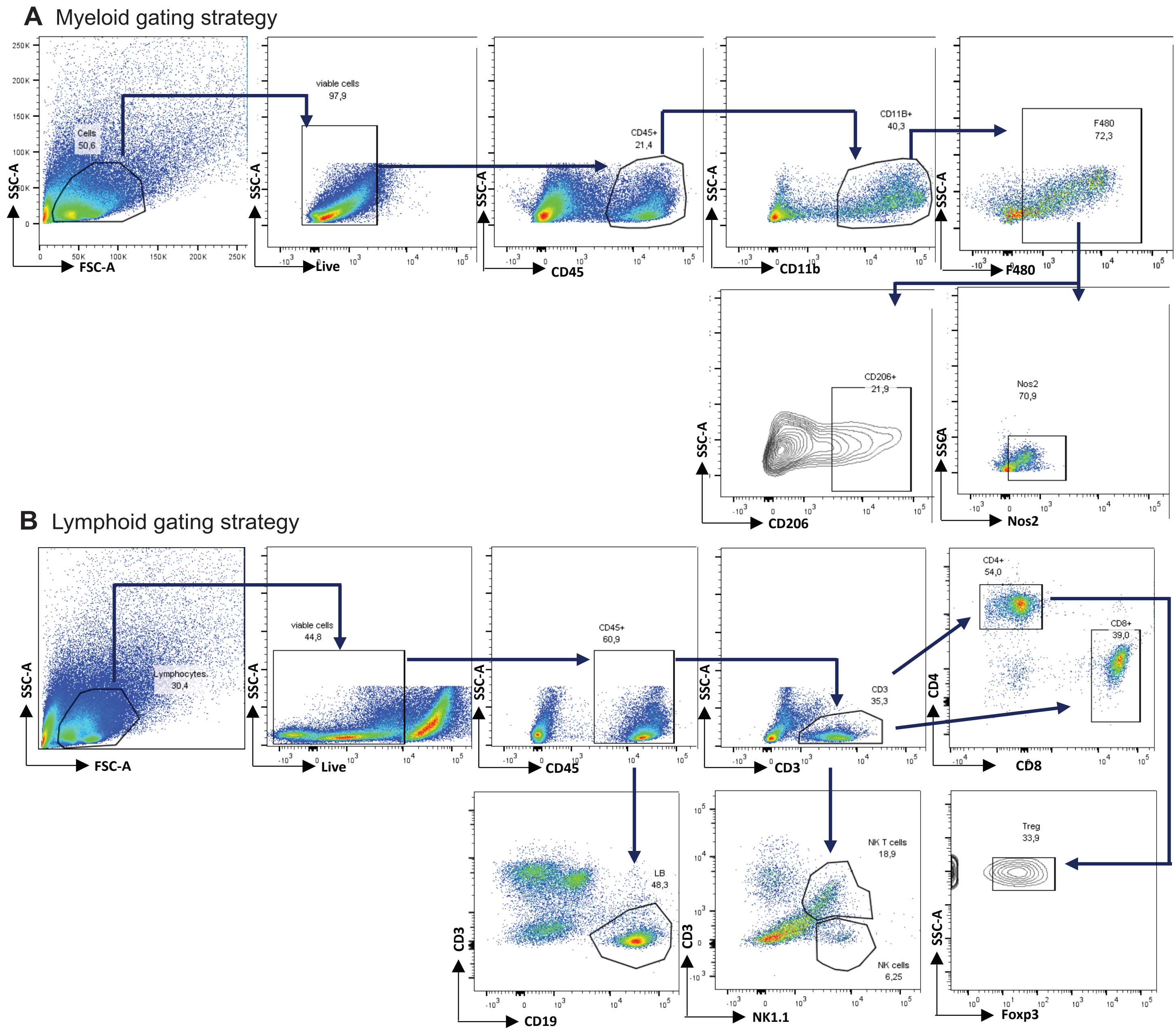

Appendix Table S1: Exact p-values

FIGURES	GRAPH	COMPARAISON	SIGNIFICANCE	ADJUSTED P VALUE	TEST
Fig 1C	pYFAK in fib.	Ct vs. PDAC	**	0,0016	Unpaired two-tailed t test
	pYFAK in fib. Cytoplasm	Ct vs. PDAC	**	0,0018	Unpaired two-tailed t test
	pYFAK in fib. Nucleus	Ct vs. PDAC	**	0,0039	Unpaired two-tailed t test
Fig 3C	Collagen I	R211+MEFsFAK-WT vs. R211+MEFsFAK-KD	*	0,0473	Unpaired two-tailed t test
	Collagen III	R211+MEFsFAK-WT vs. R211+MEFsFAK-KD	*	0,0452	Unpaired two-tailed t test
Fig 3G	Metastasis number	R211 vs. R211+MEFsFAK-WT	*	0,0152	Tukey's multiple comparisons test
		R211+MEFsFAK-WT vs. R211+MEFsFAK-KD	*	0,0365	Tukey's multiple comparisons test
	Metastasis area	R211 vs. R211+MEFsFAK-WT	***	0,0003	Tukey's multiple comparisons test
		R211+MEFsFAK-WT vs. R211+MEFsFAK-KD	**	0,0012	Tukey's multiple comparisons test
Fig 4 A	M1	TumCells+FibFAK-WT vs. TumCells+FibFAK-KD	*	0,0396	Unpaired two-tailed t test
	M2	TumCells+FibFAK-WT vs. TumCells+FibFAK-KD	**	0,0091	Unpaired two-tailed t test
Fig 4B	M2	TumCells+FibFAK-WT vs. TumCells+FibFAK-KD	*	0,0442	Unpaired two-tailed t test
Fig 4D	CD206low/CMH2high	FAK-WT CM vs FAK KD CM	*	0,0317	Unpaired two-tailed t test
	CD206high/CMH2low	FAK-WT CM vs FAK KD CM	*	0,0101	Unpaired two-tailed t test
	Dectin	FAK-WT CM vs FAK KD CM	*	0,0287	Unpaired two-tailed t test
Fig 4E	M2 48h	CM from CAFs vs CM from FAK-I CAFs	*	0,047	Paired two-tailed t test
Fig 4G	MCP1 elisa	NT vs FAK-I	*	0,016	Paired two-tailed t test
Fig 4H	CD206	fib pFAK low vs fib pFAK high	**	0,0084	Unpaired two-tailed t test
Fig 5C	fib. Velocity	FibFAK-WT vs. FibFAK-KD	****	<0,0001	Tukey's multiple comparisons test
		FibFAK-KD vs. FibFAK-KD+TumCell	**	0,0021	Tukey's multiple comparisons test
Fig 5D	fib directionality	FibFAK-WT vs. FibFAK-KD	****	<0,0001	Tukey's multiple comparisons test
		FibFAK-WT TumCell vs. FibFAK-KD TumCell	****	<0,0001	Tukey's multiple comparisons test
Fig 5E	fib. Distance	FibFAK-WT vs. FibFAK-KD TumCell	****	<0,0001	Tukey's multiple comparisons test
		FibFAK-WT+TumCell vs. FibFAK-KD+TumCell	****	<0,0001	Tukey's multiple comparisons test
		FibFAK-KD vs. FibFAK-KD+TumCell	****	<0,0001	Tukey's multiple comparisons test
Fig 5G	Tum Cell directionality	TumCell vs. TumCell+FibFAK-WT	*	0,0173	Tukey's multiple comparisons test
		TumCell vs. TumCell+FibFAK-KD	**	0,0073	Tukey's multiple comparisons test
		TumCell+FibFAK-WT vs. TumCell+FibFAK-KD	****	<0,0001	Tukey's multiple comparisons test
Fig 5H	Tum Cell distance	TumCell vs. TumCell+FibFAK-WT	**	0,0081	Tukey's multiple comparisons test
		TumCell+FibFAK-WT vs. TumCell+FibFAK-KD	*	0,02	Tukey's multiple comparisons test
Fig 5J	Fib invasion	FibFAK-WT+TumCell vs. FibFAK-KD+TumCell	***	0,0003	Unpaired two-tailed t test
Fig 5K	Tum Cell invasion	FibFAK-WT+TumCell vs. FibFAK-KD+TumCell	*	0,023	Unpaired two-tailed t test
		FibFAK-WT+TumCell vs. FibFAK-KD+TumCell D1	****	<0,0001	Tukey's multiple comparisons test
		FibFAK-WT+TumCell vs. FibFAK-KD+TumCell D2	***	0,0001	Tukey's multiple comparisons test
		FibFAK-WT+TumCell vs. FibFAK-KD+TumCell D3	****	<0,0001	Tukey's multiple comparisons test

Fig 6C	Mouse fib. + tum cells	FibFAK-WT+TumCell vs. FibFAK-KD+TumCell D4	****	<0,0001	Tukey's multiple comparisons test
		FibFAK-WT+TumCell vs. FibFAK-KD+TumCell D5	****	<0,0001	Tukey's multiple comparisons test
		FibFAK-WT+TumCell vs. FibFAK-KD+TumCell D6	***	0,0004	Tukey's multiple comparisons test
		FibFAK-WT+TumCell vs. FibFAK-KD+TumCell D7	***	0,0003	Tukey's multiple comparisons test
	Human primary CAFs	NT vs FAK-I D1	**	0,0075	Paired two-tailed t test
		NT vs FAK-I D2	**	0,0032	Paired two-tailed t test
		NT vs FAK-I D3	*	0,0115	Paired two-tailed t test
		NT vs FAK-I D4	**	0,0041	Paired two-tailed t test
		NT vs FAK-I D5	*	0,0278	Paired two-tailed t test
		NT vs FAK-I D7	**	0,0038	Paired two-tailed t test
Fig 6D	pY397 FAK	NT vs FAK-I	***	0,0006	Paired two-tailed t test
	Coll III	NT vs FAK-I	*	0,0473	Paired two-tailed t test
	Coll IV	NT vs FAK-I	**	0,0095	Paired two-tailed t test
	POSTN	NT vs FAK-I	***	0,0002	Paired two-tailed t test
	OPN	NT vs FAK-I	****	< 0,0001	Paired two-tailed t test
	LOXL2	NT vs FAK-I	*	0,0111	Paired two-tailed t test
Fig 6 L	cluster number	CAF14 NT vs. CAF14 FAK-I	****	<0,0001	Unpaired two-tailed t test
	cluster size	CAF14 NT vs. CAF14 FAK-I	****	<0,0001	Unpaired two-tailed t test
Fig EV2 A	pY397 FAK on GFP+ cells	FibFAK-WT+TumCell vs. FibFAK-KD+TumCell	*	0,0352	Unpaired one-tailed t test
Fig EV3C	CD206 number Tum area	FibFAK-WT+TumCell vs. FibFAK-KD+TumCell	*	0,0144	Unpaired two-tailed t test
	CD206 numb. Adj are	FibFAK-WT+TumCell vs. FibFAK-KD+TumCell	*	0,0442	Unpaired two-tailed t test
	CD206 num. Fibrotic area	FibFAK-WT+TumCell vs. FibFAK-KD+TumCell	*	0,0131	Unpaired two-tailed t test
Fig EV3F	M1 differentiation	M0 vs M1	****	<0,0001	Bonferroni's multiple comparisons test
	M2 differentiation	M0 vs M2	****	<0,0001	Bonferroni's multiple comparisons test
Fig EV3G	M2 migration	NT vs. MC	*	0,0381	Tukey's multiple comparisons test
		Pre-inc FAKi vs. MC	*	0,0301	Tukey's multiple comparisons test
		MC vs. MC+FAKi	*	0,0452	Tukey's multiple comparisons test
Fig EV5D	Mouse fibroblasts D1	FAK-WT fib. vs. FAK-WT fib. + TGFb	*	0,0128	Tukey's multiple comparisons test
		FAK-WT fib. vs. FAK-KD fib.	*	0,0195	Tukey's multiple comparisons test
		FAK-WT fib. vs. FAK-KD fib. + TGFb	*	0,0304	Tukey's multiple comparisons test
		FAK-WT fib. + TGFb vs. FAK-KD fib.	****	<0,0001	Tukey's multiple comparisons test
		FAK-WT fib. + TGFb vs. FAK-KD fib. + TGFb	****	<0,0001	Tukey's multiple comparisons test
	Mouse fibroblasts D2	FAK-WT fib. vs. FAK-WT fib. + TGFb	**	0,0038	Tukey's multiple comparisons test
		FAK-WT fib. vs. FAK-KD fib.	*	0,0432	Tukey's multiple comparisons test
		FAK-WT fib. + TGFb vs. FAK-KD fib.	****	<0,0001	Tukey's multiple comparisons test
		FAK-WT fib. + TGFb vs. FAK-KD fib. + TGFb	****	<0,0001	Tukey's multiple comparisons test
	Mouse fibroblasts D5	FAK-WT fib. vs. FAK-WT fib. + TGFb	**	0,0071	Tukey's multiple comparisons test
		FAK-WT fib. + TGFb vs. FAK-KD fib.	****	<0,0001	Tukey's multiple comparisons test
		FAK-WT fib. + TGFb vs. FAK-KD fib. + TGFb	****	<0,0001	Tukey's multiple comparisons test

	Mouse fibroblasts D6	FAK-WT fib. vs. FAK-WT fib. + TGFb	*	0,0107	Tukey's multiple comparisons test
		FAK-WT fib. + TGFb vs. FAK-KD fib.	****	<0,0001	Tukey's multiple comparisons test
		FAK-WT fib. + TGFb vs. FAK-KD fib. + TGFb	***	0,0004	Tukey's multiple comparisons test
	Mouse fibroblasts D7	FAK-WT fib. vs. FAK-WT fib. + TGFb	*	0,011	Tukey's multiple comparisons test
		FAK-WT fib. + TGFb vs. FAK-KD fib.	****	<0,0001	Tukey's multiple comparisons test
		FAK-WT fib. + TGFb vs. FAK-KD fib. + TGFb	**	0,0017	Tukey's multiple comparisons test
Fig EV5 L	cluster number	CAF1 NT vs. CAF1 FAK-I	*	0,0134	Tukey's multiple comparisons test
		CAF2 NT vs. CAF2 FAK-I	**	0,0041	Tukey's multiple comparisons test
	cluster size	CAF1 NT vs. CAF1 FAK-I	***	0,0005	Tukey's multiple comparisons test
		CAF2 NT vs. CAF2 FAK-I	**	0,0017	Tukey's multiple comparisons test
