# Supplementary material for: FAK activity in cancer‐associated fibroblasts is a prognostic marker and a druggable key metastatic player in pancreatic cancer
Source: EMBO Mol Med. 2020 Oct 7;12(11):e12010. doi: 10.15252/emmm.202012010 (PMC7645544; doi:10.15252/emmm.202012010)
Supplement: Supplementary file 2 — Expanded View Figures PDF [file EMMM-12-e12010-s002.pdf]

Expanded View Figures

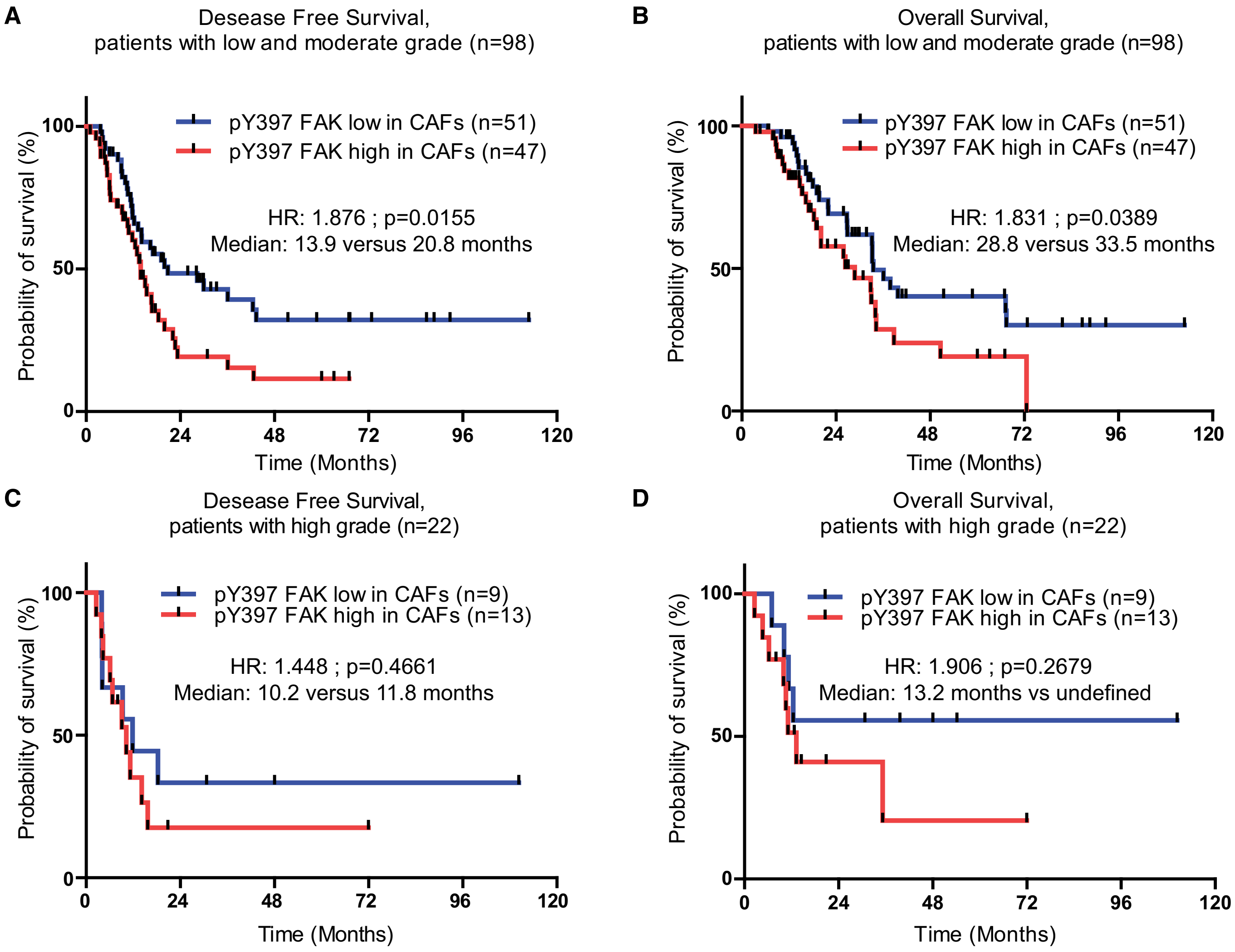

Figure EV1. Increased FAK activation is predictive of shorter DFS and OS for patients with low and moderate grade.

A, B Disease-free survival (A) and overall survival (B) according to pY397 FAK expression in CAFs of patients with low and moderate grade tumour.

C, D Disease-free survival (C) and overall survival (D) according to pY397 FAK expression in CAFs of patients with high grade tumour.

Data information: Survival curves were estimated with the Kaplan–Meier method and compared using log-rank test, non-parametric tests (chi-square test) to compare independent groups for categorical data; HR, hazard ratio.

© 2020 The Authors

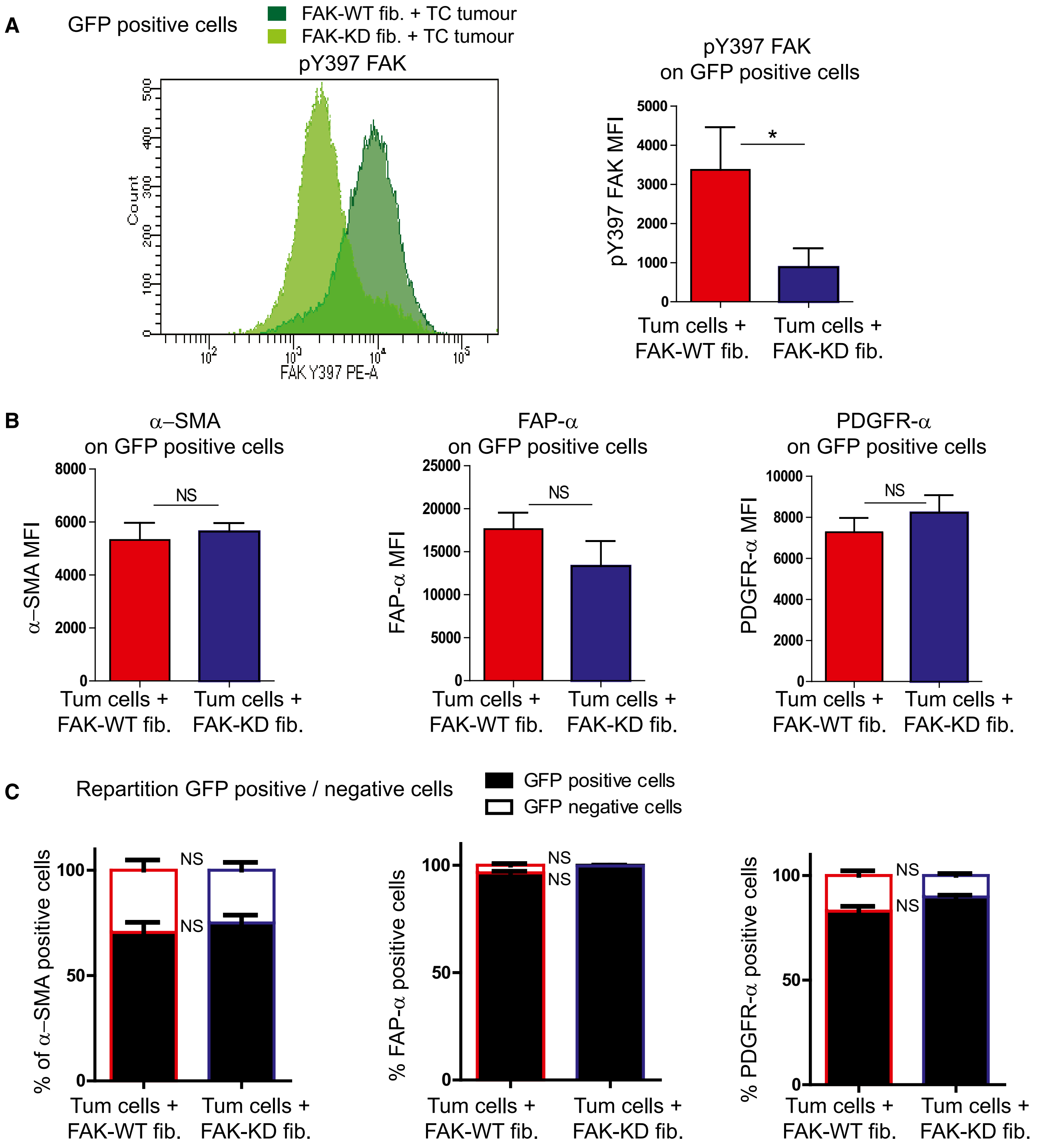

Figure EV2. Fibroblasts get activated when co-grafted with tumour cells.

Mice were syngeneically and orthotopically co-grafted with GFP-expressing FAK-WT or GFP-expressing FAK-KD mouse embryonic fibroblasts and pancreatic tumour cells (respectively, named GFP-FAK-WT fib. + TC or GFP-FAK-KD fib. + TC tumour). Fourty days after injection, mice were euthanized, tumour dissociated and CAF markers analysed using flow cytometry.

A, B pY397 FAK, α-SMA, FAP-α and PDGFR-α expressions were analysed on GFP-positive cells from GFP-FAK-WT fib. + TC tumours (dark green) or GFP-FAK-KD fib. + TC tumours (light green) MFI: mean of fluorescence intensity, values are means ± SEM using unpaired one-tailed Student's *t*-test from 6 mice per group. **P* < 0.05.

C Quantification of GFP-expressing cells on α-SMA-, FAP-α- or PDGFR-α-positive cells from GFP-FAK-WT fib. + TC or GFP-FAK-KD fib. + TC tumours. Values are means ± SEM using unpaired one-tailed Student's *t*-test from six mice per group. NS: not significantly different.

© 2020 The Authors

Figure EV3. Fibroblastic FAK inactivation reduces M2 macrophage polarization and migration.

A, B Twenty-one and 38 days after syngeneic and orthotopic co-grafting of mouse FAK-WT or FAK-KD fibroblasts plus tumour cells, mice were euthanized and tumour dissociated. Relative frequencies of tumour-infiltrating immune cells (CD3, CD4, CD8, LB, NK, NKT and Treg) using flow cytometry at 21 days (A) and 38 days (B). All stained cells were analysed with a Fortessa X20 flow cytometer (BD Biosciences), and data processed using FlowJo software (Beckman Dickinson). Values are means ± SEM from 5 to 10 mice per group, unpaired two-tailed Student's *t*-test.

C, D Quantification of CD206 (C)- and CD8 (D)-positive cell frequency (based on IHC staining) on whole pancreas, tumoural, adjacent and fibrotic areas with representative immunochemistry pictures of each staining on different areas. Values are means ± SEM from 9 to 10 mice per group. *$P < 0.05$ using unpaired two-tailed Student's *t*-test. Scale bar: 50 μm

E Mouse embryonic fibroblasts were incubated with conditioned medium (CM) from pancreatic cancer cells during 24, 48 or 72 h before being lysed (CM were harvested from tumour cells 72 h after tumour cells were seeded in F12 medium plus 0.5% FBS). PDGFR-α, FAP-α, α-SMA and GAPDH expressions were evaluated by Western blot.

F Validation of the BMDM-derived macrophage polarization into M1 or M2. Left: schematic of the experiment; right: percentage of M1- or M2-positive cells. Results were acquired by flow cytometry and processed using FlowJo software. ***$P < 0.001$, by Bonferroni's multiple comparison test; values are mean ± SEM from three experiments.

G Evaluation of the impact of FAK inhibitor that has been pre-incubated for 48 h at 37°C in DMEM/F12 + 0.5% FBS on macrophage migration. M, medium DMEM/F12 + 0.5% FBS; CM, conditioned medium. Quantification performed using Cell Observer videomicroscope (Zeiss) and analysed with ImageJ. *$P < 0.05$, by Tukey's multiple comparison test; values are mean ± SEM from three experiments.

© 2020 The Authors

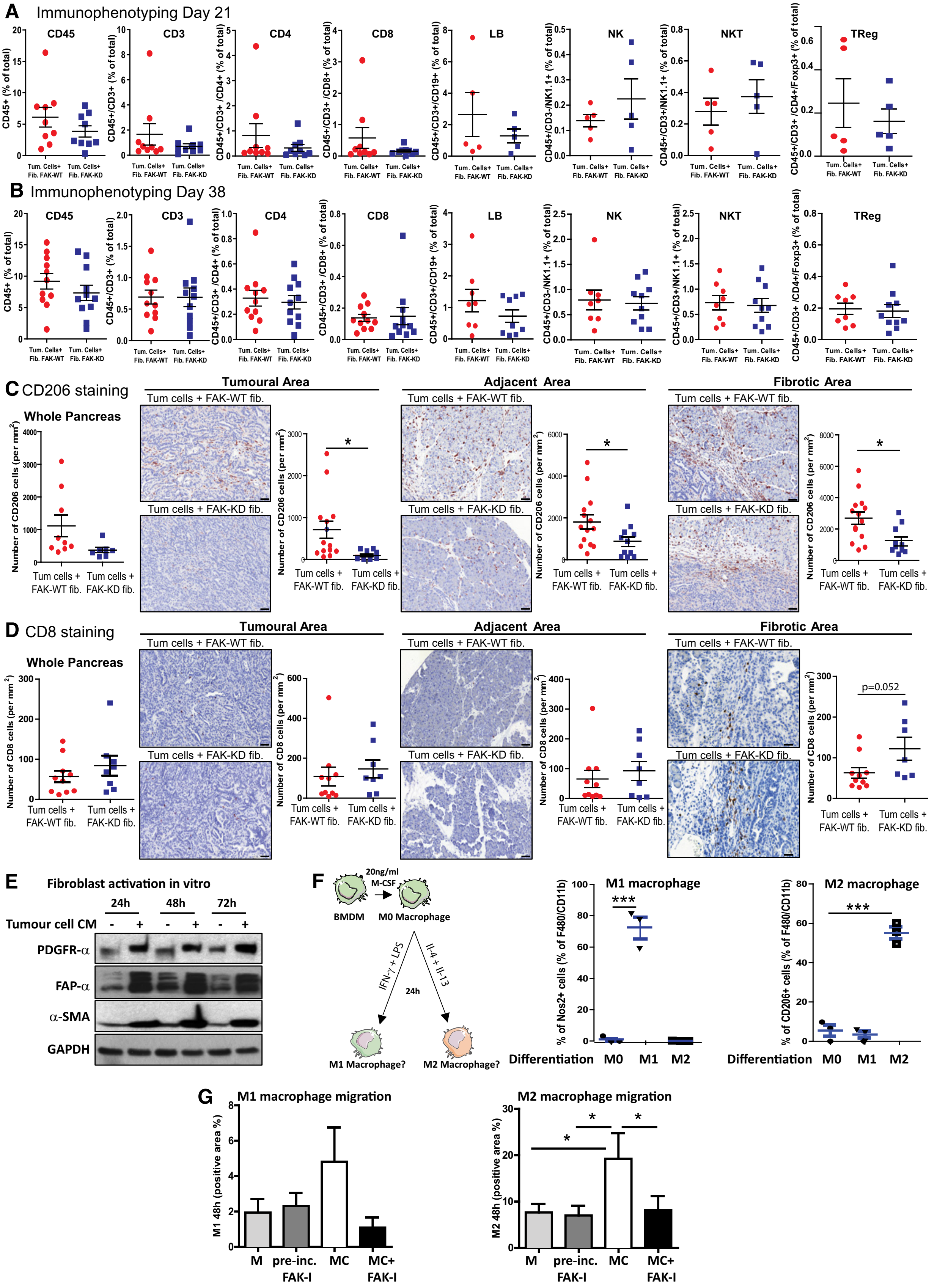
A Immunophenotyping Day 21
CD45
CD3
CD4
CD8
LB
NK
NKT
TReg
B Immunophenotyping Day 38
C CD206 staining
Whole Pancreas
Tumoural Area
Adjacent Area
Fibrotic Area
Tum cells + FAK-WT fib.
Tum cells + FAK-KD fib.
D CD8 staining
p=0.052
E Fibroblast activation in vitro
24h
48h
72h
Tumour cell CM
PDGFR-α
FAP-α
α-SMA
GAPDH
F
20ng/ml M-CSF
BMDM
M0 Macrophage
IFN-γ + LPS
Il-4 + Il-13
24h
M1 Macrophage?
M2 Macrophage?
M1 macrophage
M2 macrophage
Differentiation
M0
M1
M2
G
M1 macrophage migration
M2 macrophage migration
M
pre-inc. FAK-I
MC
MC+ FAK-I

Figure EV3.

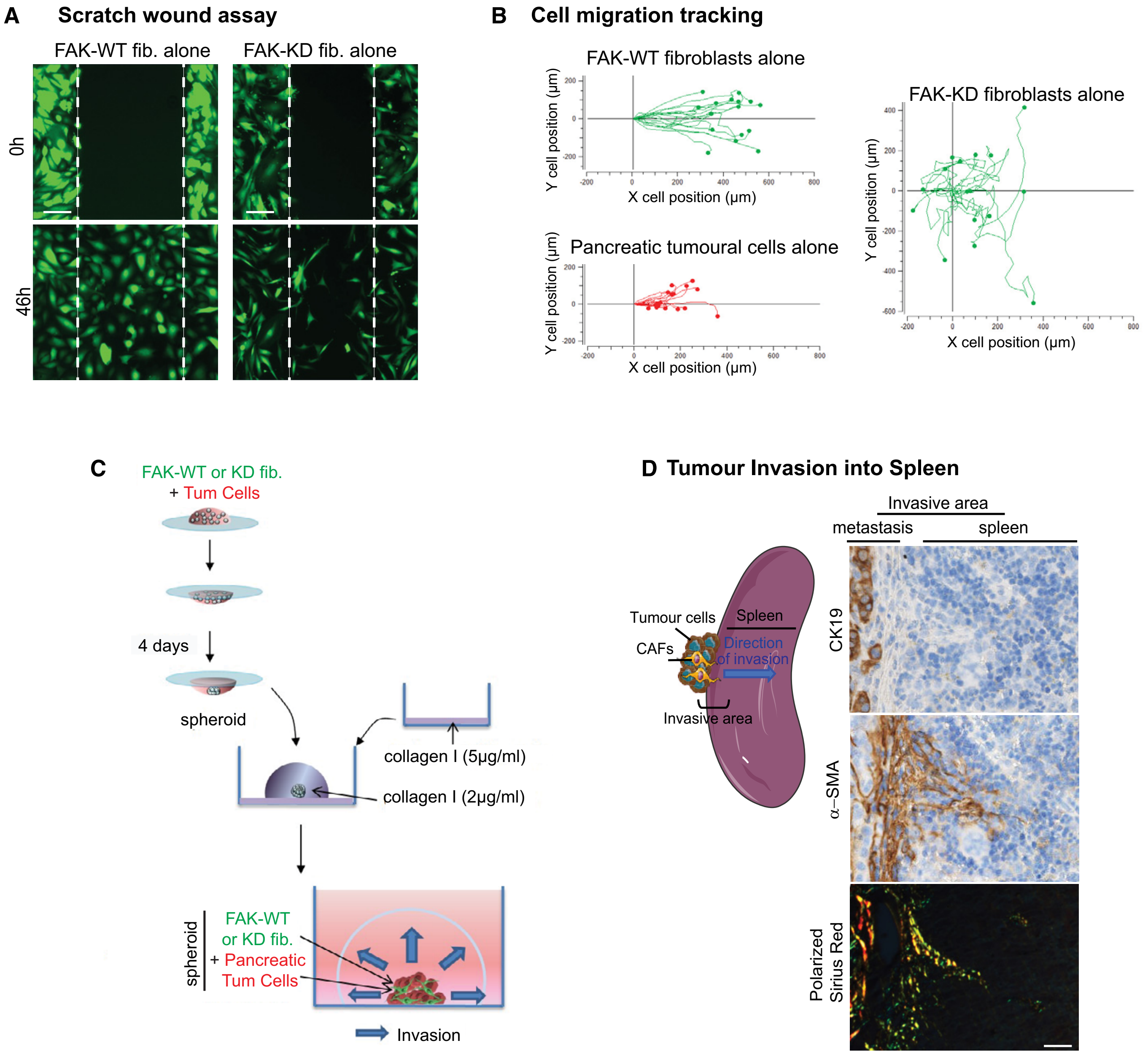

Figure EV4. Fibroblastic FAK activity controls tumour cell migration and invasion.

A Representative images of three independent scratch wound assays of either FAK-WT or FAK-KD green-labelled fibroblasts at time zero and 46 h. Scale bar, 150 µm.

B Cell migration tracking of either fibroblasts or tumour cells (n > 16 cells per condition) over time.

C Schematic of the method used to generate 3D spheroid of FAK-WT or FAK-KD fibroblasts plus pancreatic tumour cells in order to analyse cell invasion into collagen I matrix. A 20 µl drop containing FAK-WT or FAK-KD fibroblasts plus pancreatic tumour cells was hanged for 4 days at 37°C. New formed spheroids were transferred into well (eight-well bottomless µ-Slide Ibidi) pre-coated with 50 µg/ml collagen I matrix and embedded into 2 mg/ml collagen I matrix for 72 h. Z-stack of embedded spheroids was made using Zeiss LSM 780 confocal microscope.

D Representative pictures of pancreatic tumour invasion into the spleen. Left: schematic of the invasion; right: representative images of CK19 and α-SMA IHC staining and polarized Sirius Red performed on spleen sections of mice syngeneically and orthotopically co-grafted with pancreatic tumour cells and fibroblasts. Scale bar, 25 µm.

 © 2020 The Authors

Figure EV5. Fibroblastic FAK activity promotes drastic ECM modification.

A Representative images of CD19 and α-SMA immunohistochemistry and Sirius red staining in serial sections of two primary mouse tumours at 38 days after injection of mice grafted with FAK-WT or FAK-KD fibroblasts + tumours cells at the invasive front. Scale bar, 100 μm.

B Representative pictures of collagen gel contraction at day 0 and 7 induced by FAK-WT or FAK-KD fibroblasts, in presence or not of TGF-β.

C Representative pictures of fibroblast organization within collagen gel at day 12 observed using Nikon Eclipse TE200 microscope (40× magnifications) and Nikon Digital Camera DMX1200.

D Quantification of fibroblast-induced collagen I gel contraction based on photographed gels using the formula 100*(well diameter − gel diameter)/well diameter in presence or not of TGF-β.

E Representative pictures of immunofluorescence analyses of different protein expressed by human CAFs treated or not (NT) with FAK inhibitor (FAK-I at 1 μM). Scale bar, 50 μm.

F Percentage changes of protein expression for each individual patient-derived activated fibroblast tested. Percentage of pY397 FAK, total FAK, collagen I, collagen III, collagen IV, LOXL2, periostin and osteopontin expression after FAK inhibitor treatment of human primary CAFs. Each individual patient-derived activated fibroblast was assigned to a specific symbol (see Table EV2). Blue symbol colour was used when FAK-I promotes a decrease higher than 10%, red symbols for an increase higher than 10%, black symbols when FAK inhibition promotes variation lower than 10%.

G pY397 FAK, FAK, collagens I, III, IV, periostin, osteopontin and tubulin expressions were evaluated by Western blot on lysates from either hCAFs treated or not with FAK-I for 48 h, or from primary mouse tumours of mice grafted with FAK-WT or FAK-KD fibroblasts + tumours cells (day 38).

H–J Heatmap of the modification of ECM-affiliated protein (H), secreted factor (I) and ECM-regulator (J) expressions upon FAK inhibition (48 h) in hCAFs. The value of $\log_2$ fold change between FAK inhibitor-treated CAFs and non-treated CAFs for each gene was indicated by the coloured scale, with red indicating increased expression while blue implies decreased expression. For each CAFs and each gene, $\log_2$ fold change was calculated based on two technical replicates per condition (biological replicate $n = 3$, technical replicate $n = 2$).

K Representative pictures of β1 integrin activation of tumour cells, upon adhesion to ECM deposited from non-treated CAFs and after 20 min of $MnCl_2$ or EDTA treatment. Merge: activated β1 integrin (red), phalloidin (green), dapi (blue); scale bar: 10 μm.

L Left: Representative pictures of activated β1 integrin (top) or total β1 integrin (bottom) expressed by tumour cells, upon adhesion to either ECM deposited from non-treated CAFs or treated with FAK-I for 7 days, analysed by IF. Merge: integrin (red), phalloidin (green), dapi (blue); scale bar: 10 μm. Right: quantification of the number of activated integrin β1 cluster per cell and size of those clusters. Values are means ± SEM using one-way ANOVA with Tukey's method. *$P < 0.05$, **$P < 0.01$, ***$P < 0.001$ on at least seven images (of one to 34 cells per image) per group.

© 2020 The Authors

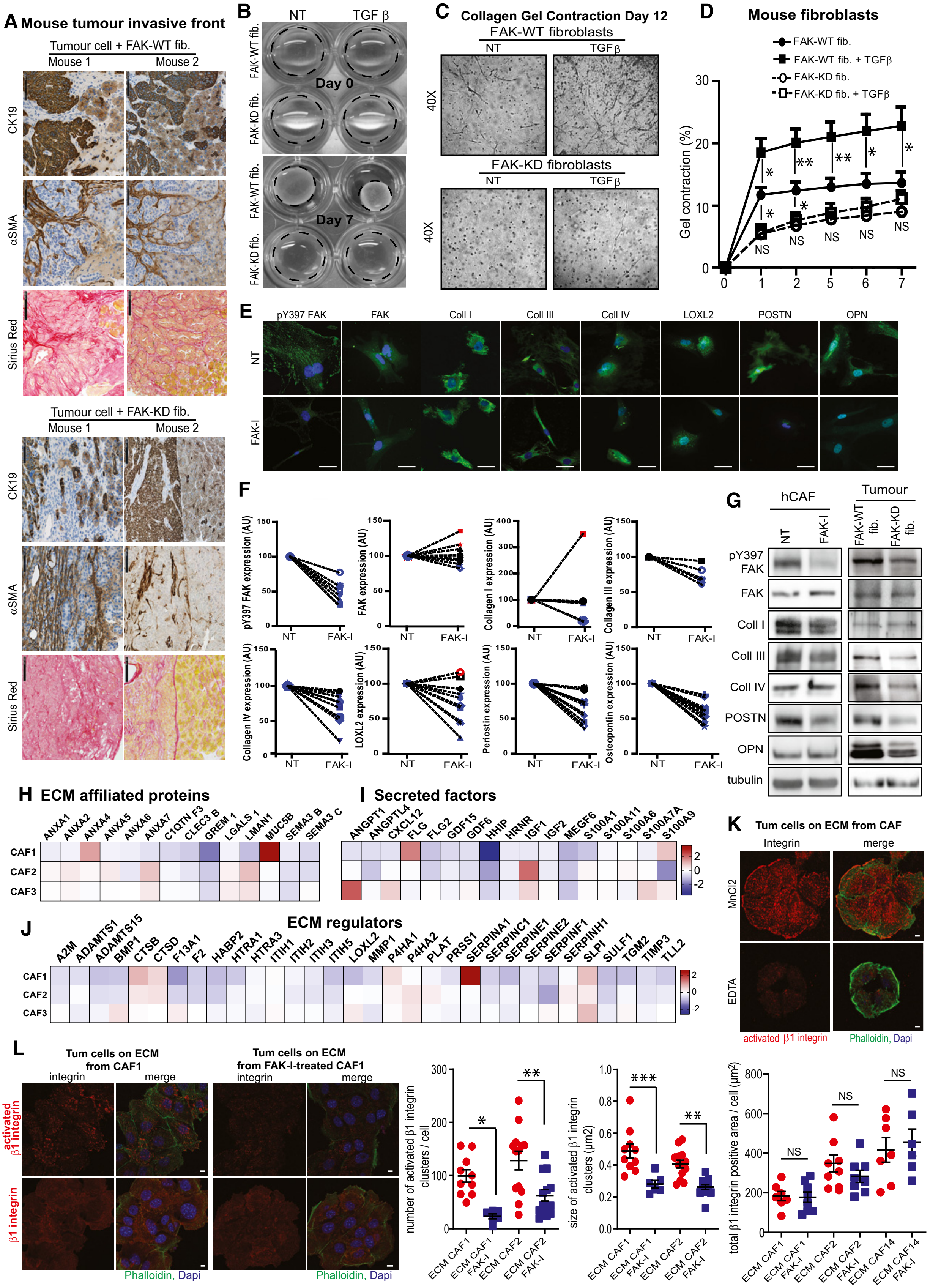

Figure EV5.

 © 2020 The Authors
