# Supplementary material for: FAK activity in cancer‐associated fibroblasts is a prognostic marker and a druggable key metastatic player in pancreatic cancer
Source: EMBO Mol Med. 2020 Oct 7;12(11):e12010. doi: 10.15252/emmm.202012010 (PMC7645544; doi:10.15252/emmm.202012010)
Supplement: Supplementary file 3 — Table EV1 [file EMMM-12-e12010-s003.pdf]

**Table EV1**. Clinical and pathological data for the 120 pancreatic ductal adenocarcinoma patients studied, and association with the fibroblastic pY397 FAK score

| Characteristic Total | Total (n=120) | Fib FAK pY397 low (n=60) | Fib FAK pY397 high (n=60) | p |
|---|---|---|---|---|
| Age (years), median (range) | 61 (37-79) | 60 (37-76) | 62 (43 -79) | NS |
| Male sex, no. (%) | 50 (42) | 25 (21) | 25 (21) | NS |
| Surgery, no. (%) | | | | |
| Duodenopancreatectomy | 101 (84) | 50 (42) | 51 (43) | NS |
| Distal pancreatectomy | 11 (9) | 6 (5) | 5 (4) | |
| Other | 8 (7) | 4 (3) | 4 (3) | |
| Resection margin, no. (%) | | | | |
| Negative, R0 | 88 (73) | 48 (40) | 40 (33) | NS |
| Positive, R1 | 32 (27) | 12 (10) | 20 (17) | |
| Tumour grade, no. (%) | | | | |
| Well differentiated | 60 (50) | 32 (27) | 28 (23) | NS |
| Moderately differentiated | 38 (32) | 19 (16) | 19 (16) | |
| Poorly differentiated | 20 (17) | 9 (8) | 11 (9) | |
| Unknown | 2 (2) | 0 (0) | 2 (2) | |
| Tumour size (mm) median (range) | 32 (10-150) | 33 (10-150) | 31 (10-105) | NS |
| Tumour stage, no. (%) | | | | |
| T1 | 3 (3) | 2 (2) | 1 (1) | NS |
| T2 | 14 (12) | 4 (3) | 10 (8) | |
| T3 | 103 (86) | 54 (45) | 49 (41) | |
| Lymph nodes, no. (%) | | | | |
| Positive, N+ | 94 (78) | 48 (40) | 46 (38) | NS |
| Ratio N+/N of ≤0.20 | 85 (71) | 42 (35) | 43 (36) | NS |
| Ratio N+/N of >0.20 | 35 (29) | 18 (15) | 17 (14) | |
| Tumour stage, AJCC 2009 (7th edn), no. (%) | | | | |
| IA | 2 (2) | 1 (1) | 1 (1) | NS |
| IB | 7 (6) | 3 (3) | 4 (3) | |
| IIA | 17 (14) | 8 (7) | 9 (8) | |
| IIB | 94 (78) | 48 (40) | 46 (38) | |
| Vascular invasion, no. (%) | | | | |
| No | 11 (9) | 7 (6) | 4 (3) | NS |
| Yes | 92 (77) | 43 (36) | 49 (41) | |
| Unknown | 17 (14) | 10 (8) | 7 (6) | |
| Perineural invasion, no. (%) | | | | |
| No | 12 (10) | 5 (4) | 7 (6) | NS |
| Yes | 88 (73) | 44 37) | 44 37) | |
| Unknown 19 | 20 (17) | 11 (9) | 9 (8) | |
| Adjuvant treatment, no. (%) | | | | |
| No | 21 (18) | 9 (8) | 12 (10) | NS |
| Gemcitabine | 40 (33) | 17 (14) | 23 (19) | |
| RT or RCT with 5FU | 19 (16) | 14 (12) | 5 (4) | |
| RCT with gemcitabine | 37 (31) | 18 (15) | 19 (16) | |
| Other | 2 (2) | 1 (1) | 1 (1) | |
| Unknown | 1 (1) | 1 (1) | 0 (0) | |

NS, not significant, AJCC, American Joint Committee on Cancer; RT, radiotherapy; RCT, radiochemotherapy; 5-FU, 5-fluorouracil.
