# Supplementary material for: FAK activity in cancer‐associated fibroblasts is a prognostic marker and a druggable key metastatic player in pancreatic cancer
Source: EMBO Mol Med. 2020 Oct 7;12(11):e12010. doi: 10.15252/emmm.202012010 (PMC7645544; doi:10.15252/emmm.202012010)
Supplement: Supplementary file 4 — Table EV2 [file EMMM-12-e12010-s004.pdf]

**Table EV2 :** Clinical and pathological data pancreatic ductal adenocarcinoma patients whose surgery-resected tumors were processed to derive primary cultures of CAFs

| | Male Sex | AGE | Differenciation | Tumor Size cm | Ratio N+/N >0.2 | Liver metastasis | TNM | Adjuvent treatment |
|---|---|---|---|---|---|---|---|---|
| ⬣ CAF1 | 1 | 68 | Moderately differentiated | 2.5 | 0 | 0 | pT3 N1 | Gemcitabine |
| ⬓ CAF2 | 1 | 44 | Moderately differentiated | .4 | 0 | 0 | pT1a N0 | FOLFIRINOX |
| ● CAF3 | 0 | 70 | Moderately differentiated | Unknown | 1 | 0 | pT3 N1 | No |
| ◇ CAF4 | 0 | 74 | Moderately differentiated | 6 | 0 | 0 | pT3 N0 | No |
| ◆ CAF5 | 1 | 56 | Unknown | Unknown | 0 | 0 | Unknown | No |
| ■ CAF6 | 0 | 78 | Moderately differentiated | 2 | 0 | 0 | pT1 N0 | FOLFIRINOX |
| ○ CAF7 | 1 | 68 | Moderately differentiated | 3.2 | 1 | 1 | pT2 N1b | No |
| + CAF8 | 1 | 54 | Well differentiated | 5 | 1 | 0 | pT3 N1 | No |
| ▲ CAF9 | 0 | 77 | Moderately differentiated | Unknown | 1 | 0 | pT2 N1 | No |
| □ CAF10 | 1 | 67 | Moderately differentiated | 2.8 | 0 | 0 | pT3 N0 | No |
| × CAF11 | 0 | 67 | Moderately differentiated | 6 | 1 | 0 | pT3 N1a | FOLFIRINOX |
| ★ CAF12 | 0 | 66 | Moderately differentiated | 3 | 0 | 0 | pT3 N1 | No |
| ⊗ CAF13 | 0 | 68 | Moderately differentiated | 3.2 | 0 | 0 | pT2 N0 R1 | No |
| ⊕ CAF14 | 0 | 47 | Moderately differentiated | 2.4 | 1 | 0 | pT2 N2 | No |
| ▼ CAF15 | 0 | 58 | Moderately differentiated | 3.5 | 0 | 0 | pT3 N0 | FOLFIRINOX |
