# Supplementary material for: FAK activity in cancer‐associated fibroblasts is a prognostic marker and a druggable key metastatic player in pancreatic cancer
Source: EMBO Mol Med. 2020 Oct 7;12(11):e12010. doi: 10.15252/emmm.202012010 (PMC7645544; doi:10.15252/emmm.202012010)
Supplement: Supplementary file 5 — Table EV3 [file EMMM-12-e12010-s005.pdf]

Table EV3. Antibodies and reagents (IHC: immunohistochemistry, IF: immunofluorescence, WB: Western-blot, FC: flow cytometry)

Antibody	Usage	Clone - Number	Company	dilution
anti-α-Smooth Muscle Actin (anti-α-SMA)	IHC	#ab7817	Abcam	1/100
anti-cytokeratin 19 (CK19)	IHC	#ab133496	Abcam	1/100
anti-pan-cytokeratin	IHC	MSK098	Zytomed	1/200
anti-pY397 FAK	IHC, WB	clone 31H5L17	ThermoFisher Scientific	1/500
anti-FAPα	IHC	ab207178	Abcam	1/500
ImmPRESS Mouse secondary antibody	IHC	#MP-7402	Vector	
ImmPRESS Rabbit secondary antibody	IHC	#MP7401	Vector	
anti-HIF1α	IHC	#ab2185	Abcam	1/1000
anti-CA IX	IHC	#PA1-16592	Invitrogen	1/2000
anti-CD31	IHC	#sc1506	Santa Cruz	1/400
anti-CD206	IHC	#ab64693	Abcam	1/1000
anti-CD8	IHC	#98941	Cell Signaling	1/100
anti-Periostin	IF, WB	#SC67233	Santa Cruz	1/100& 1/500
anti-osteopontin	IF, WB	#SC73631	Santa Cruz	1/100& 1/500
anti-Lysyl Oxydase Like 2 (LOXL2)	IF	#SC66950	Santa Cruz	1/100
anti-Lysyl Oxydase Like 2 (LOXL2)	WB	#ab96233	Abcam	1/1000
anti-pY397 FAK	IF, WB	#ab81298	abcam	1/200& 1/1000
anti-collagen I	IF, WB	#PA1-26204	ThermoFisher Scientific	1/100& 1/1000
anti-collagen III	IF, WB	#ab7778	abcam	1/100& 1/2000
anti-collagen IV	IF, WB	#ab6586	abcam	1/100& 1/2000
anti-FAK	IF, WB	#05-537 clone 4.47	EMD Millipore	1/100 & 1/1000
Anti-activated β1 integrin clone 9EG7	IF	#553715	BD Biosciences	1/50
Anti- β1 integrin -PE	IF	#102207	BioLegend	1/100
Goat anti-Mouse Alexa Fluor 488	IF	#A-11001	Invitrogen	1/1000
Goat anti-Rabbit Alexa Fluor 647	IF & FC	#A-21244	Invitrogen	1/1000 & 1/400
Goat anti-Rat Alexa Fluor 555	IF	#A-21434	Invitrogen	1/1000?
anti-pY397 FAK	FC	#ab81298	Abcam	1/50
anti-FAPα	FC	#ab28244	Abcam	1/50
Goat anti-rabbit Alexa Fluor 546	FC	#A-11010	Invitrogen	1/400
Goat anti-mouse Alexa Fluor 647	FC	#A28181	ThermoFisher Scientific	1/400
anti-PDGFRα-PE	FC	#562776	BD Biosciences	1/50
anti-αSMA-Alexa Fluor 647	FC	#ab196919	Abcam	1/50
anti-Foxp3-V450	FC	#561293	BD Biociences	1/100
anti-MHC-I-BV711	FC	#563414	BD Biociences	1/100
anti-GR1-PECy7	FC	# 552985	BD Biociences	1/100
anti-LY6G-BV421	FC	#562737	BD Biociences	1/100
anti-LY6C-APC-Cy7	FC	#560596	BD Biociences	1/100
anti-CD11b-APC-Cy7	FC	#557657	BD Biociences	1/200
anti-CD19-PECy7	FC	#115520	BioLegend	1/100

anti-F480-APC	FC	#17-4801-82	Invitrogen	1/100
anti-CD3-V450	FC	#561389	BD Biociences	1/100
anti-CD3-PECy5	FC	#553065	BD Biociences	1/100
anti-CD11b-PE-CF594	FC	#562287	BD Biociences	1/200
Live-BV605	FC	# 565694	BD Biociences	1/200
anti-CD45-V450	FC	#560501	BD Biociences	1/100
anti-CD4-BUV737	FC	#564933	BD Biociences	1/100
anti-CD8-APC	FC	#553035	BD Biociences	1/100
anti-NK1.1-FITC	FC	#108706	BioLegend	1/100
anti-Nos2-PECy7	FC	#25-5920-82	Invitrogen	1/200
anti-CD206-AF488	FC	#141710	BioLegend	1/100
anti-CD163-PE	FC	# 155307	BioLegend	1/200
anti-Dectin-PerCP-eFluor 710	FC	#46-5859-82	eBiosciences	1/200
anti-CD80-APC	FC	#17-0801-82	eBiosciences	1/200
anti-CD86-PE	FC	#159203	BioLegend	1/200
anti-MHC-II-APC-eFluor 780	FC	#47-5321-82	eBiosciences	1/800

**Reagent**	**Usage**	**Clone - Number**	**Company**	**dilution**
DAB (3,3’-diaminobenzidine)	IHC	#SK4105	Vector	
AEC (aminoethyl carbazole)	IHC	#K3464	DAKO	
Glycergel	IHC	#C0563	DAKO	
Pico-Sirius Red Solution	IHC	#ab150681	abcam	
DAPI	IF	#D9545	Sigma-Aldrich	1/1000
Fluorescent mounting Medium	IF	#S3023	DAKO	
Tumor Dissociation Kit	FC	130-096-730	Miltenyi Biotec	
Fix/Perm solution	FC	#00-5123-43	ebiosciences	
Permeabilization buffer	FC	#00-8333-56	ebiosciences	
