# Supplementary material for: FAK activity in cancer‐associated fibroblasts is a prognostic marker and a druggable key metastatic player in pancreatic cancer
Source: EMBO Mol Med. 2020 Oct 7;12(11):e12010. doi: 10.15252/emmm.202012010 (PMC7645544; doi:10.15252/emmm.202012010)
Supplement: Supplementary file 6 — Movie EV1 [file EMMM-12-e12010-s006.zip › Movie EV1.docx]

**Movie EV1:** Representative scratch wound assay movie from three independent experiments of red-labelled pancreatic tumour cells (mCherry-expressing cells) and FAK-Wild-type green-labelled fibroblasts (GFP- expressing cells) from time zero to 72 hours. Cell migration was recorded at 3 min interval for 72h using Cell Observer videomicroscope motorized with AxioObserver Z1 (ZEISS); Objective 10x EC Plan-Neofluar; Source Colibri 2LED (ZEISS), software Zen Blue 2012.
