# Supplementary figures and images for: FAK activity in cancer‐associated fibroblasts is a prognostic marker and a druggable key metastatic player in pancreatic cancer
Source: EMBO Mol Med. 2020 Oct 7;12(11):e12010. doi: 10.15252/emmm.202012010 (PMC7645544; doi:10.15252/emmm.202012010)

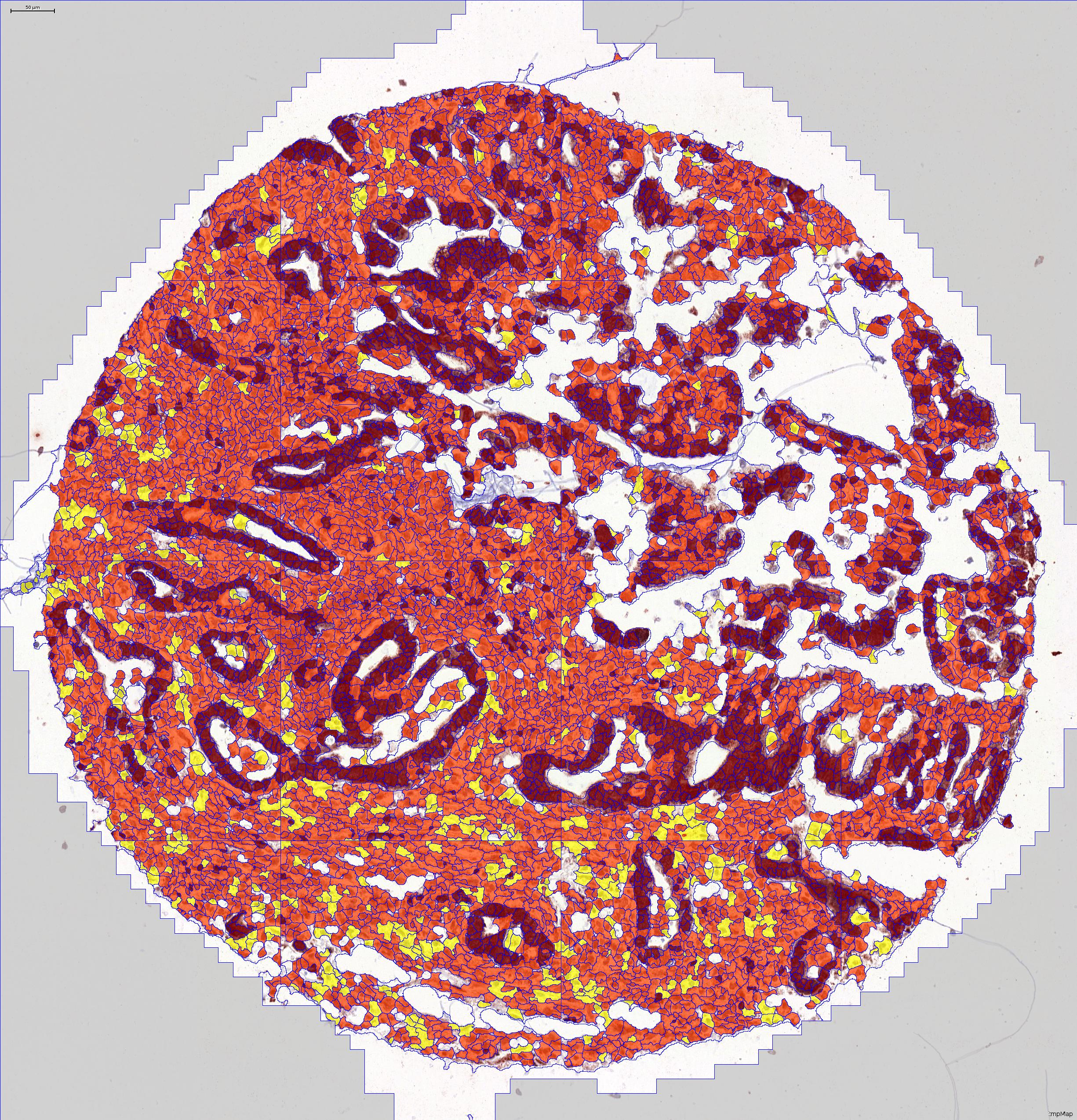

Supplement: Supplementary file 10 — Source Data for Figure 2 [file EMMM-12-e12010-s009.zip › Fig2A/Fig2_TMA109yFAK.20x.subsets.Core_78_4CellLevel_OverlayOutlines_.v2.jpg]

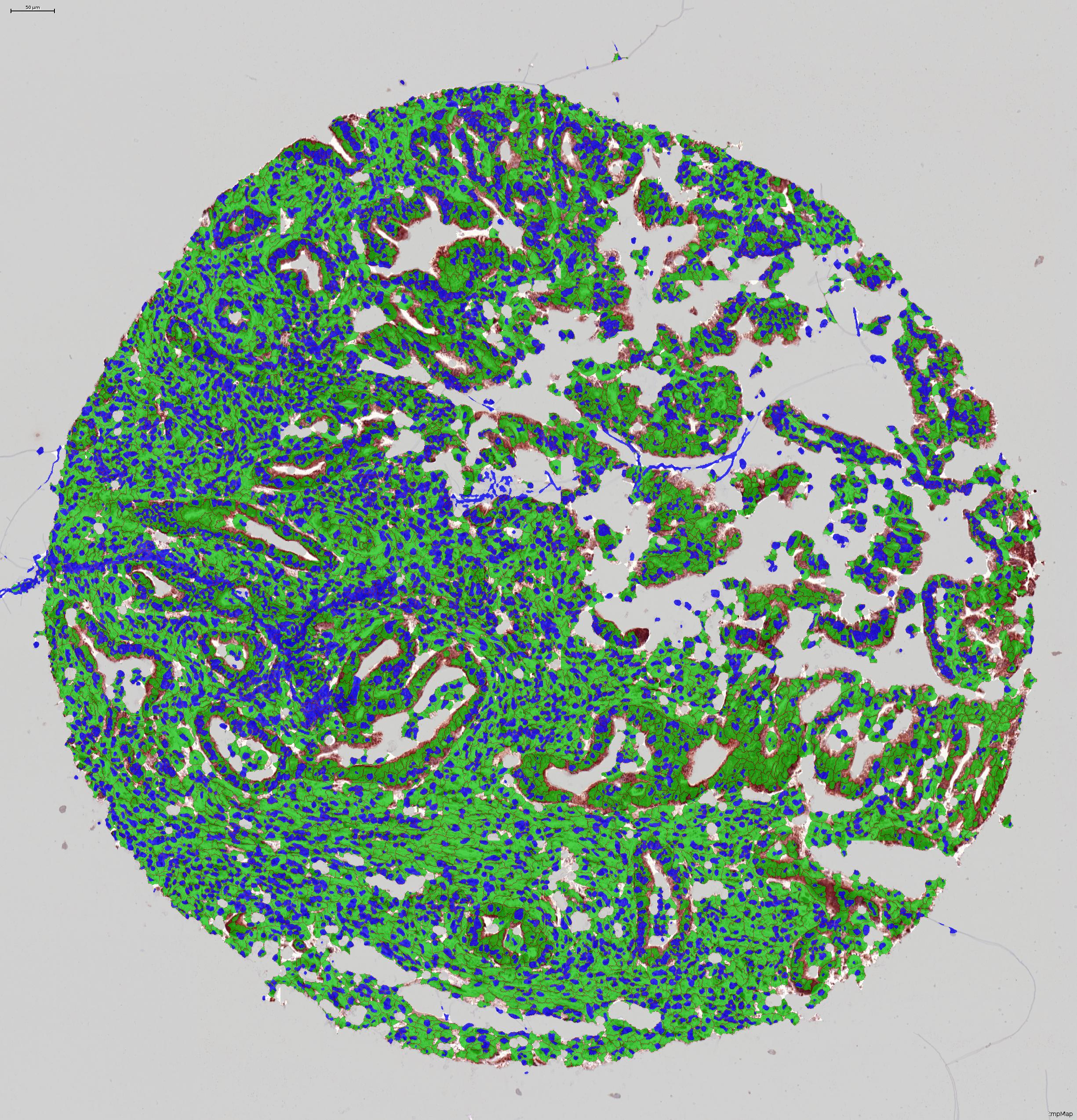

Supplement: Supplementary file 10 — Source Data for Figure 2 [file EMMM-12-e12010-s009.zip › Fig2A/Fig2_TMA109yFAK.20x.subsets.Core_78_4NucleusLevel_OverlayOutlines_.v2.jpg]

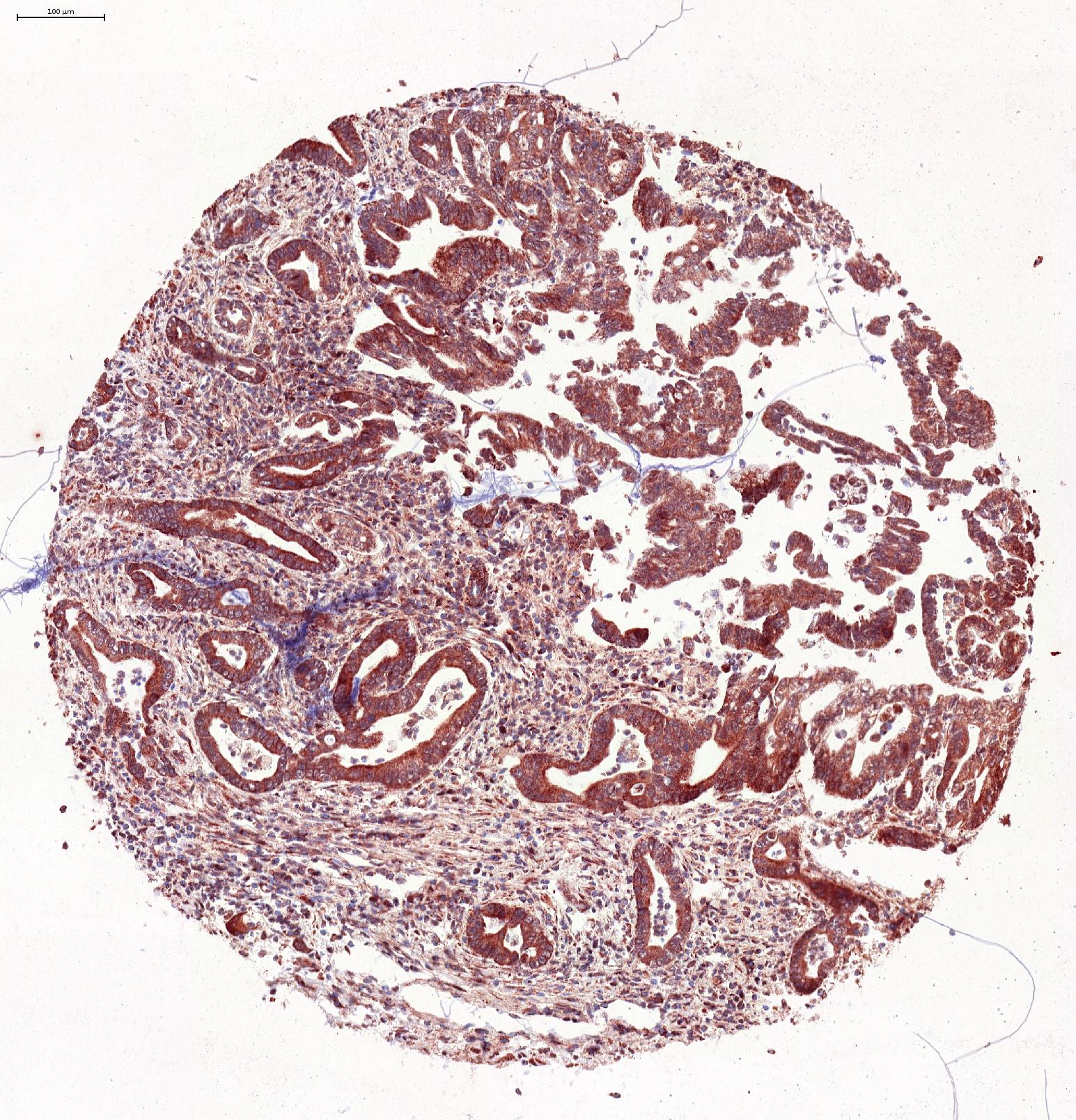

Supplement: Supplementary file 10 — Source Data for Figure 2 [file EMMM-12-e12010-s009.zip › Fig2A/Fig2_TMA109yFAK.20x.subsets.Core_78_ROI_Original_.v2.jpg]

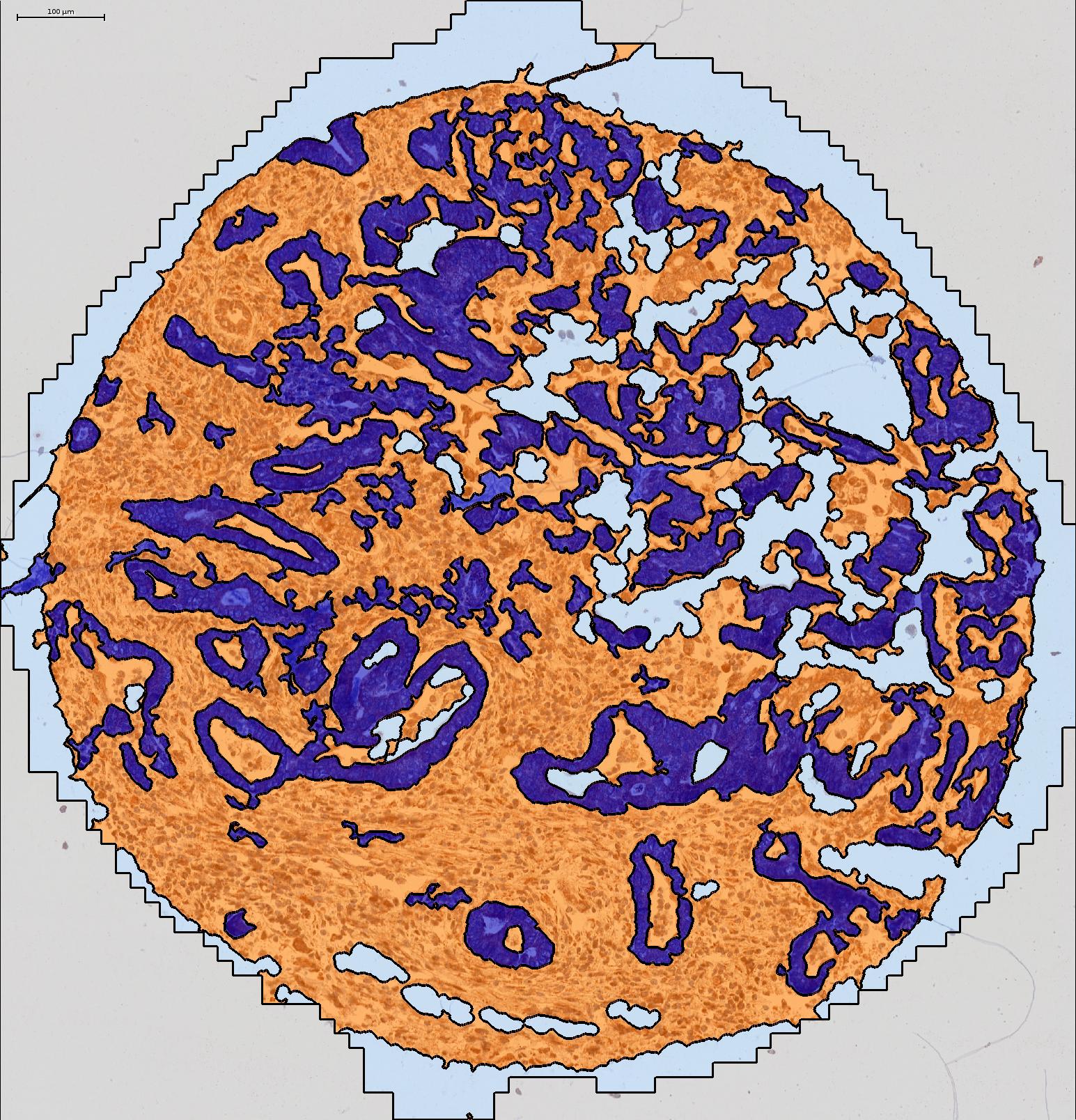

Supplement: Supplementary file 10 — Source Data for Figure 2 [file EMMM-12-e12010-s009.zip › Fig2A/Fig2_TMA109yFAK.20x.subsets.Core_78_ROI_OverlayOutlines_.v2.jpg]

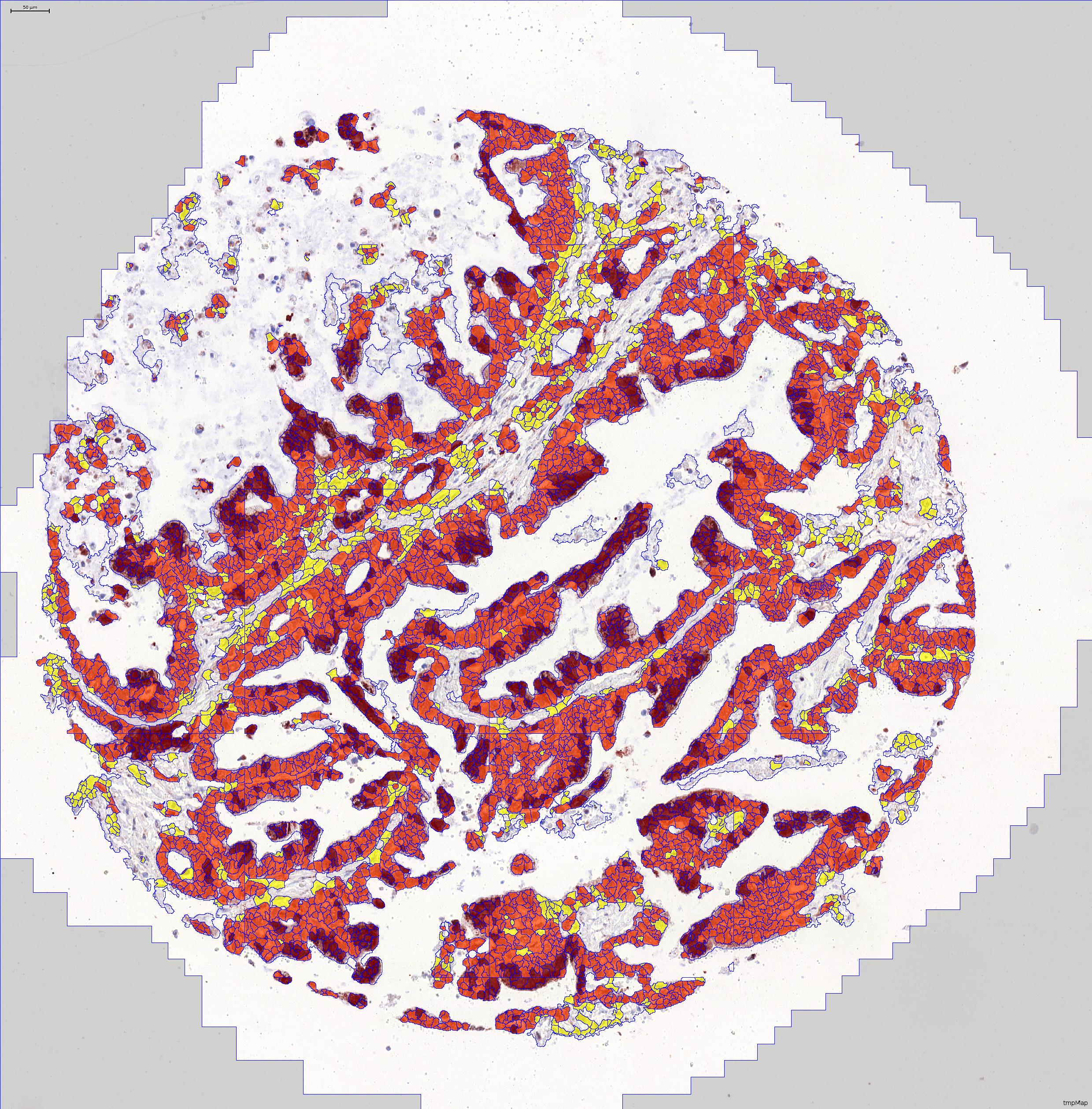

Supplement: Supplementary file 10 — Source Data for Figure 2 [file EMMM-12-e12010-s009.zip › Fig2A/Fig2_TMA94yFAK.20x.subsets.Core_10_4CellLevel_OverlayOutlines_.v2.jpg]

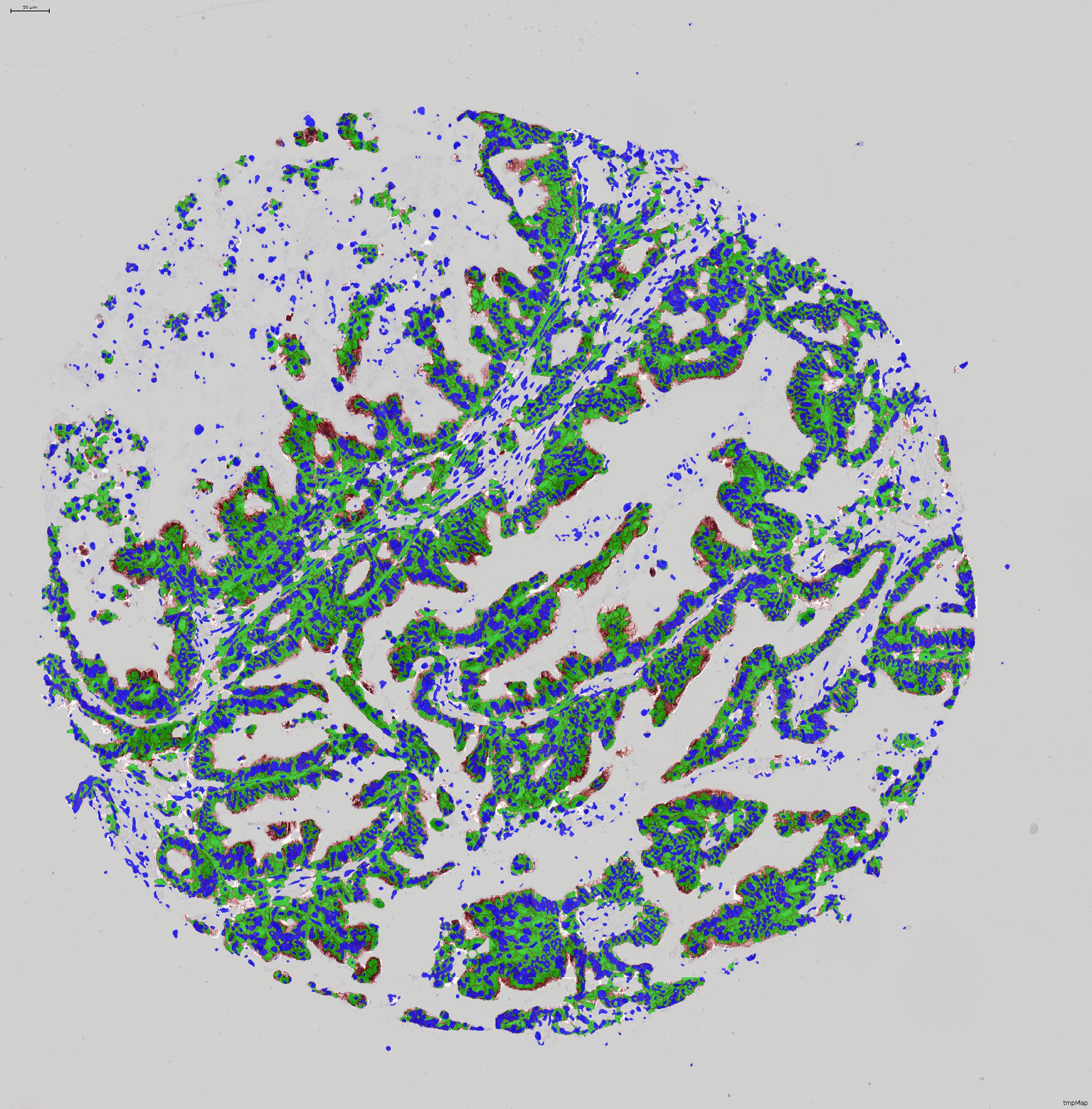

Supplement: Supplementary file 10 — Source Data for Figure 2 [file EMMM-12-e12010-s009.zip › Fig2A/Fig2_TMA94yFAK.20x.subsets.Core_10_4NucleusLevel_OverlayOutlines_.v2.jpg]

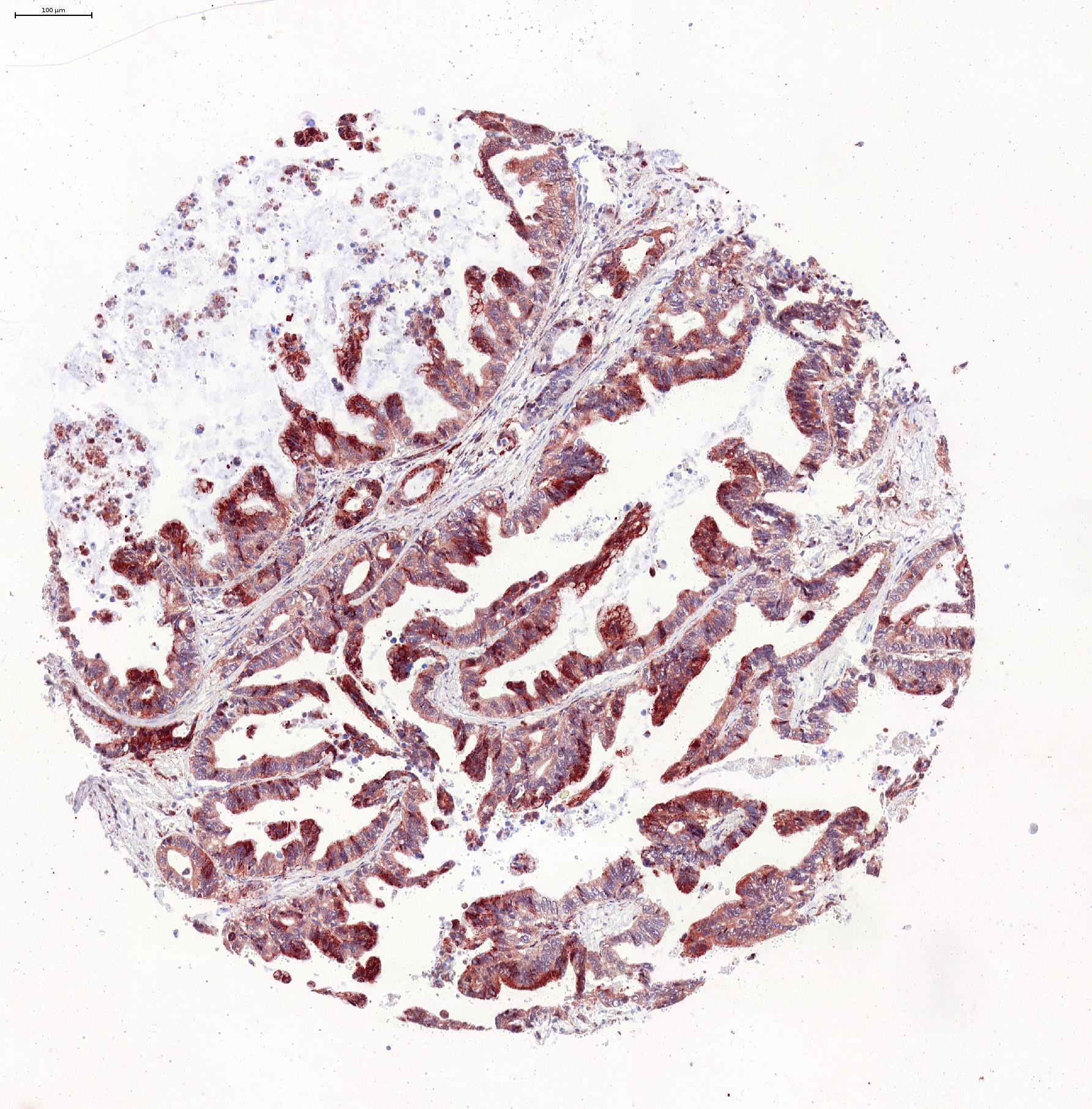

Supplement: Supplementary file 10 — Source Data for Figure 2 [file EMMM-12-e12010-s009.zip › Fig2A/Fig2_TMA94yFAK.20x.subsets.Core_10_ROI_Original_.v2.jpg]

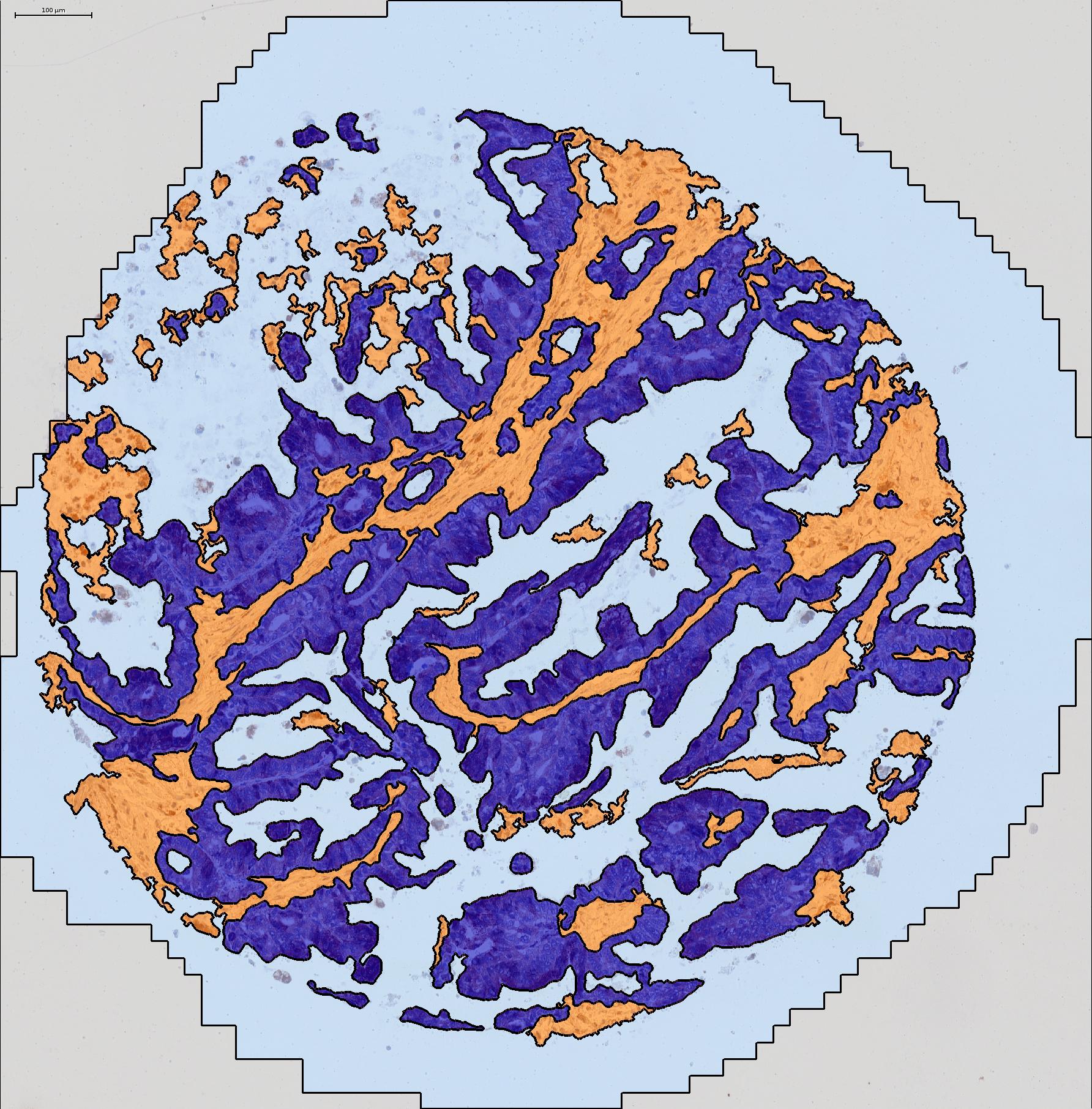

Supplement: Supplementary file 10 — Source Data for Figure 2 [file EMMM-12-e12010-s009.zip › Fig2A/Fig2_TMA94yFAK.20x.subsets.Core_10_ROI_OverlayOutlines_.v2.jpg]

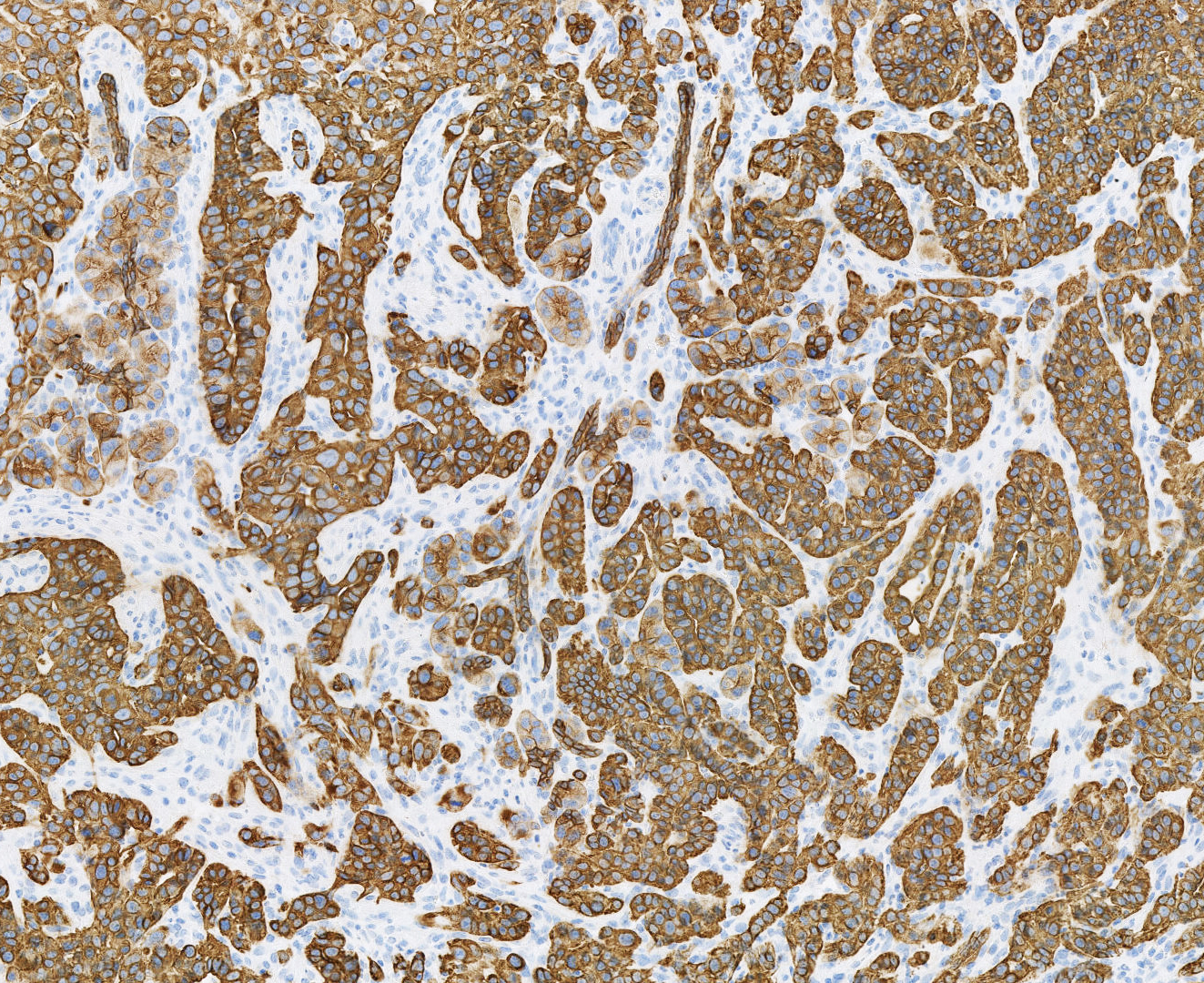

Supplement: Supplementary file 11 — Source Data for Figure 3 [file EMMM-12-e12010-s010.zip › Fig3b/Fig3B TC+FAK-KD fib CK19.jpg]

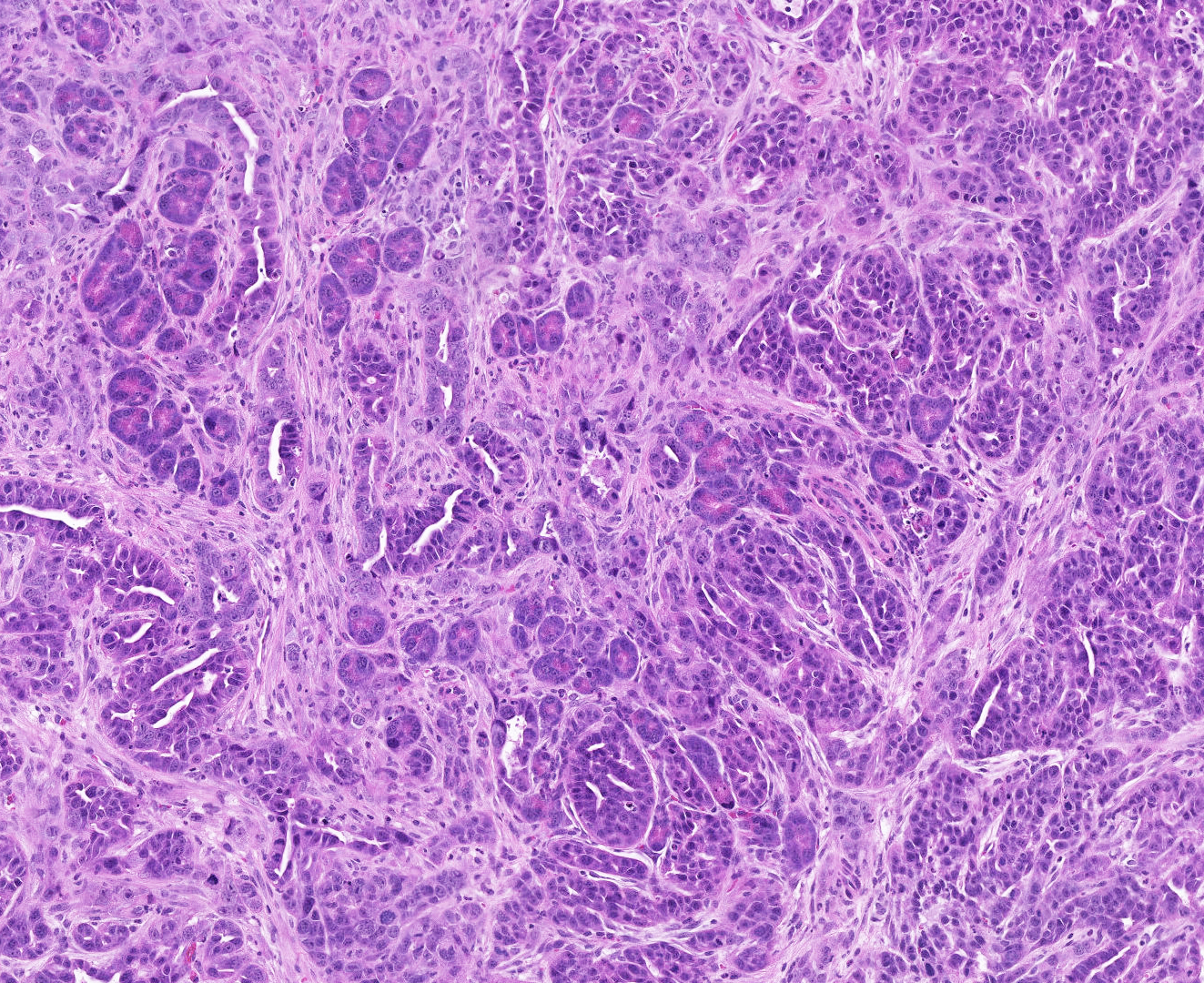

Supplement: Supplementary file 11 — Source Data for Figure 3 [file EMMM-12-e12010-s010.zip › Fig3b/Fig3B TC+FAK-KD fib HE.jpg]

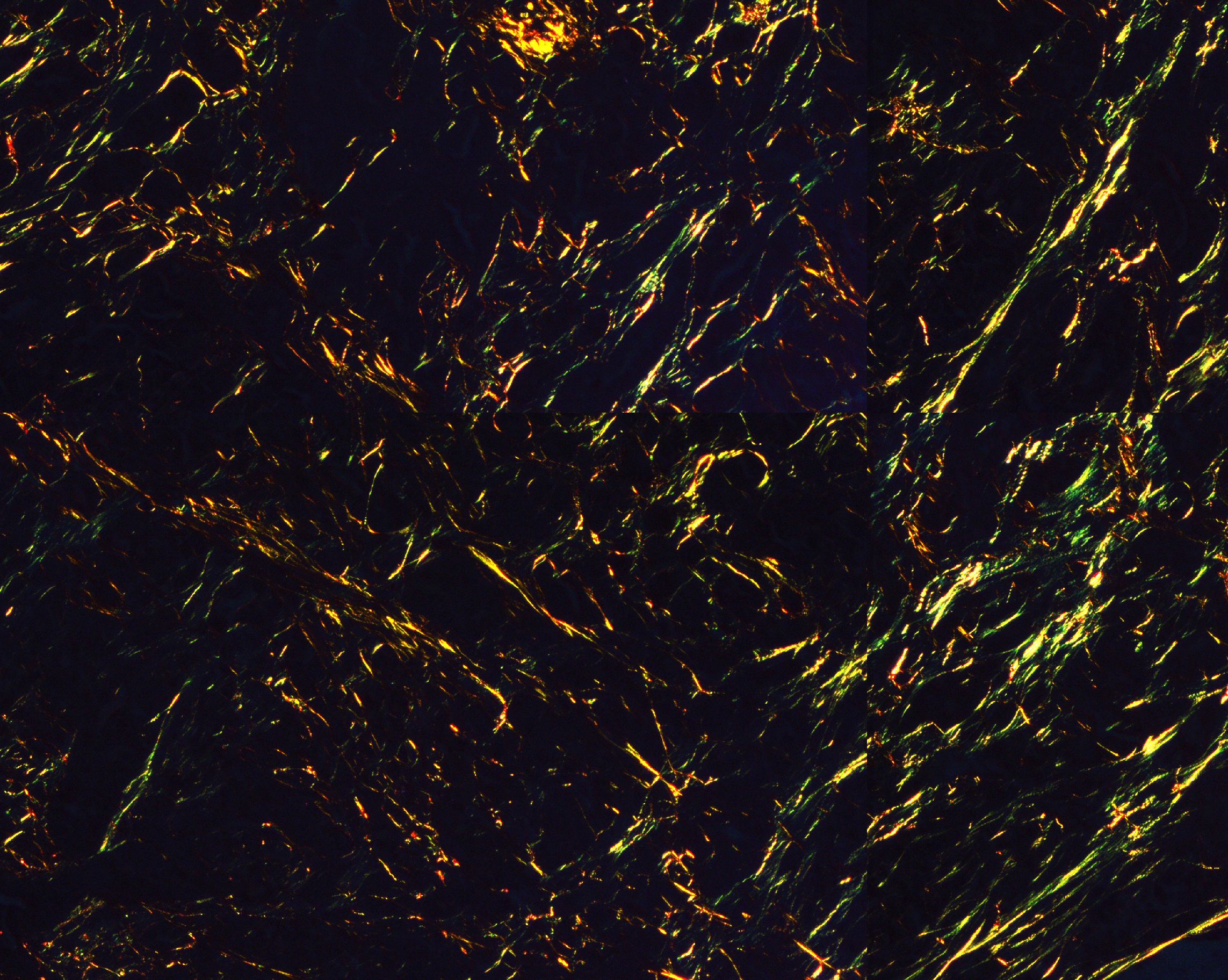

Supplement: Supplementary file 11 — Source Data for Figure 3 [file EMMM-12-e12010-s010.zip › Fig3b/Fig3B TC+FAK-KD fib sIRIUS POL2.jpg]

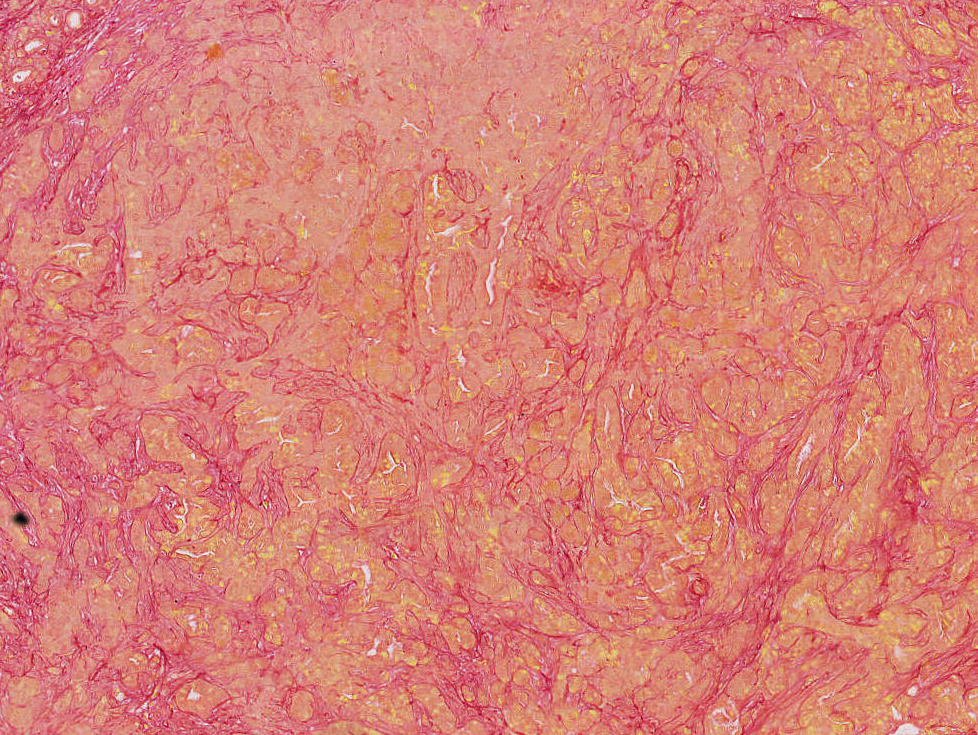

Supplement: Supplementary file 11 — Source Data for Figure 3 [file EMMM-12-e12010-s010.zip › Fig3b/Fig3B TC+FAK-KD fib Sirius.jpg]

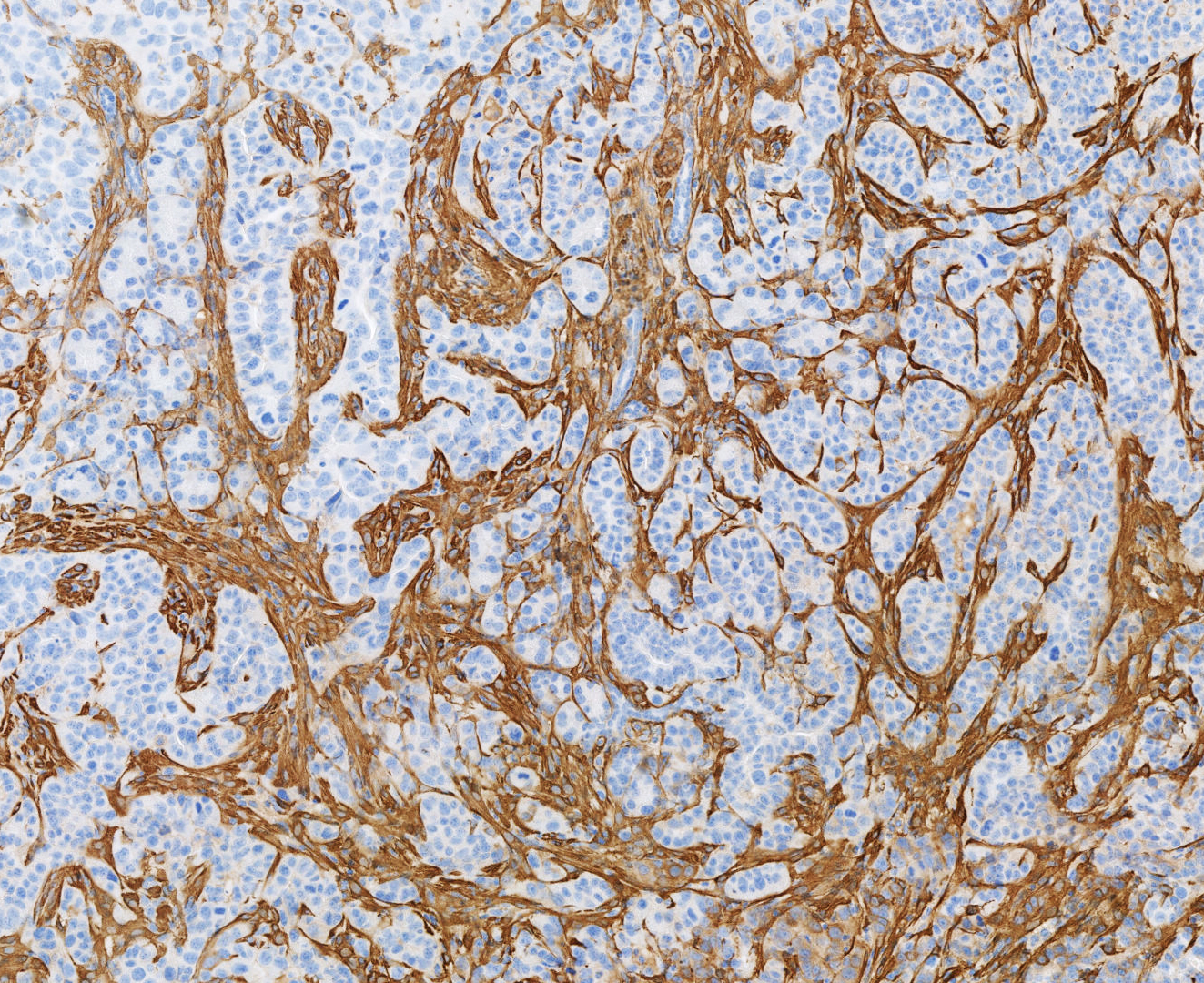

Supplement: Supplementary file 11 — Source Data for Figure 3 [file EMMM-12-e12010-s010.zip › Fig3b/Fig3B TC+FAK-KD fib SMA.jpg]

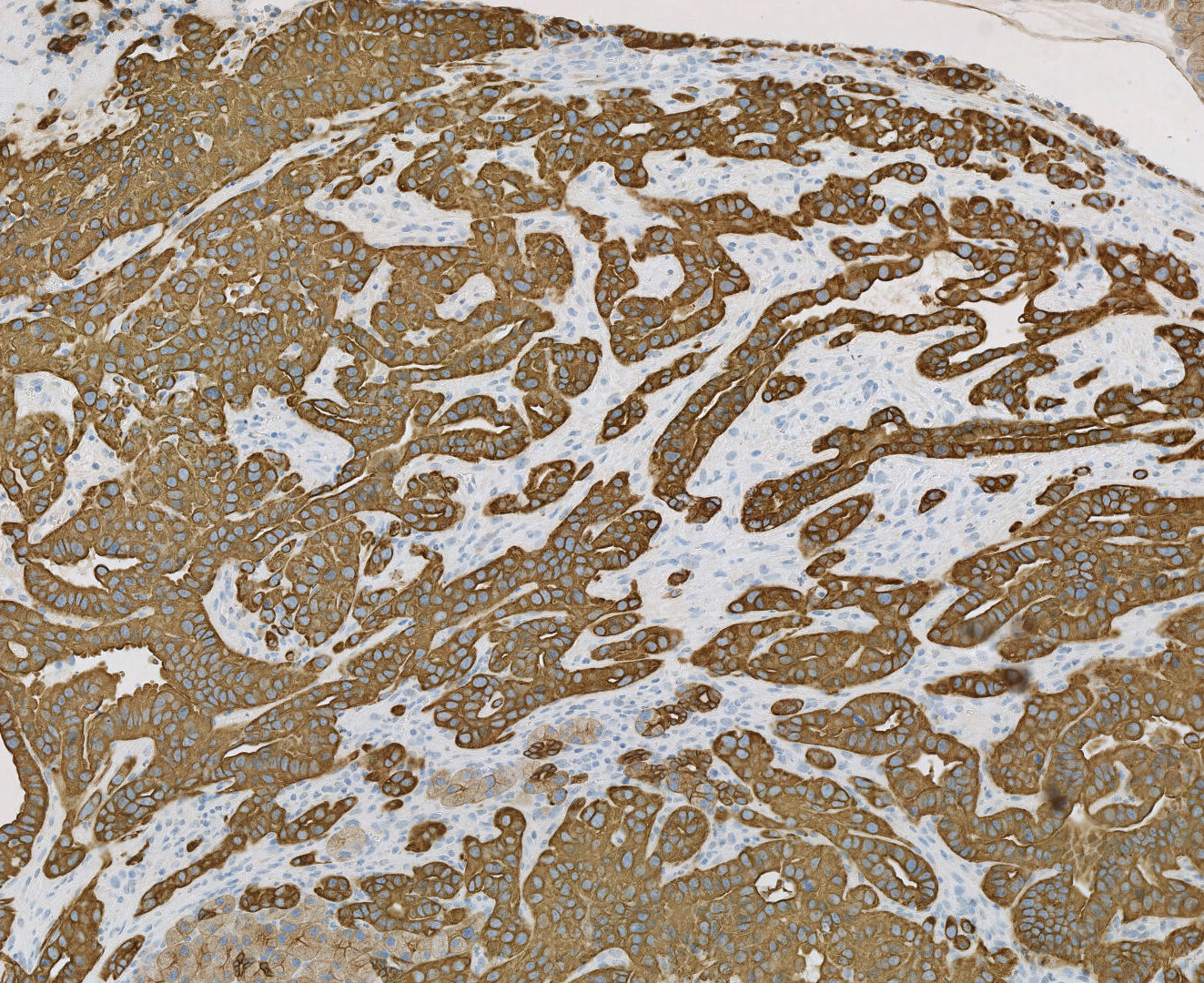

Supplement: Supplementary file 11 — Source Data for Figure 3 [file EMMM-12-e12010-s010.zip › Fig3b/Fig3B TC+FAK-WT fib CK19.jpg]

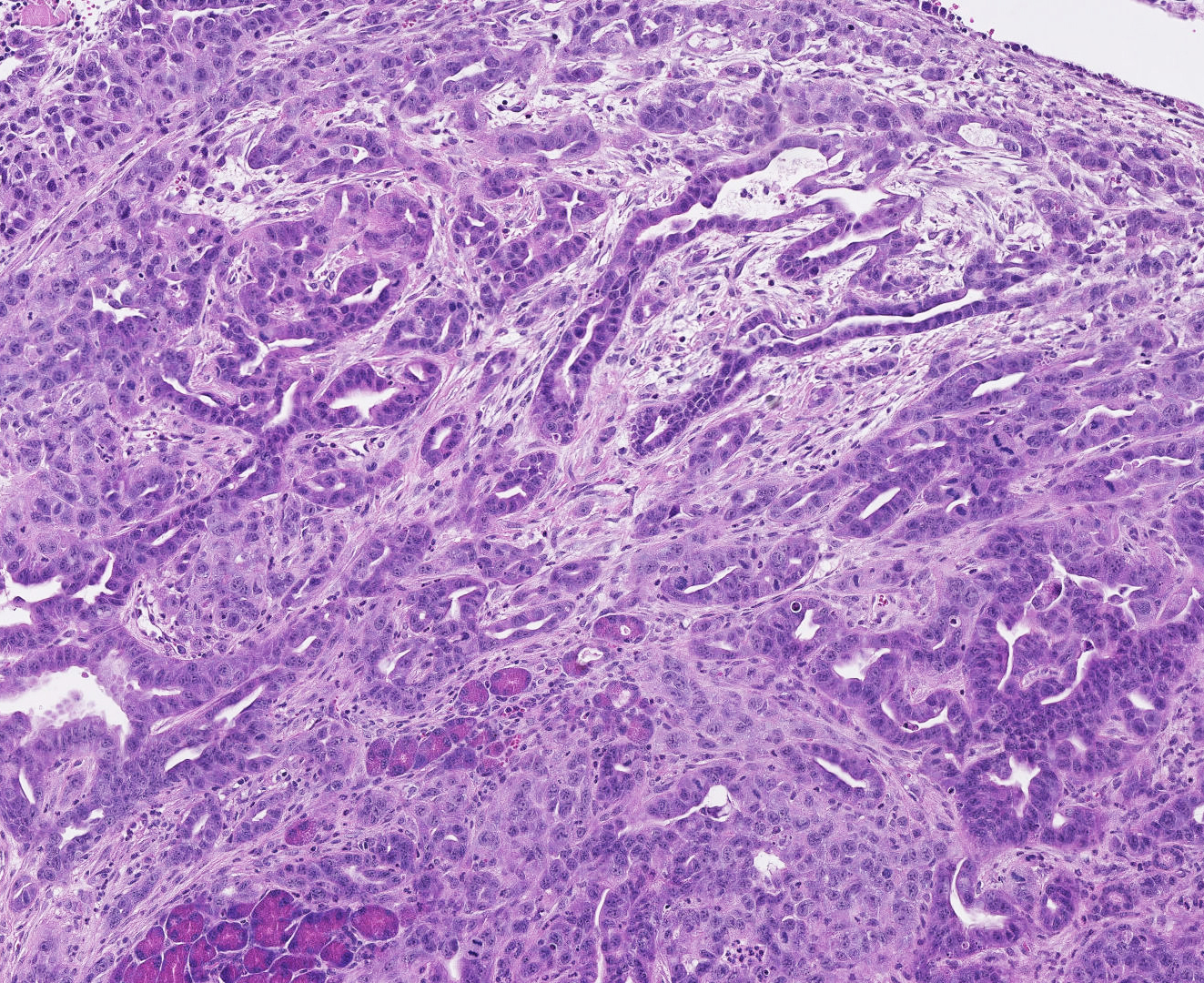

Supplement: Supplementary file 11 — Source Data for Figure 3 [file EMMM-12-e12010-s010.zip › Fig3b/Fig3B TC+FAK-WT fib HE.jpg]

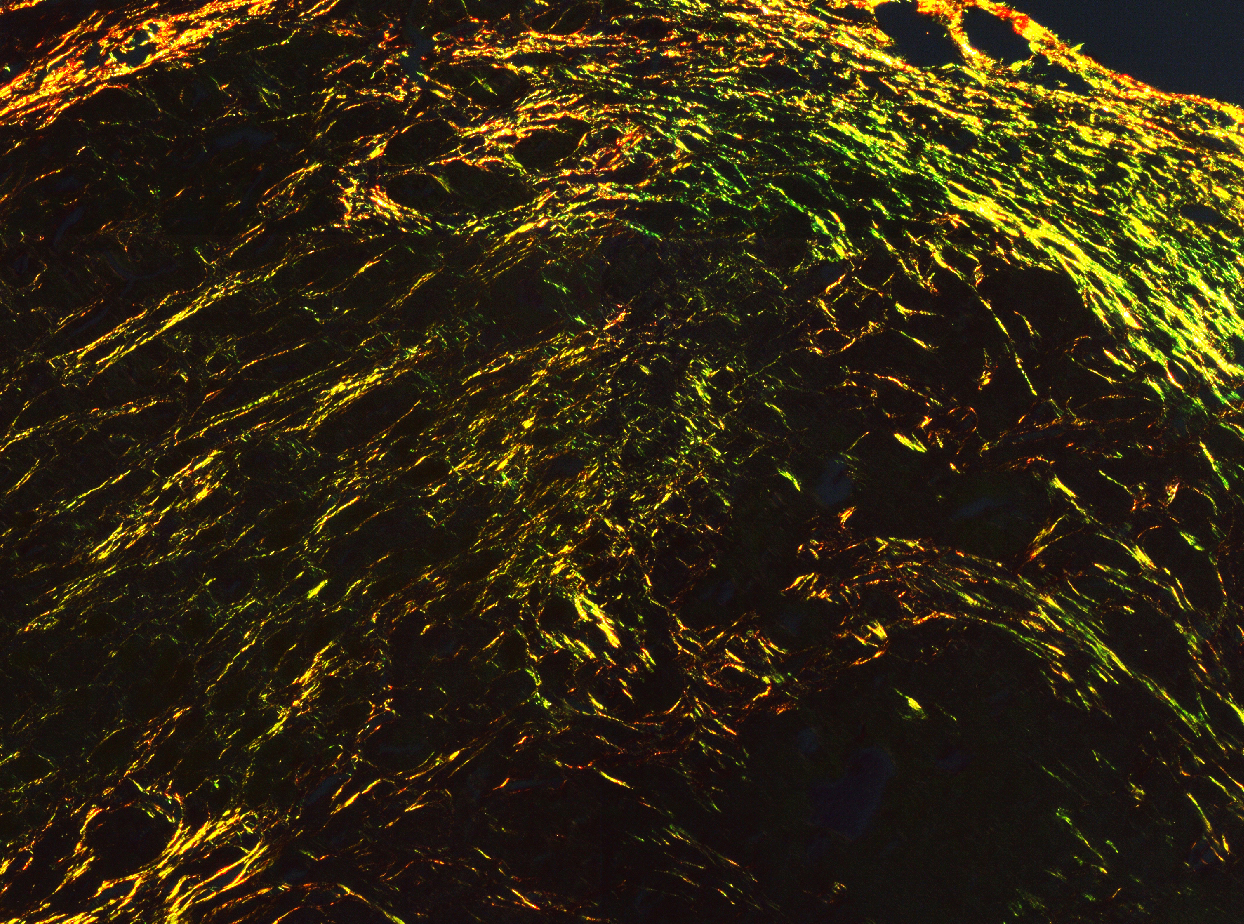

Supplement: Supplementary file 11 — Source Data for Figure 3 [file EMMM-12-e12010-s010.zip › Fig3b/Fig3B TC+FAK-WT fib sIRIUS POL2.jpg]

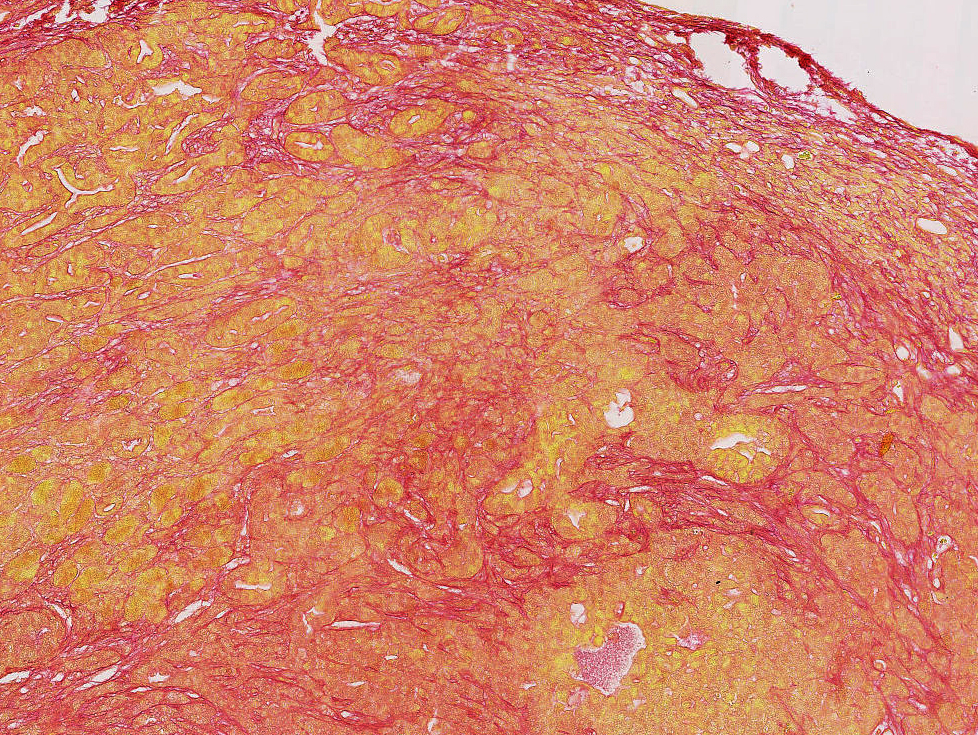

Supplement: Supplementary file 11 — Source Data for Figure 3 [file EMMM-12-e12010-s010.zip › Fig3b/Fig3B TC+FAK-WT fib sirius.jpg]

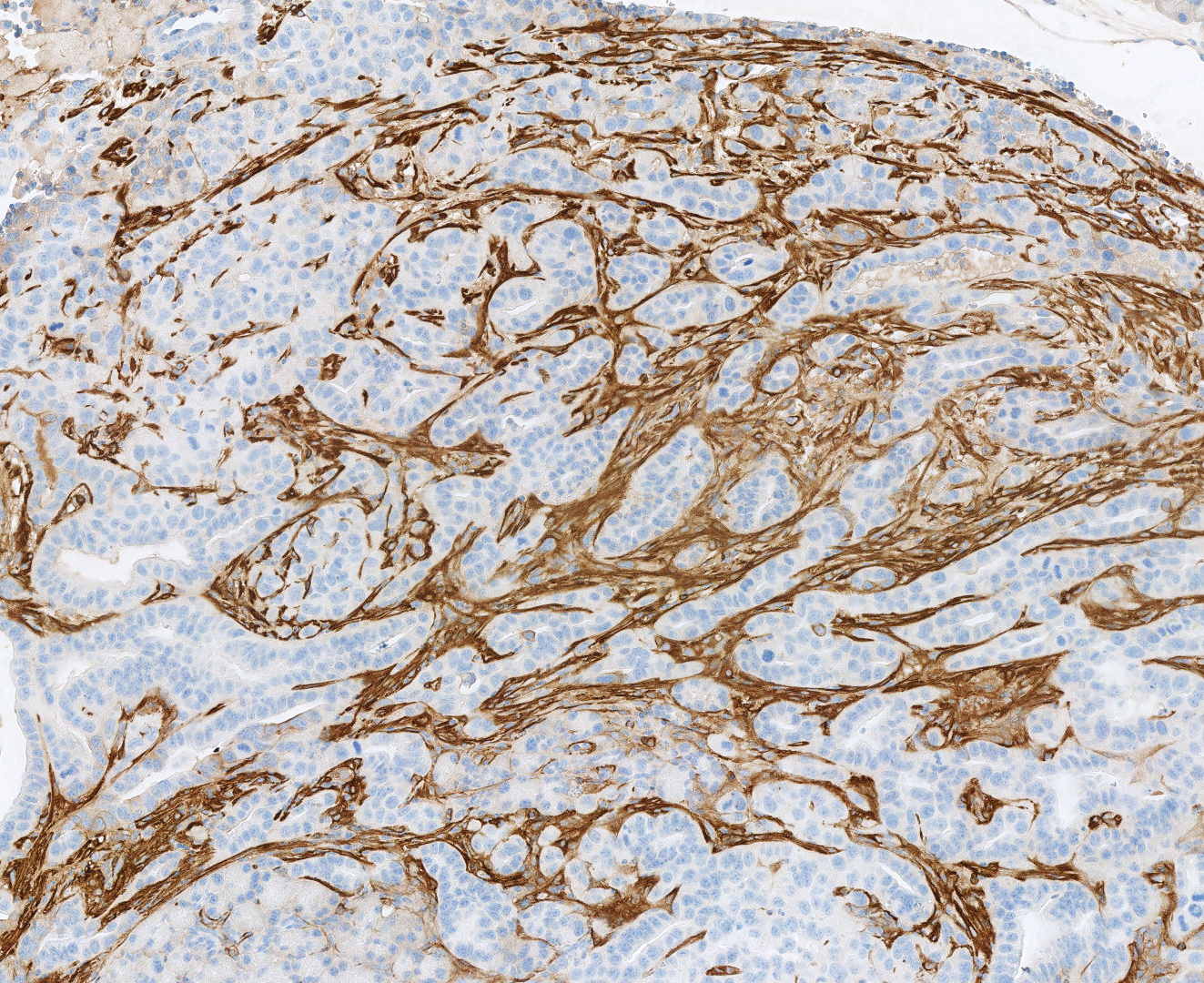

Supplement: Supplementary file 11 — Source Data for Figure 3 [file EMMM-12-e12010-s010.zip › Fig3b/Fig3B TC+FAK-WT fib sma.jpg]

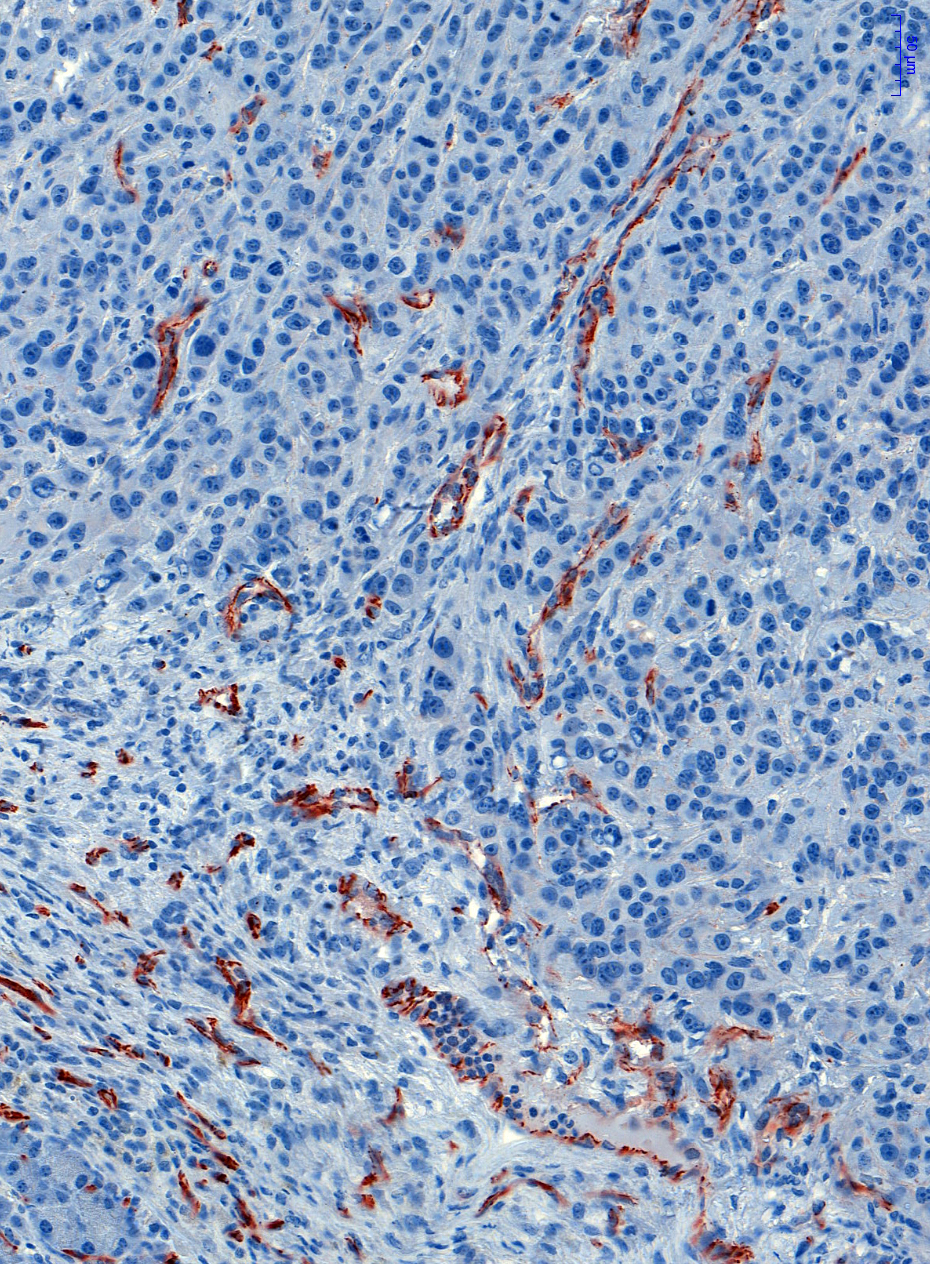

Supplement: Supplementary file 11 — Source Data for Figure 3 [file EMMM-12-e12010-s010.zip › Fig3D/Fig3D KD1 TC CD31_20.0.jpg]

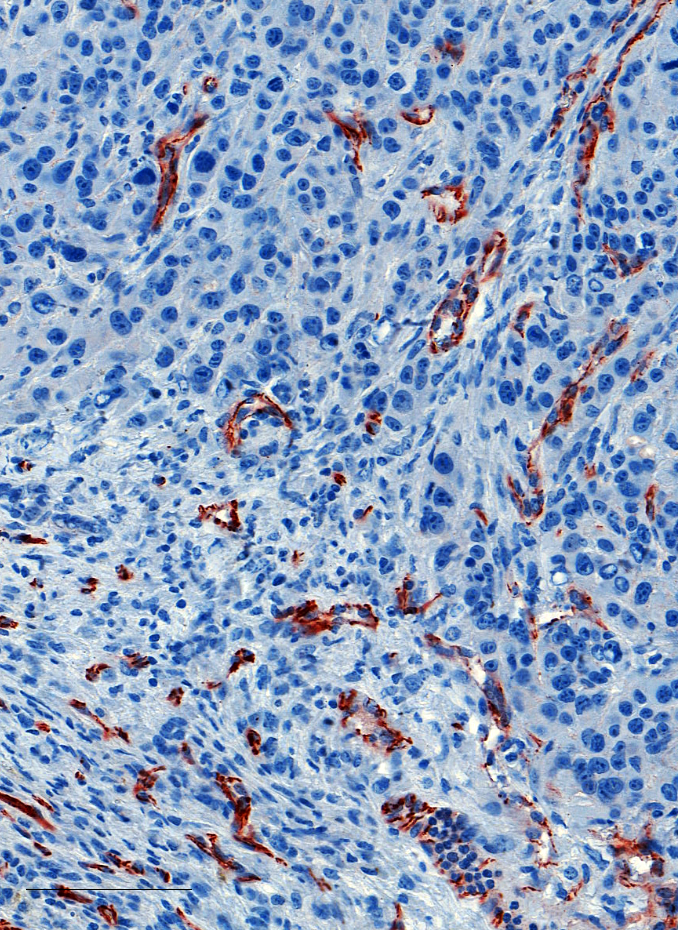

Supplement: Supplementary file 11 — Source Data for Figure 3 [file EMMM-12-e12010-s010.zip › Fig3D/Fig3D KD1 TC crop.tif]

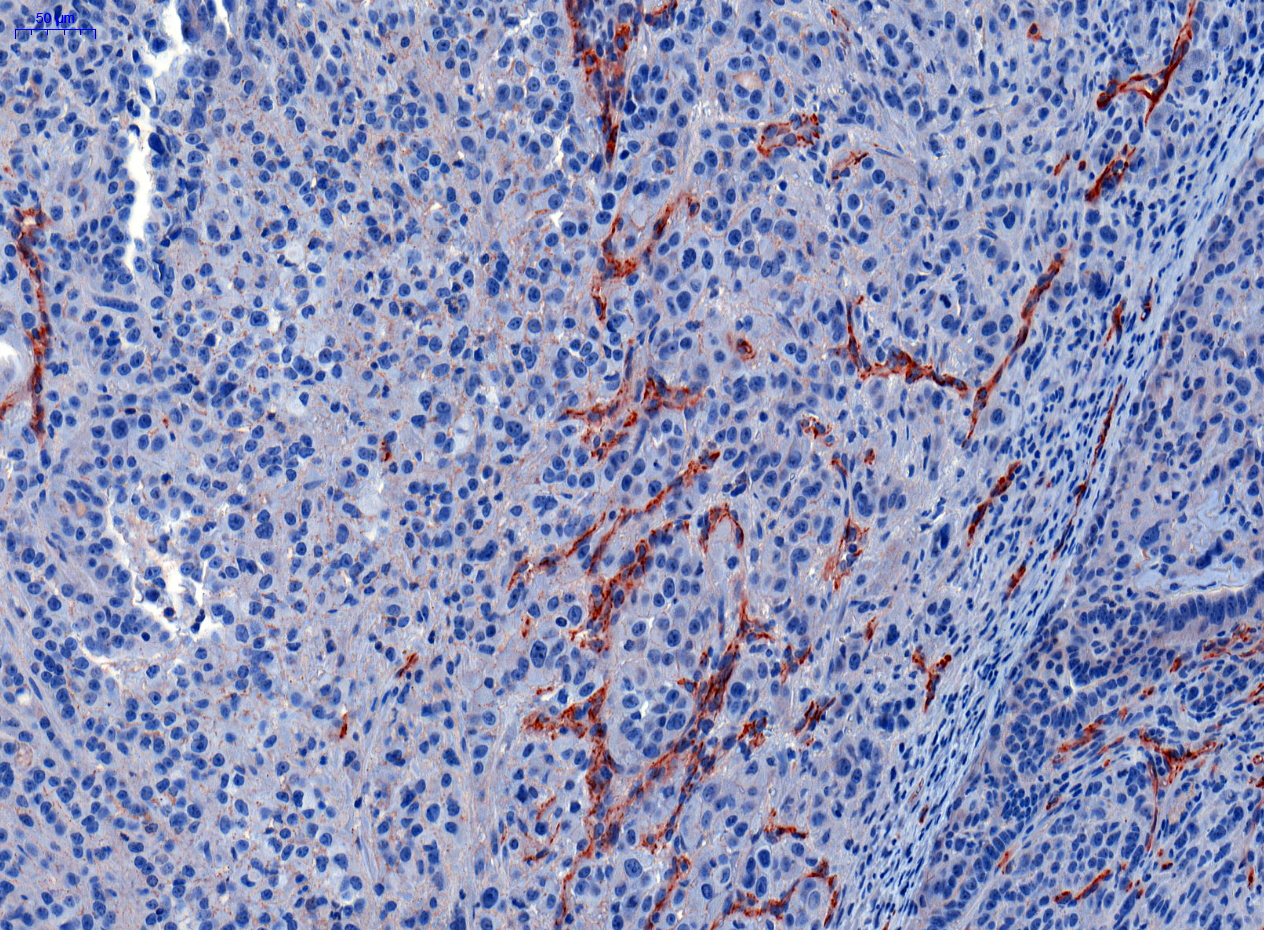

Supplement: Supplementary file 11 — Source Data for Figure 3 [file EMMM-12-e12010-s010.zip › Fig3D/Fig3D WT TC CD31_20.0.jpg]

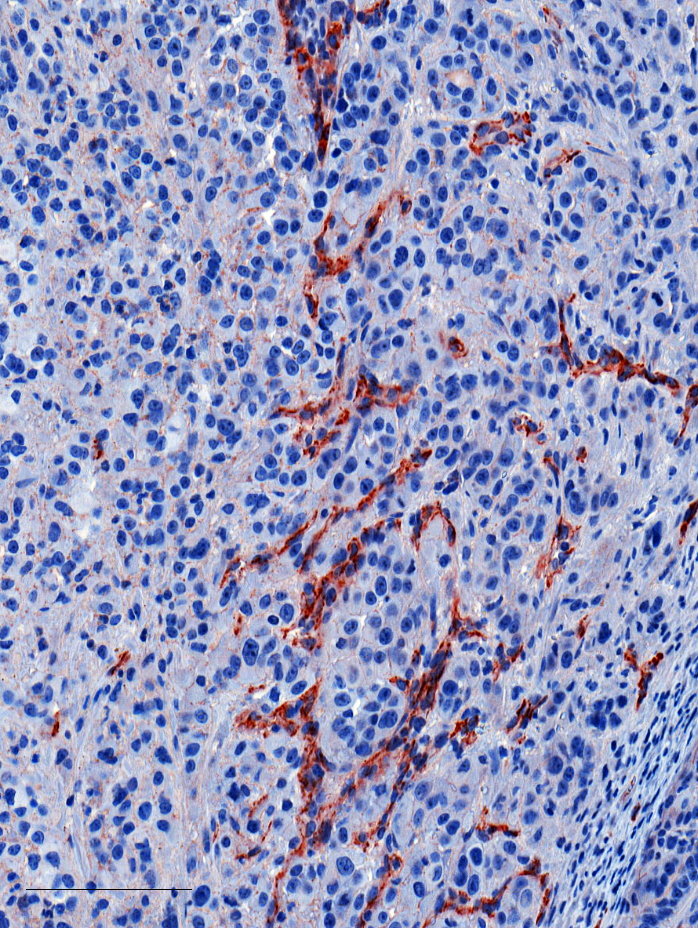

Supplement: Supplementary file 11 — Source Data for Figure 3 [file EMMM-12-e12010-s010.zip › Fig3D/Fig3D WT TC crop.tif]

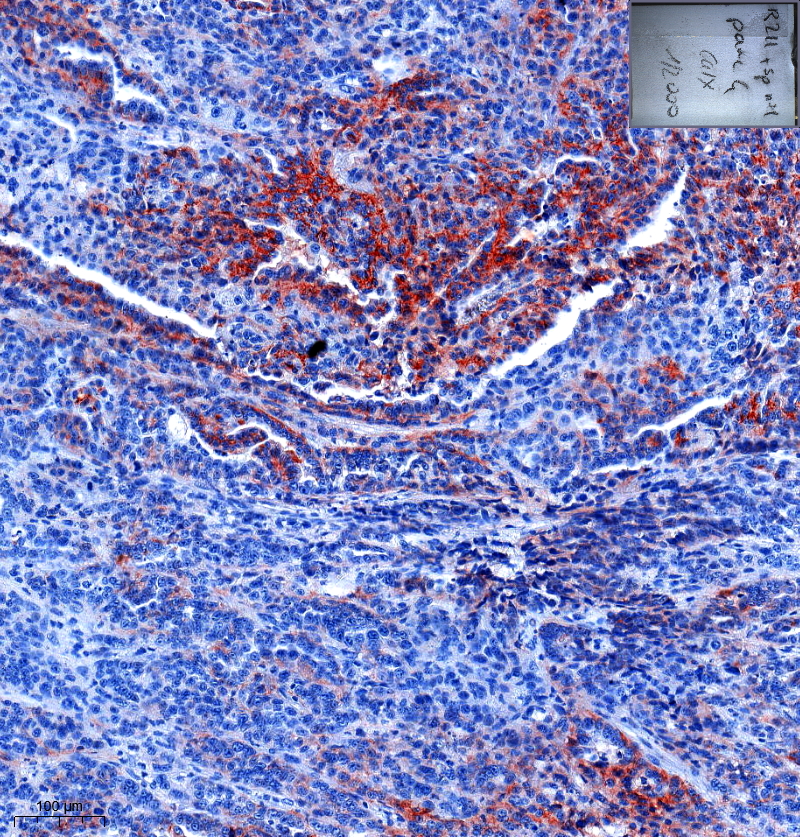

Supplement: Supplementary file 11 — Source Data for Figure 3 [file EMMM-12-e12010-s010.zip › fIG3E/Fig3E KD1 TC CA9_9.0x.jpg]

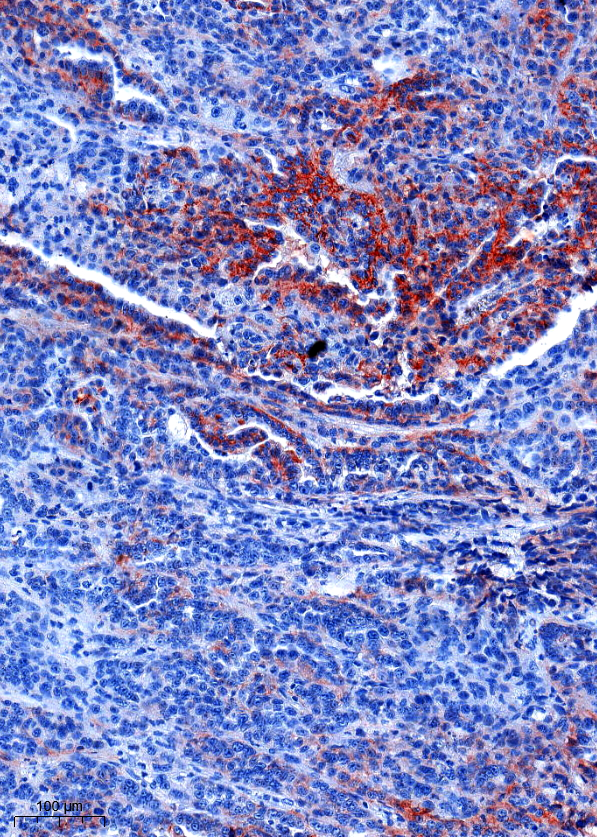

Supplement: Supplementary file 11 — Source Data for Figure 3 [file EMMM-12-e12010-s010.zip › fIG3E/Fig3E KD1 TC CA9CROP.tif]

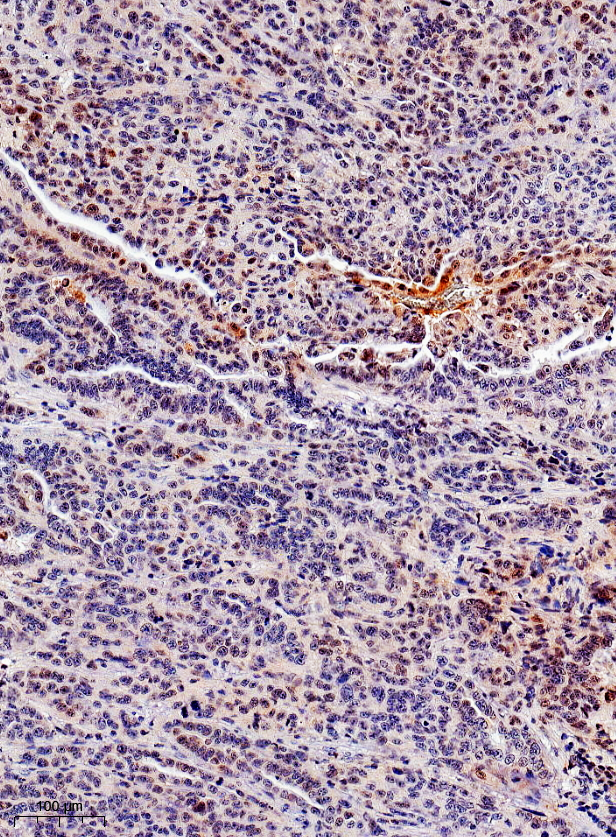

Supplement: Supplementary file 11 — Source Data for Figure 3 [file EMMM-12-e12010-s010.zip › fIG3E/Fig3E KD1 TC HIF CROP.tif]

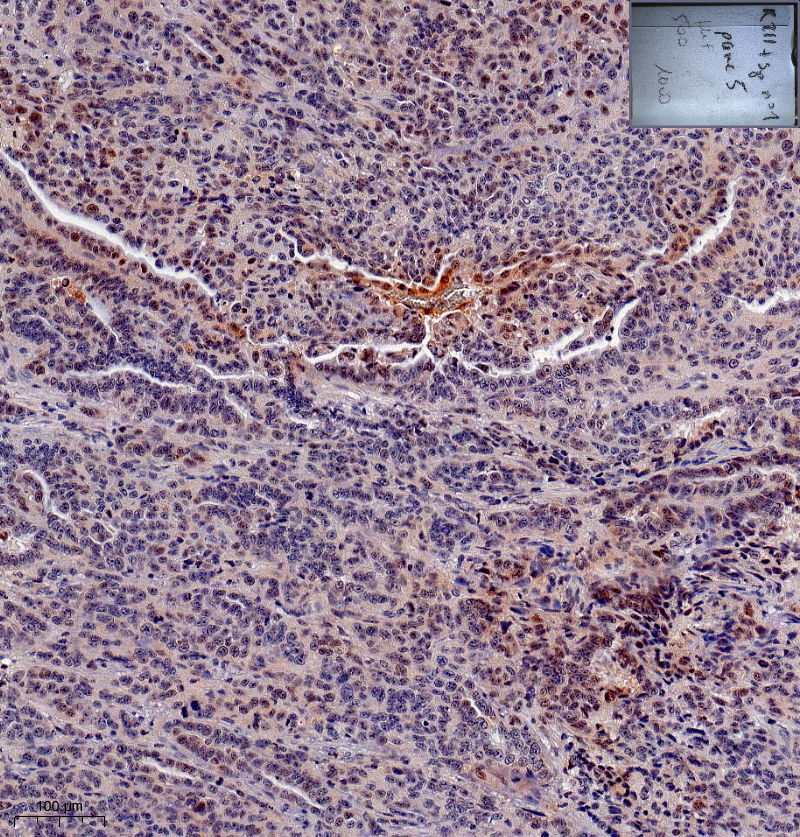

Supplement: Supplementary file 11 — Source Data for Figure 3 [file EMMM-12-e12010-s010.zip › fIG3E/Fig3E KD1 TC HIF_9.0x.jpg]

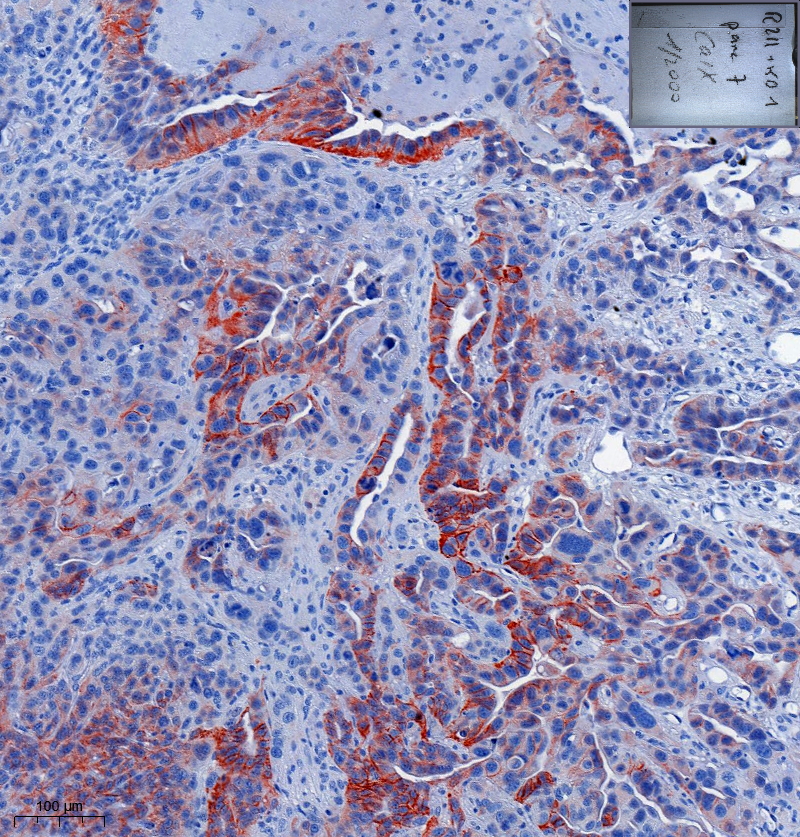

Supplement: Supplementary file 11 — Source Data for Figure 3 [file EMMM-12-e12010-s010.zip › fIG3E/Fig3E WT TC CA9_9.0x .jpg]

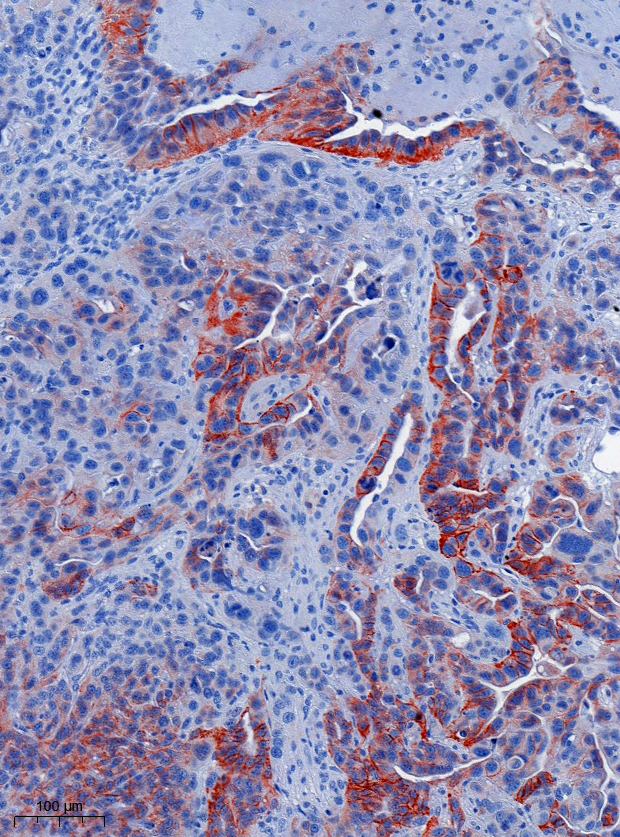

Supplement: Supplementary file 11 — Source Data for Figure 3 [file EMMM-12-e12010-s010.zip › fIG3E/Fig3E WT TC CA9CROP .tif]

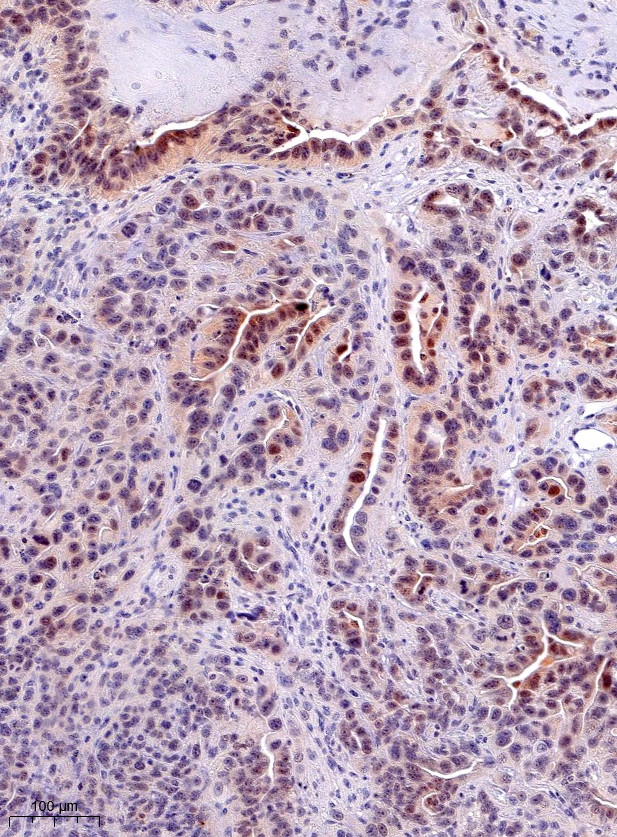

Supplement: Supplementary file 11 — Source Data for Figure 3 [file EMMM-12-e12010-s010.zip › fIG3E/Fig3E WT TC HIF CROP .tif]

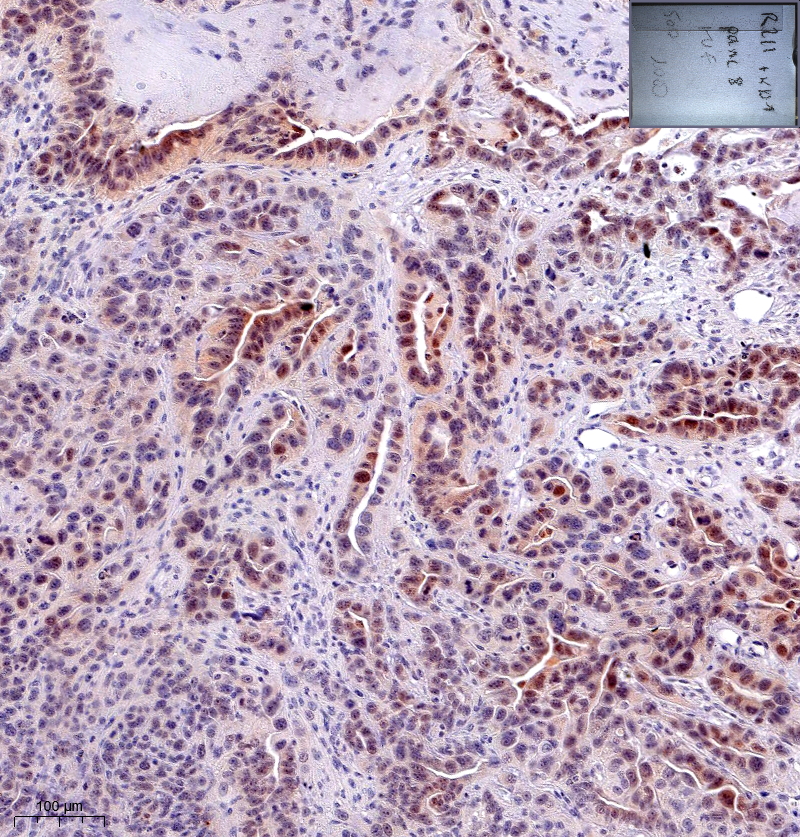

Supplement: Supplementary file 11 — Source Data for Figure 3 [file EMMM-12-e12010-s010.zip › fIG3E/Fig3E WT TC HIF1a_9.0x.jpg]

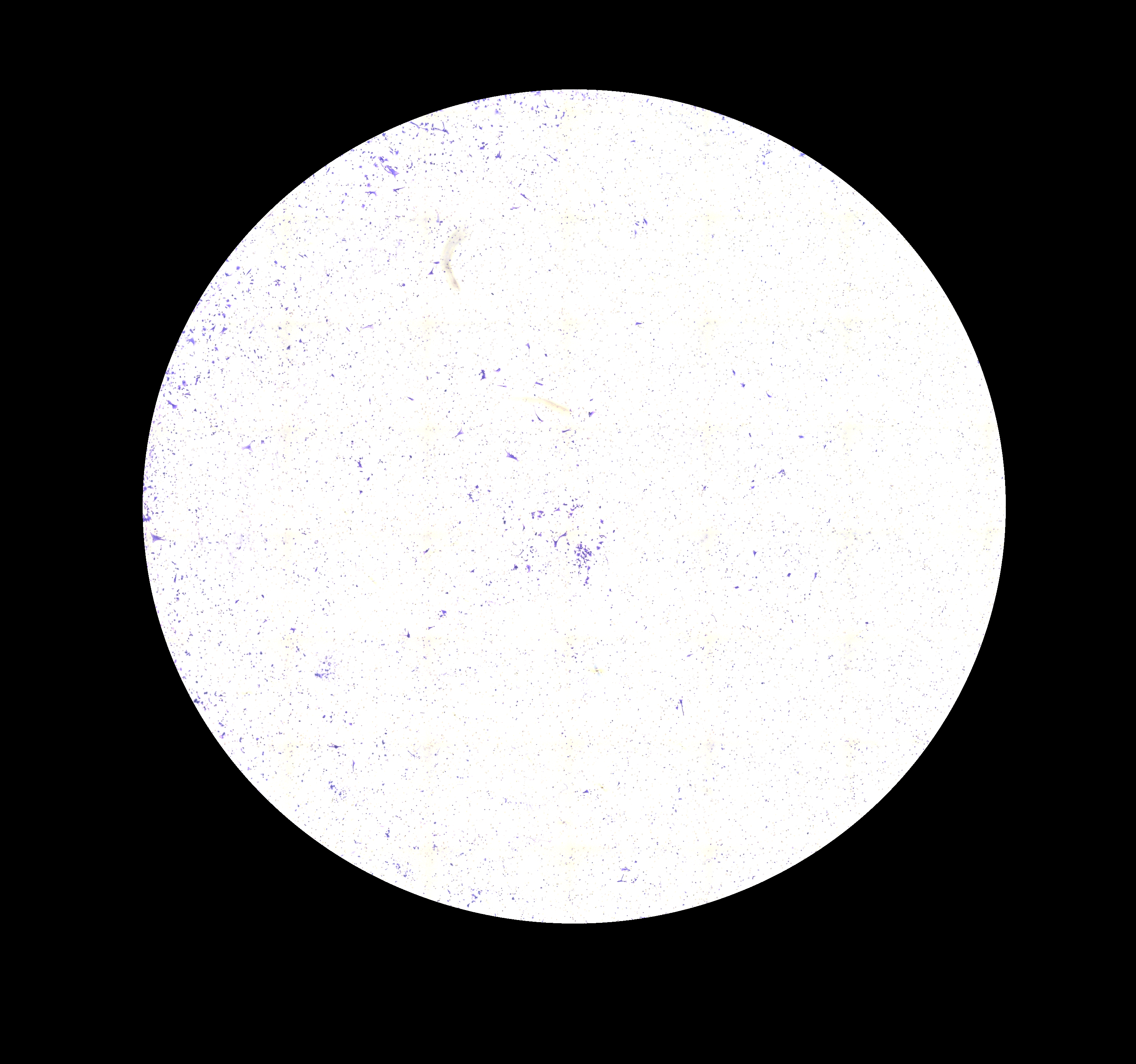

Supplement: Supplementary file 12 — Source Data for Figure 4 [file EMMM-12-e12010-s011.zip › Fig4E/Fig4E CM hCAF 24h M1.tif]

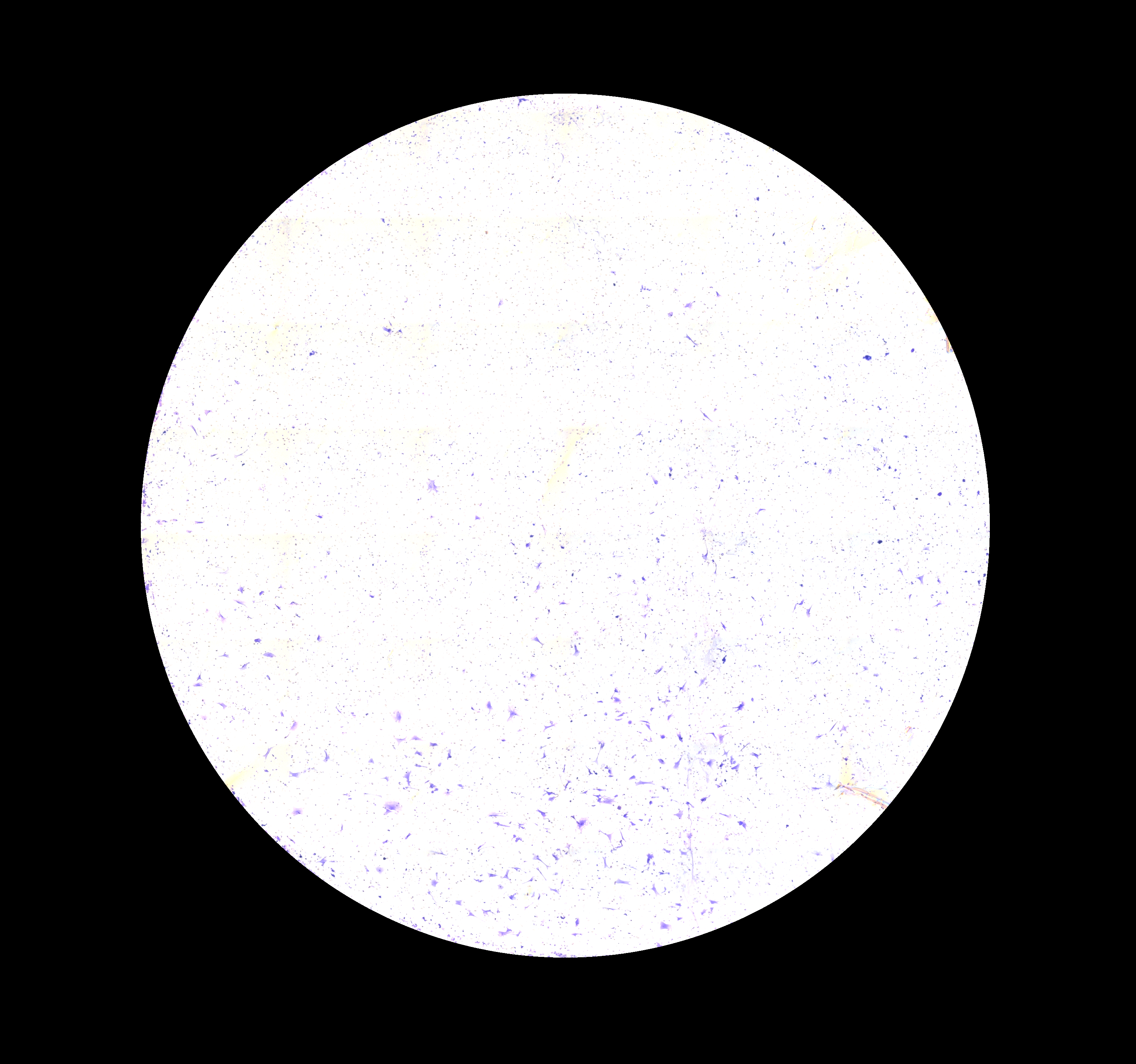

Supplement: Supplementary file 12 — Source Data for Figure 4 [file EMMM-12-e12010-s011.zip › Fig4E/Fig4E CM hCAF 24h M2.tif]

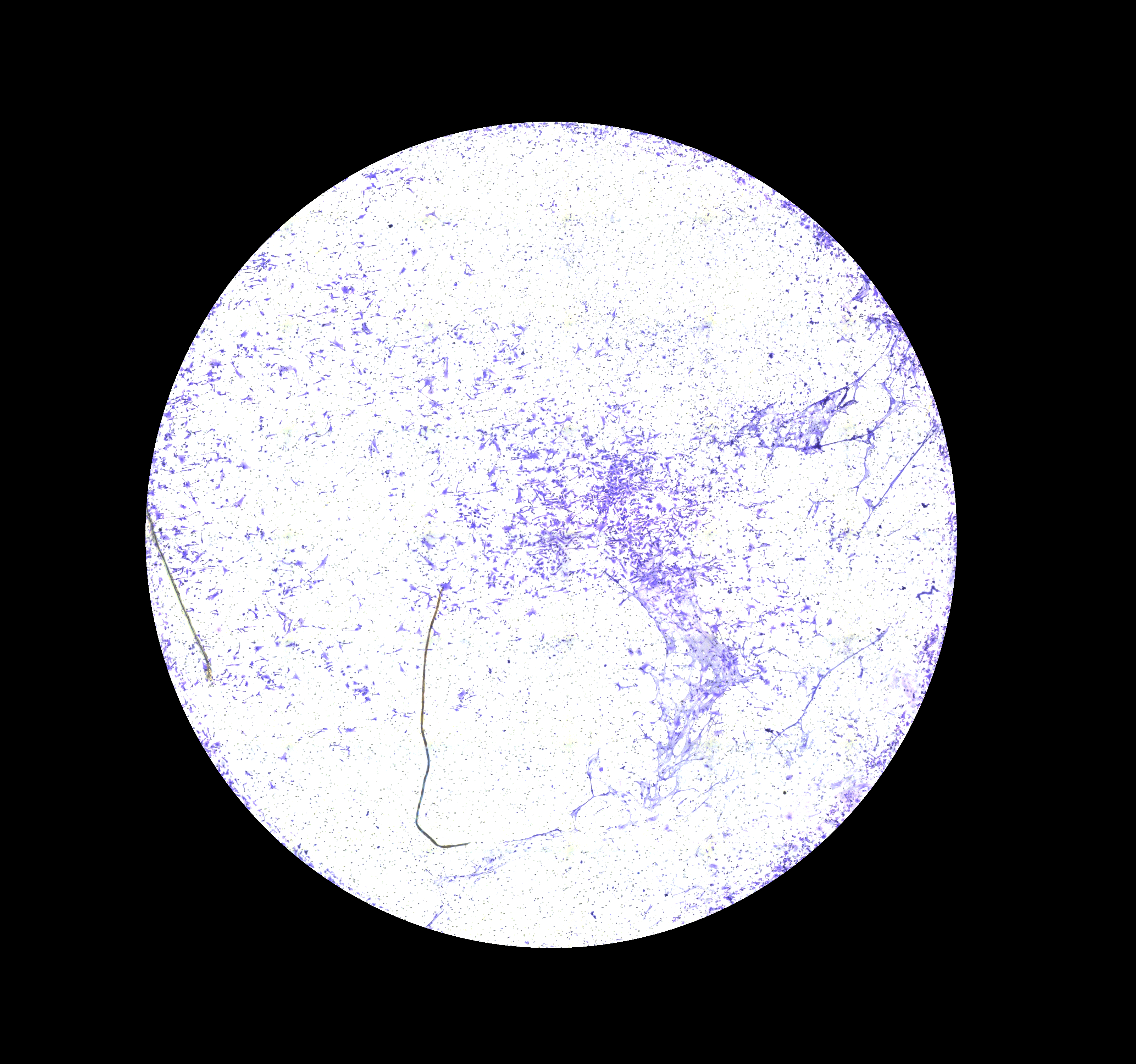

Supplement: Supplementary file 12 — Source Data for Figure 4 [file EMMM-12-e12010-s011.zip › Fig4E/Fig4E CM hCAF 48h M1.tif]

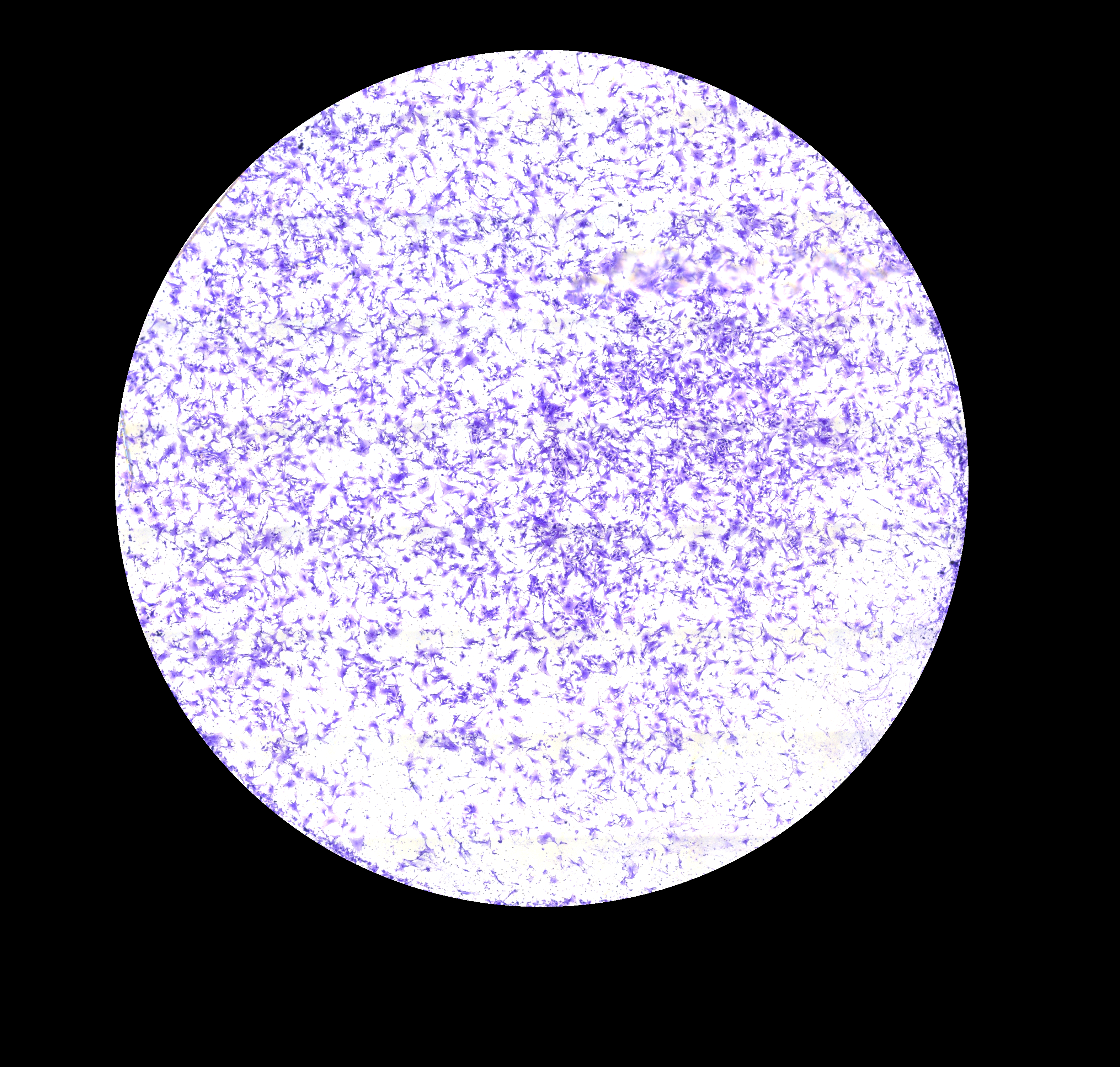

Supplement: Supplementary file 12 — Source Data for Figure 4 [file EMMM-12-e12010-s011.zip › Fig4E/Fig4E CM hCAF 48h M2.tif]

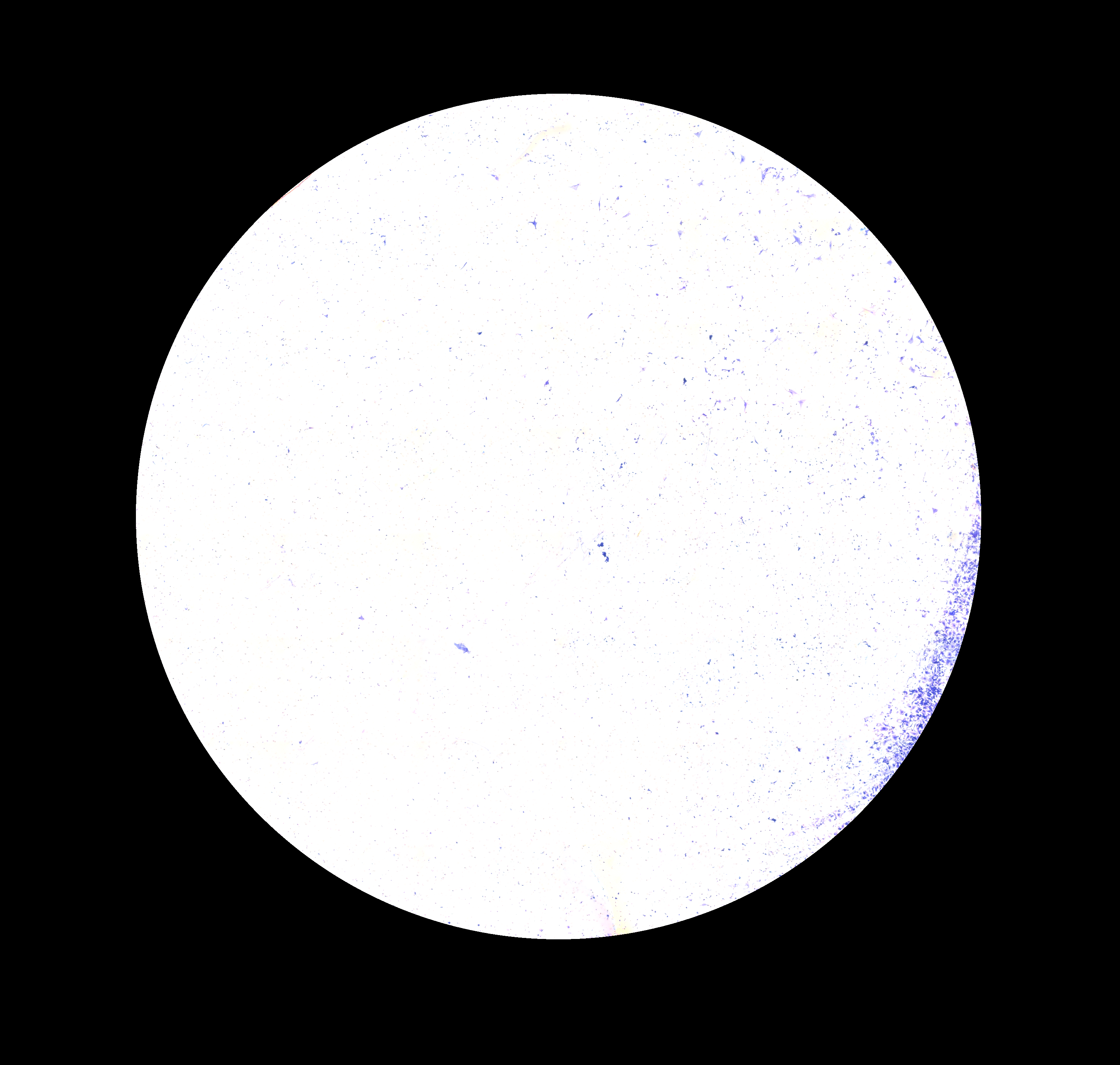

Supplement: Supplementary file 12 — Source Data for Figure 4 [file EMMM-12-e12010-s011.zip › Fig4E/Fig4E CM hCAF FAK-I 24h M1.tif]

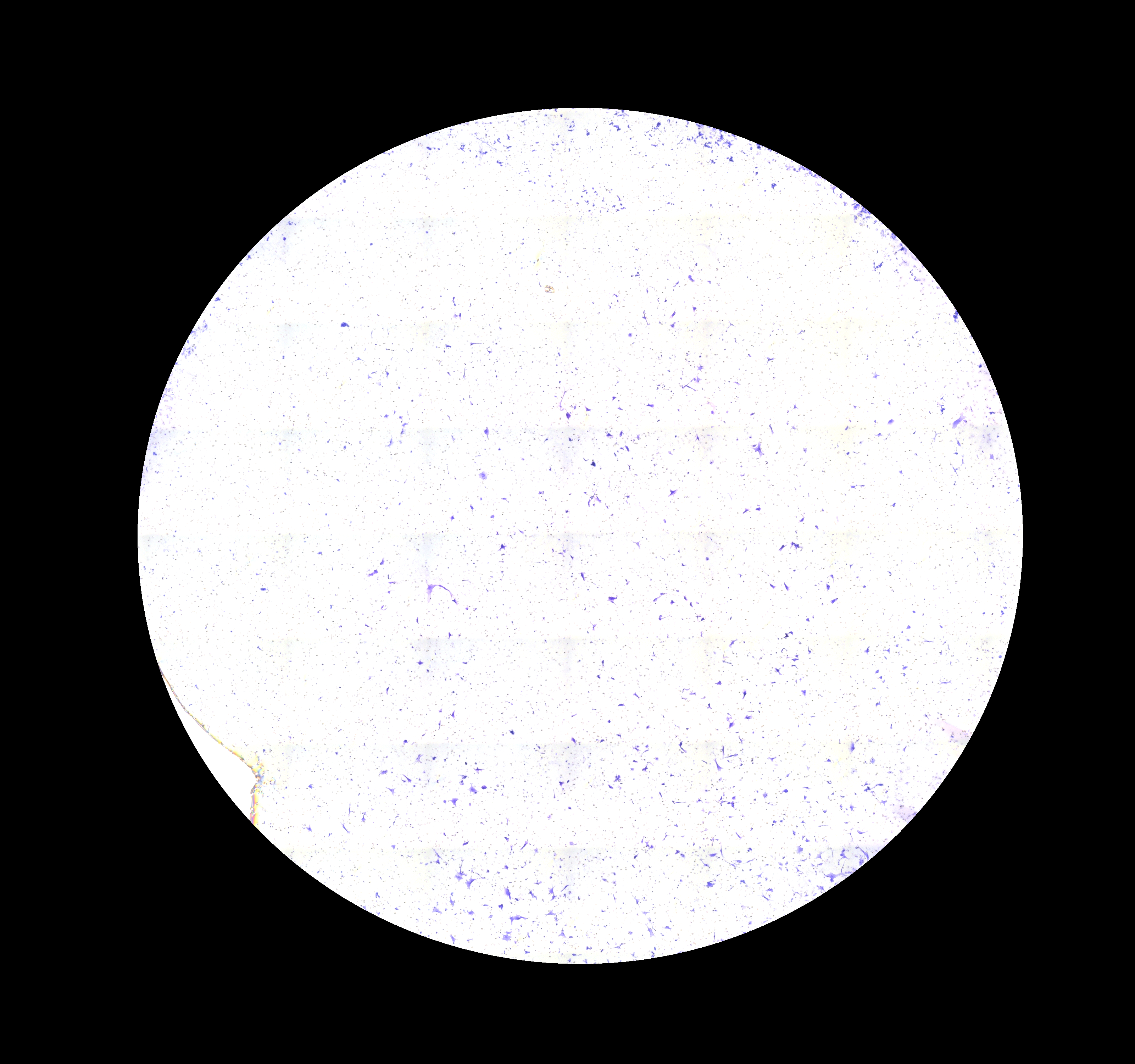

Supplement: Supplementary file 12 — Source Data for Figure 4 [file EMMM-12-e12010-s011.zip › Fig4E/Fig4E CM hCAF FAK-I 24h M2.tif]

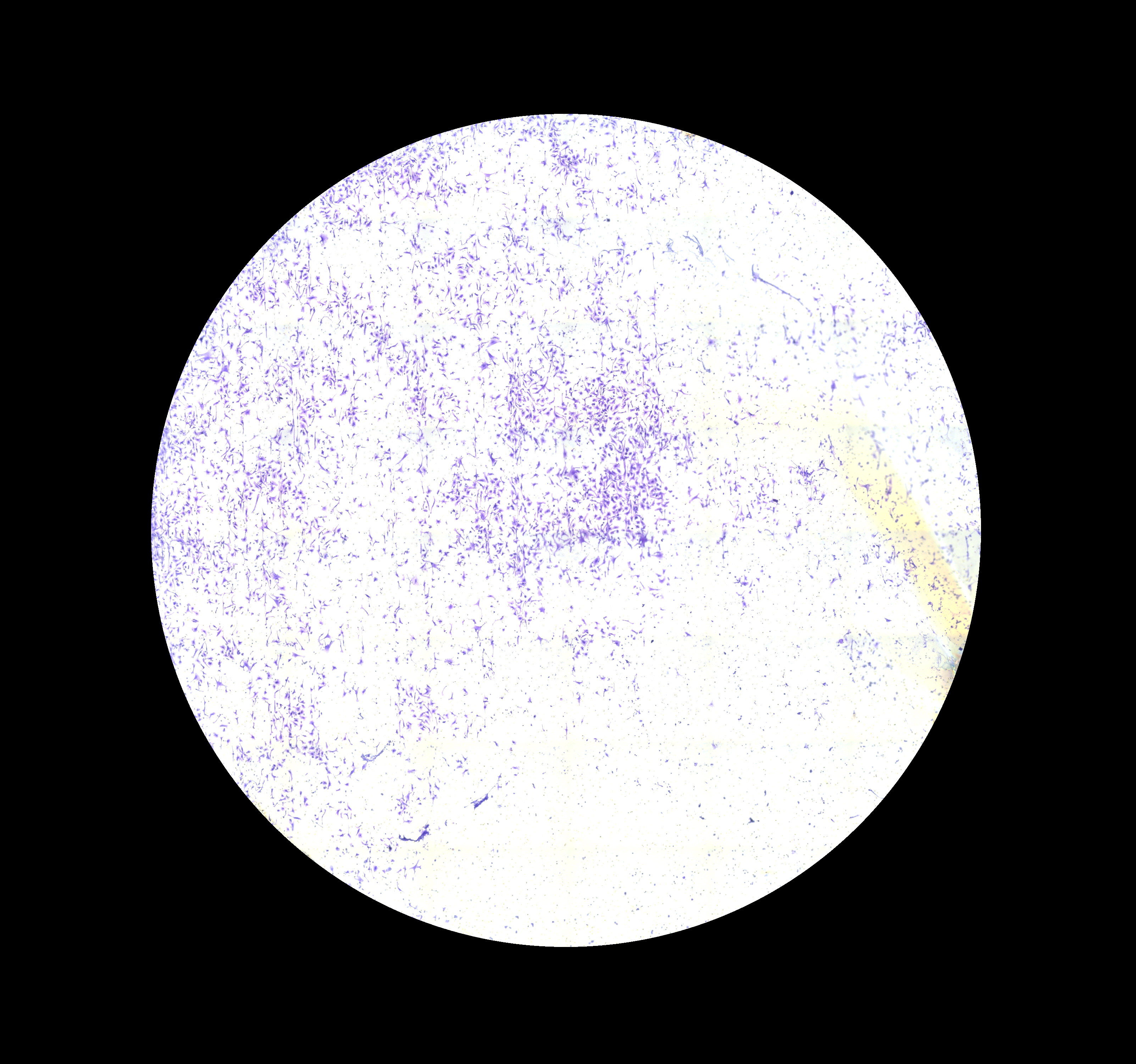

Supplement: Supplementary file 12 — Source Data for Figure 4 [file EMMM-12-e12010-s011.zip › Fig4E/Fig4E CM hCAF FAK-I 48h M1.tif]

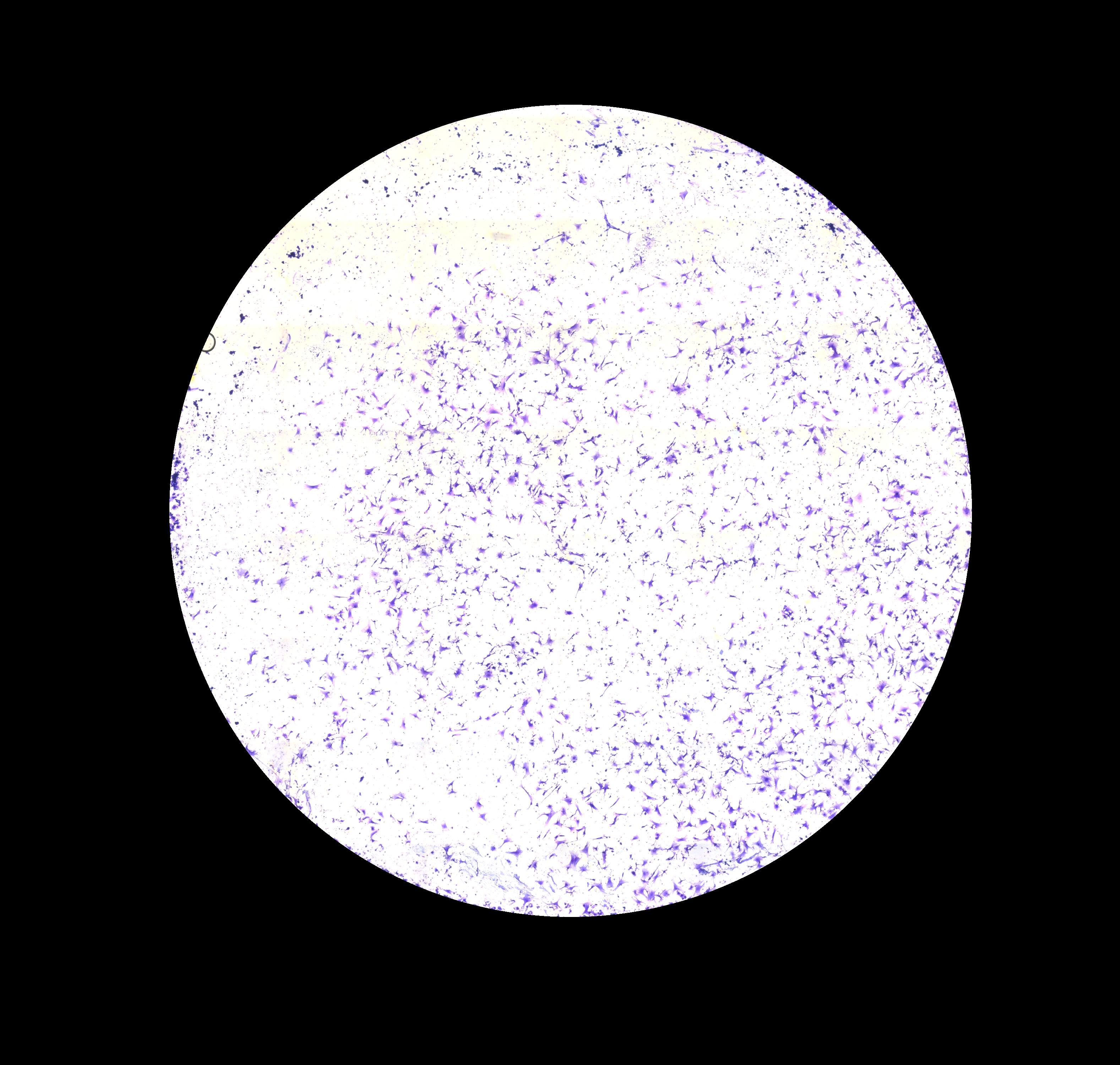

Supplement: Supplementary file 12 — Source Data for Figure 4 [file EMMM-12-e12010-s011.zip › Fig4E/Fig4E CM hCAF FAK-I 48h M2.tif]

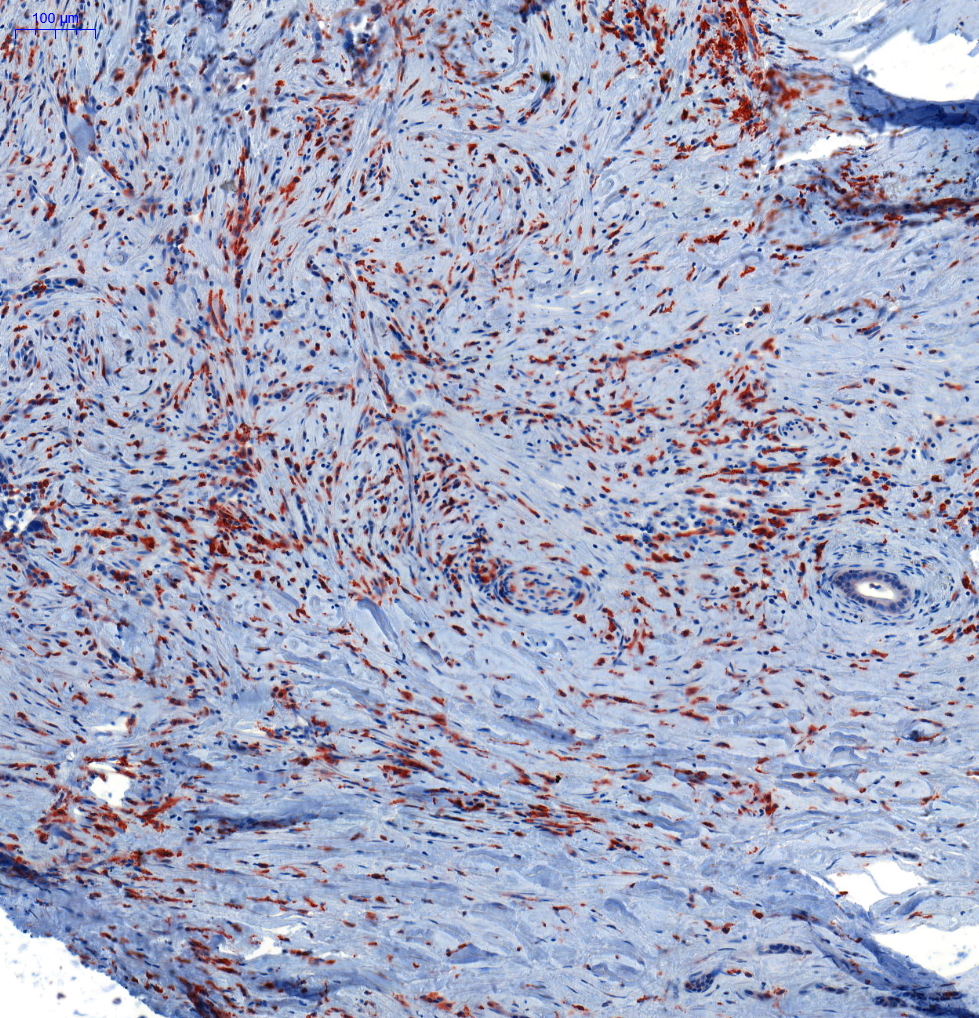

Supplement: Supplementary file 12 — Source Data for Figure 4 [file EMMM-12-e12010-s011.zip › fig4H/FIG4H patientA-CD206_10.0x FAK high.jpg]

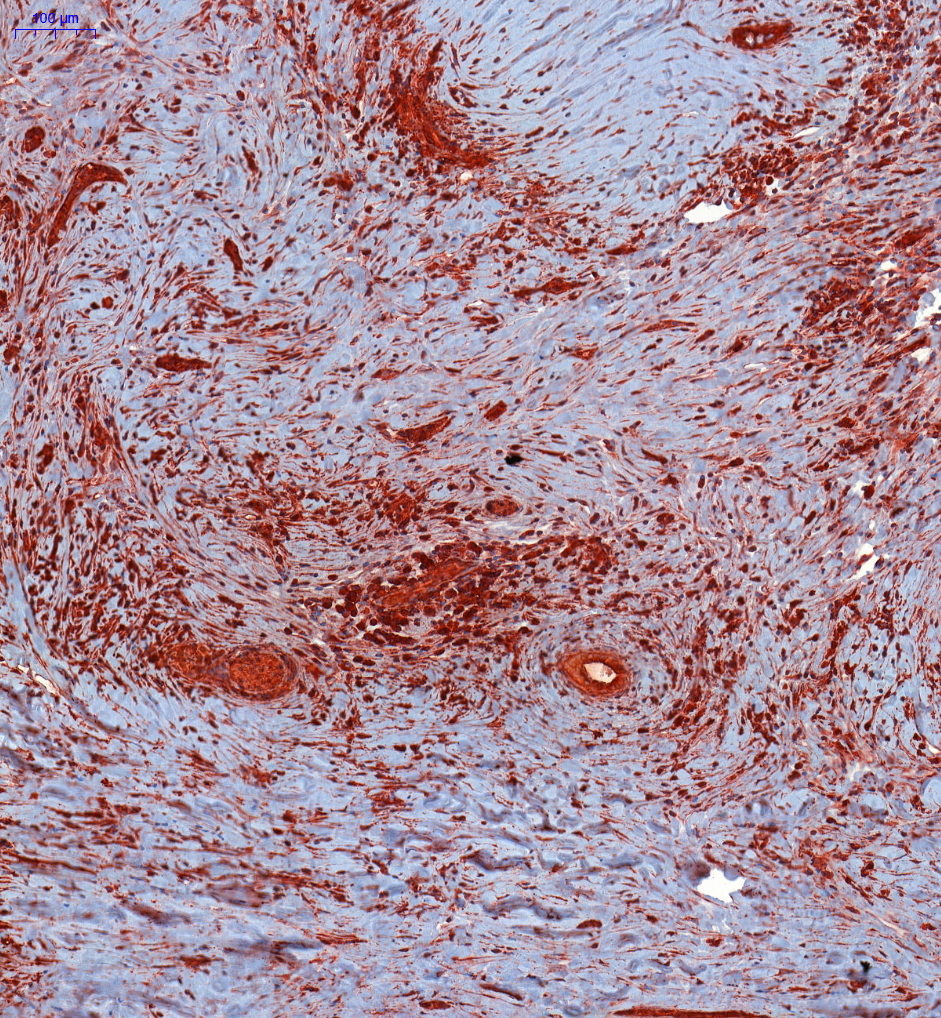

Supplement: Supplementary file 12 — Source Data for Figure 4 [file EMMM-12-e12010-s011.zip › fig4H/FIG4H patientA-pFAK_10.0x FAK high.jpg]

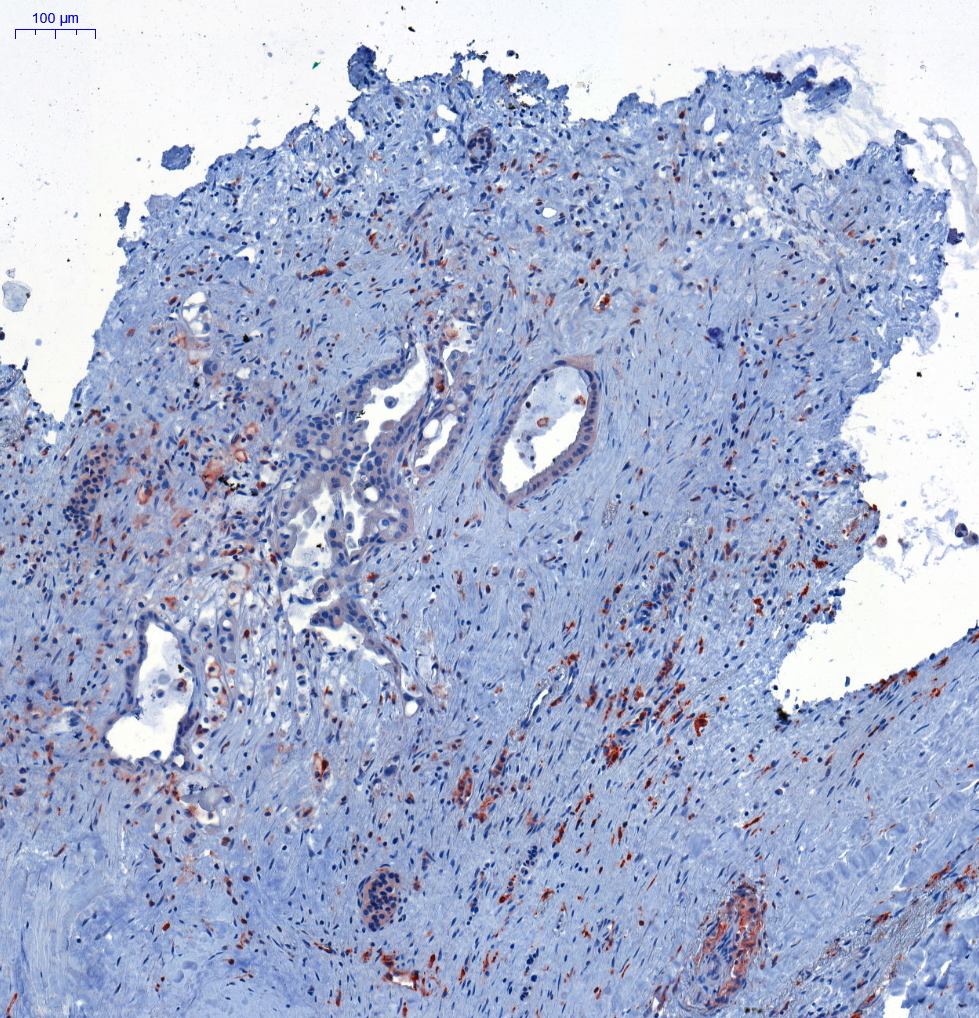

Supplement: Supplementary file 12 — Source Data for Figure 4 [file EMMM-12-e12010-s011.zip › fig4H/FIG4H patientB-CD206_10.0x low FAK.jpg]

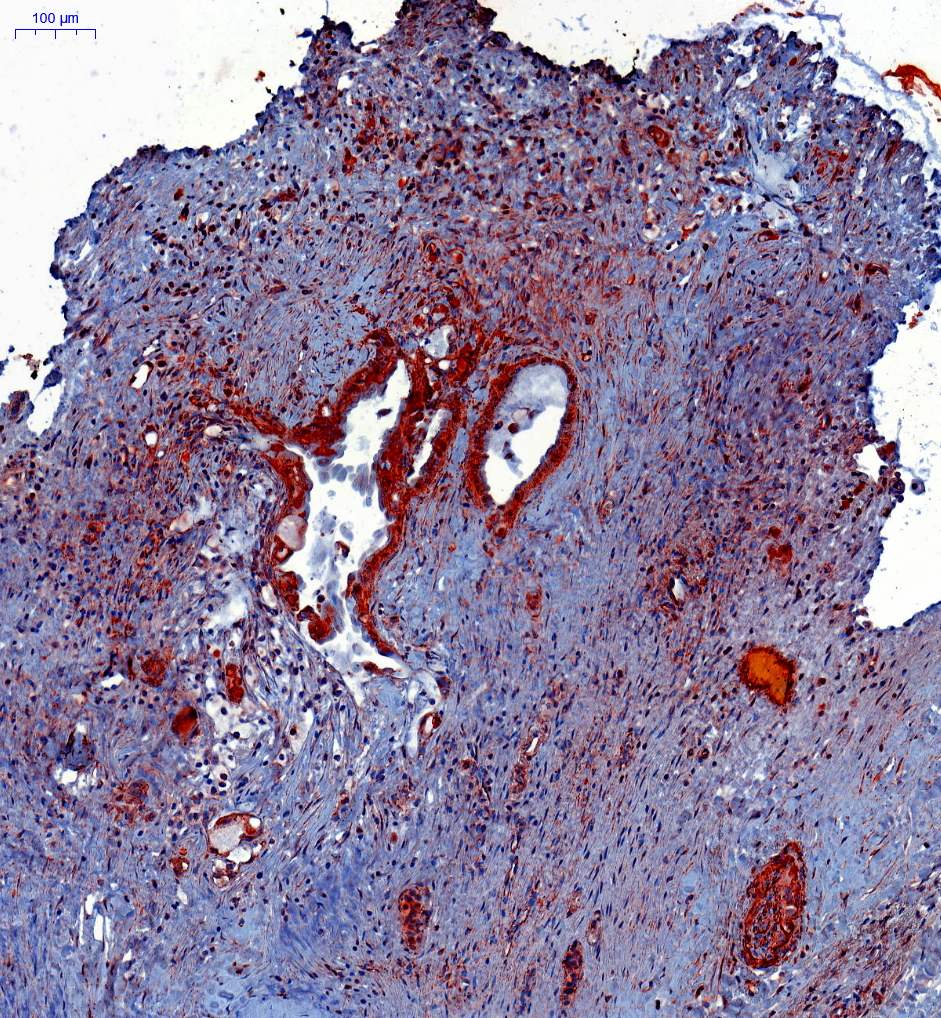

Supplement: Supplementary file 12 — Source Data for Figure 4 [file EMMM-12-e12010-s011.zip › fig4H/FIG4H patientB-pFAK_10.0x low FAK.jpg]

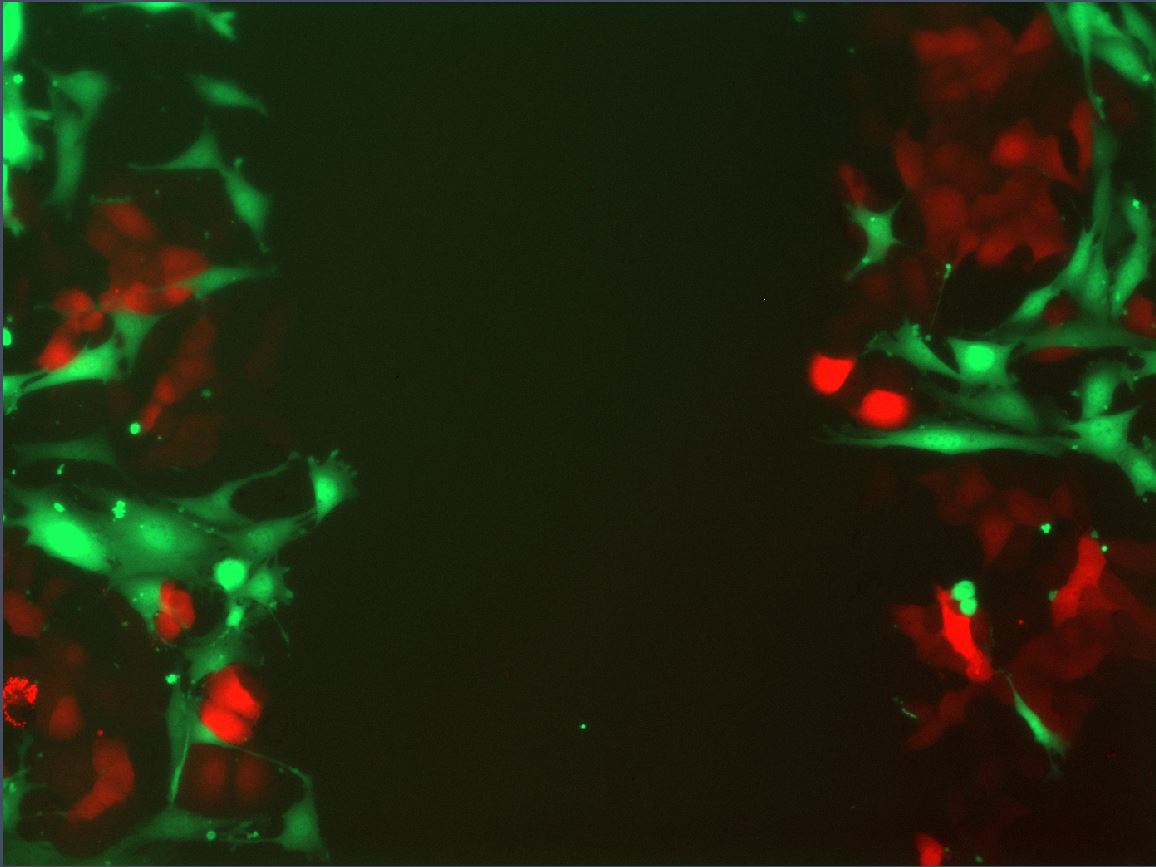

Supplement: Supplementary file 13 — Source Data for Figure 5 [file EMMM-12-e12010-s012.zip › fig5A/FIG5a KDsp R211 0h.JPG]

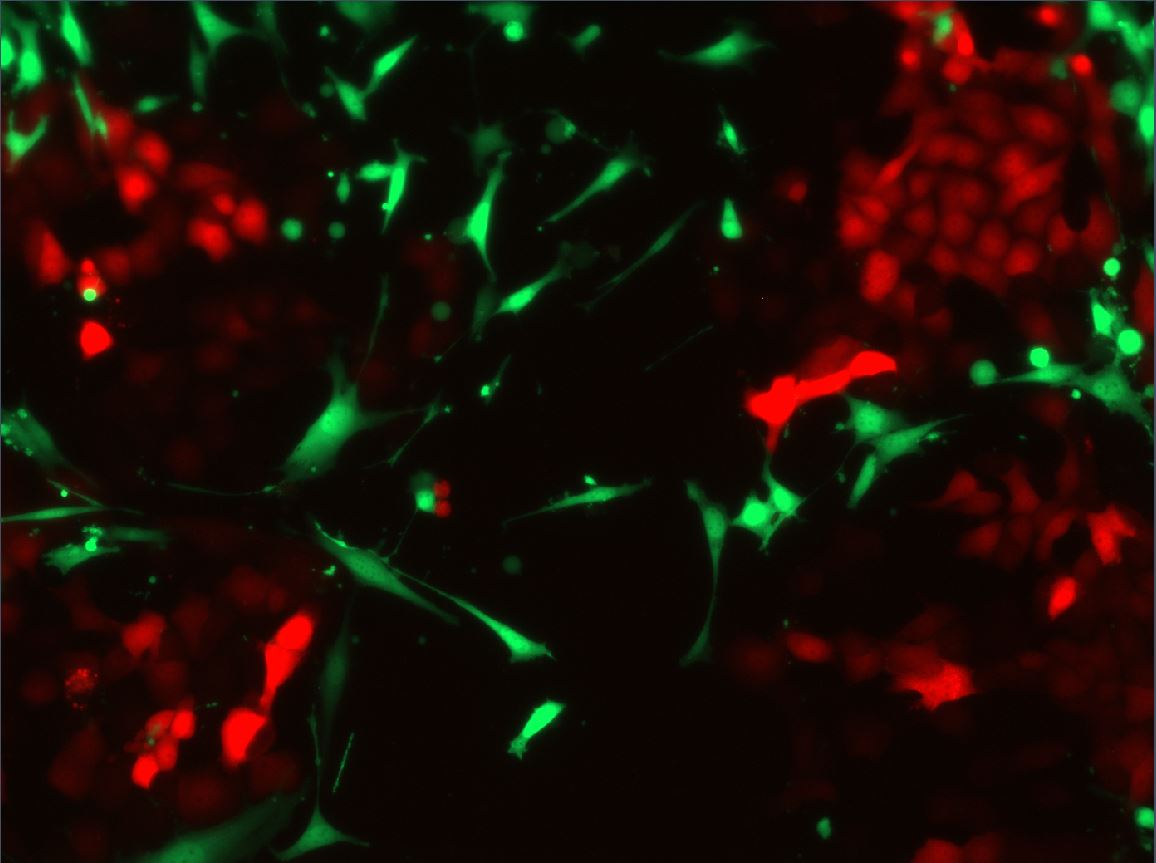

Supplement: Supplementary file 13 — Source Data for Figure 5 [file EMMM-12-e12010-s012.zip › fig5A/FIG5a KDsp R211 48h.JPG]

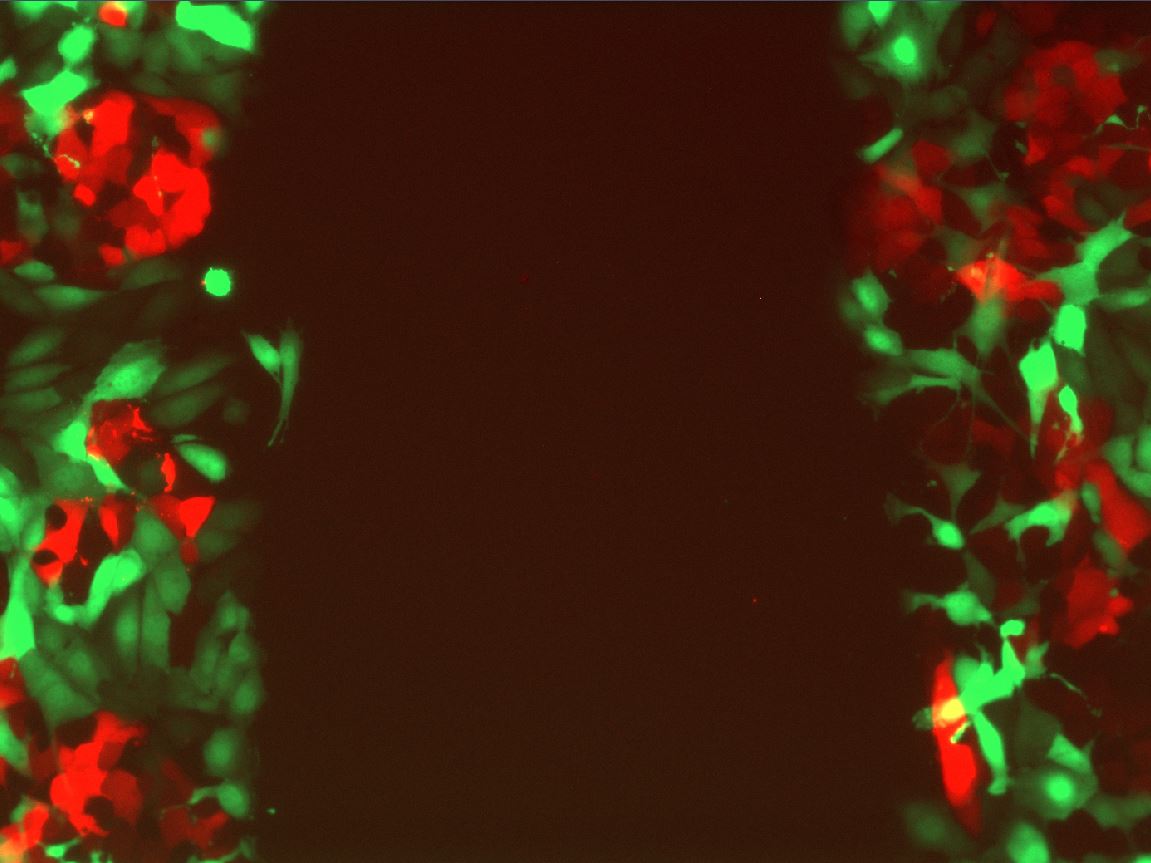

Supplement: Supplementary file 13 — Source Data for Figure 5 [file EMMM-12-e12010-s012.zip › fig5A/FIG5a WTsp R211 0h.JPG]

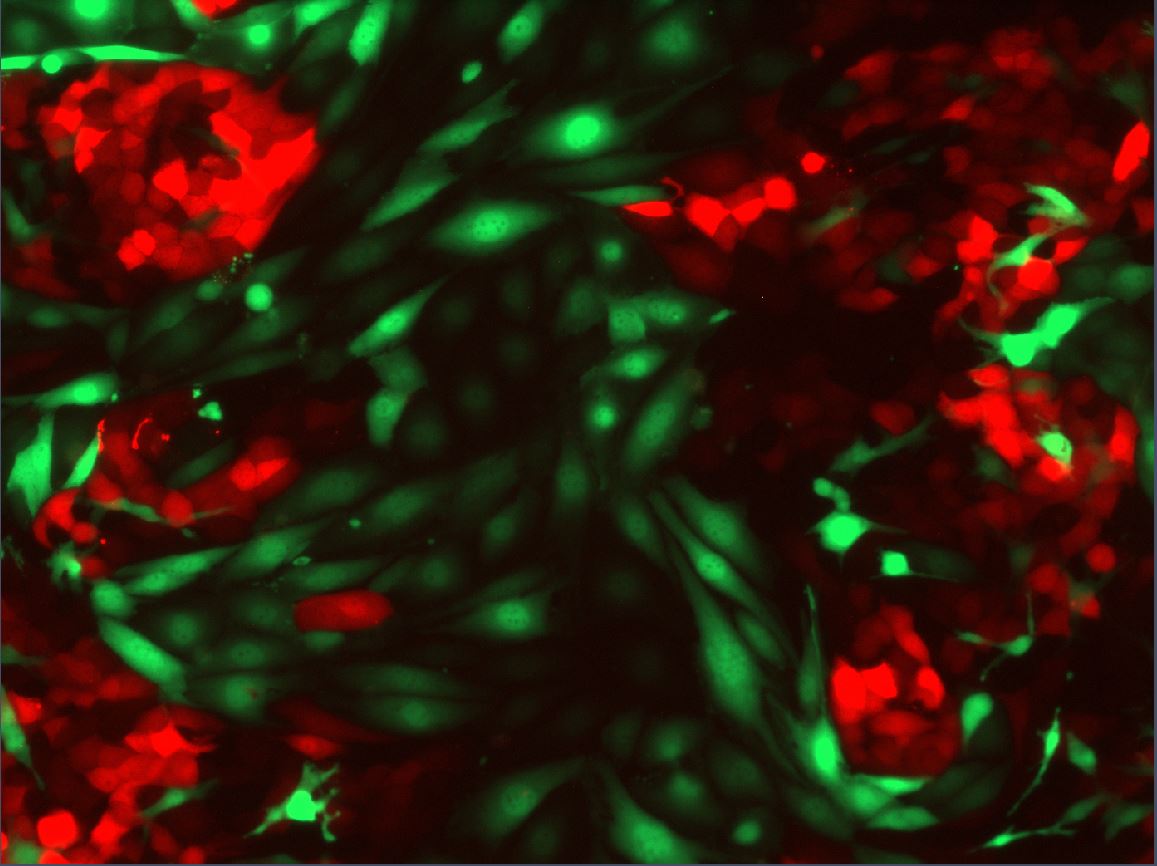

Supplement: Supplementary file 13 — Source Data for Figure 5 [file EMMM-12-e12010-s012.zip › fig5A/FIG5a WTsp R211 48h.JPG]

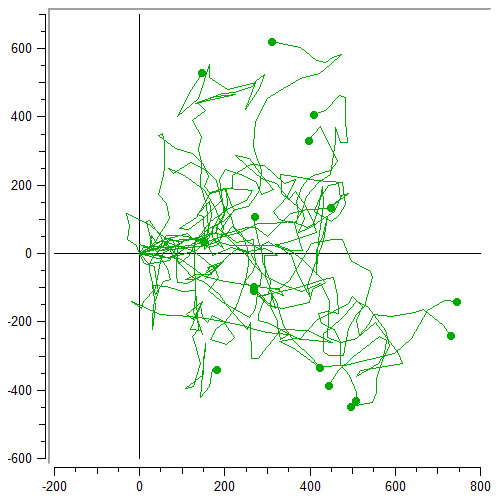

Supplement: Supplementary file 13 — Source Data for Figure 5 [file EMMM-12-e12010-s012.zip › Fig5B/FIG5B migr fakKD fib.jpg]

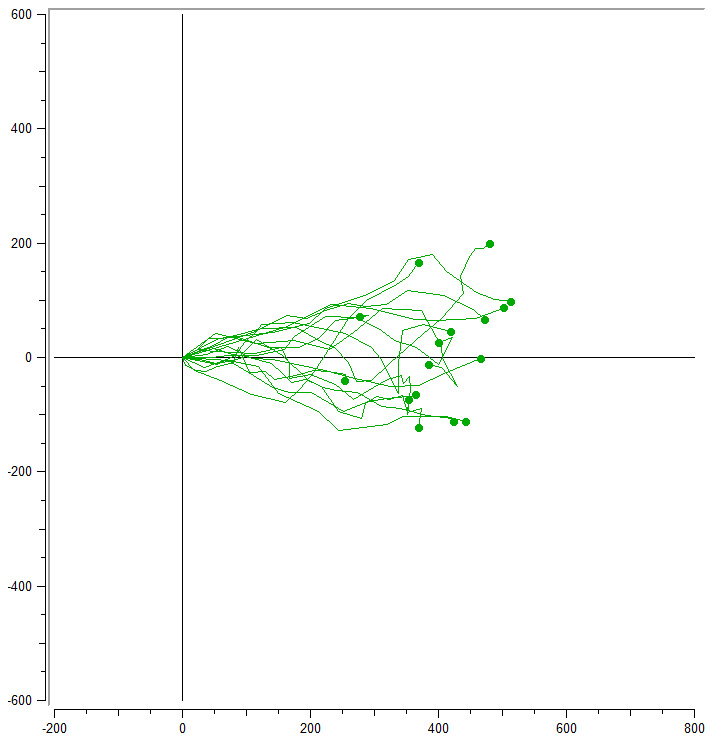

Supplement: Supplementary file 13 — Source Data for Figure 5 [file EMMM-12-e12010-s012.zip › Fig5B/FIG5B migr fakWT fib.jpg]

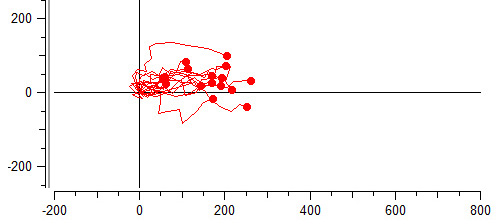

Supplement: Supplementary file 13 — Source Data for Figure 5 [file EMMM-12-e12010-s012.zip › Fig5B/FIG5B migr tum cell with KD fib.jpg]

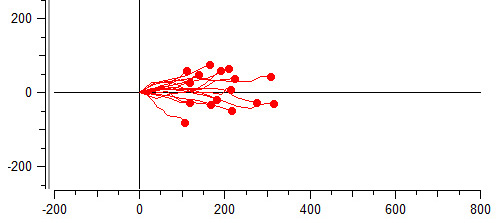

Supplement: Supplementary file 13 — Source Data for Figure 5 [file EMMM-12-e12010-s012.zip › Fig5B/FIG5B migr tum cell with WT fib.jpg]

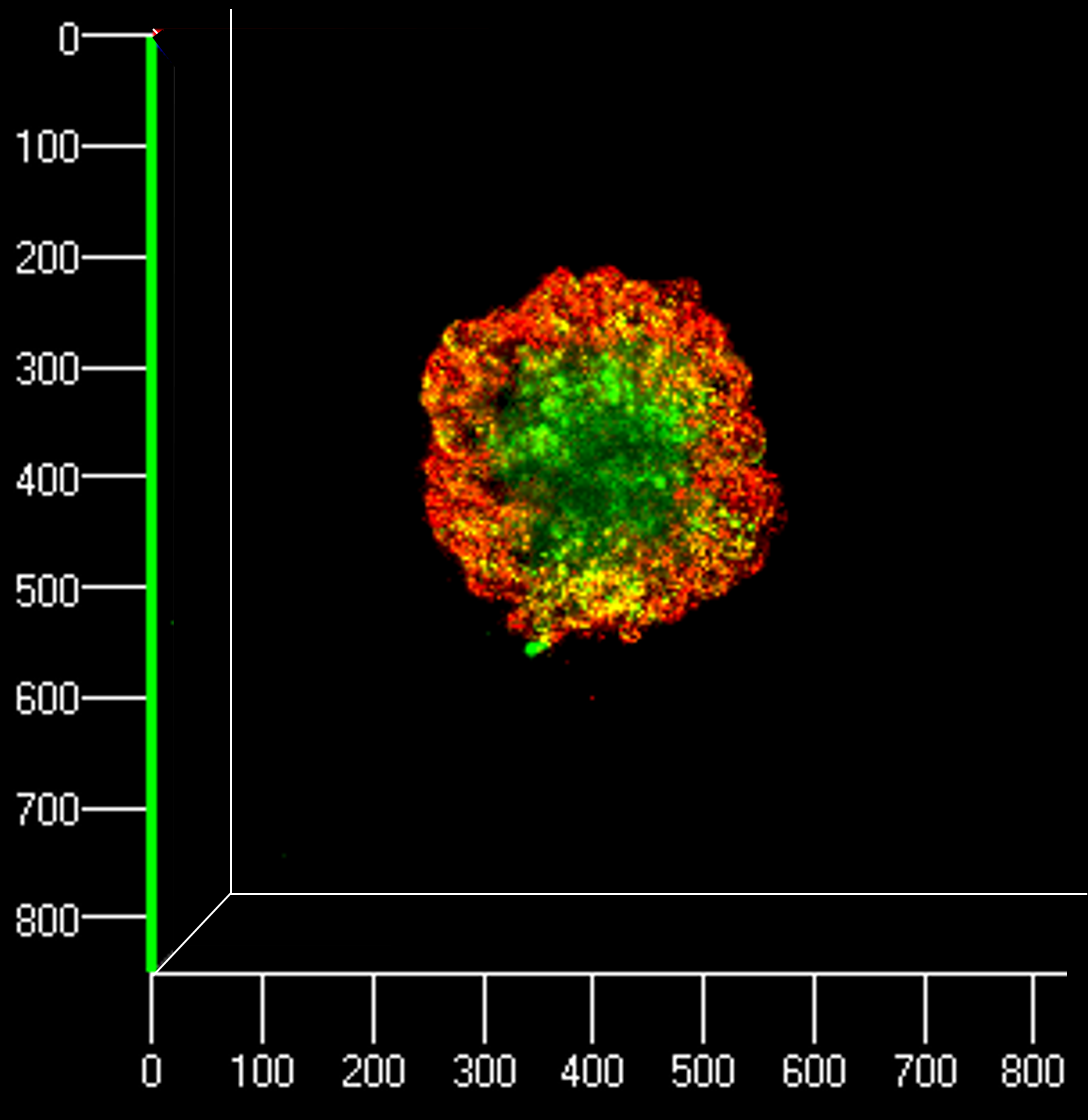

Supplement: Supplementary file 13 — Source Data for Figure 5 [file EMMM-12-e12010-s012.zip › Fig5I/FIG5I inv FAKKD fib 24h.tif]

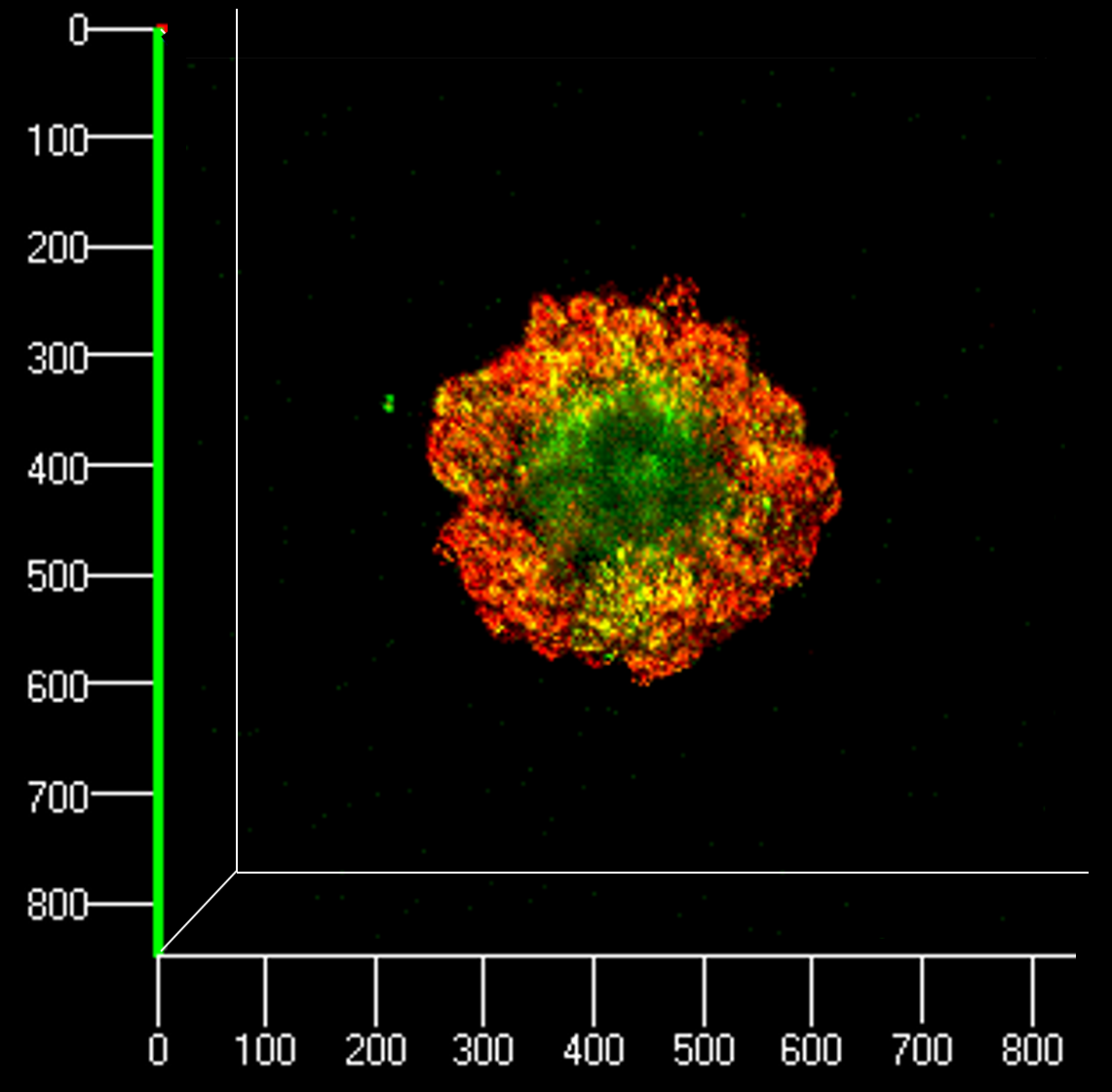

Supplement: Supplementary file 13 — Source Data for Figure 5 [file EMMM-12-e12010-s012.zip › Fig5I/FIG5I inv FAKKD fib 72h.tif]

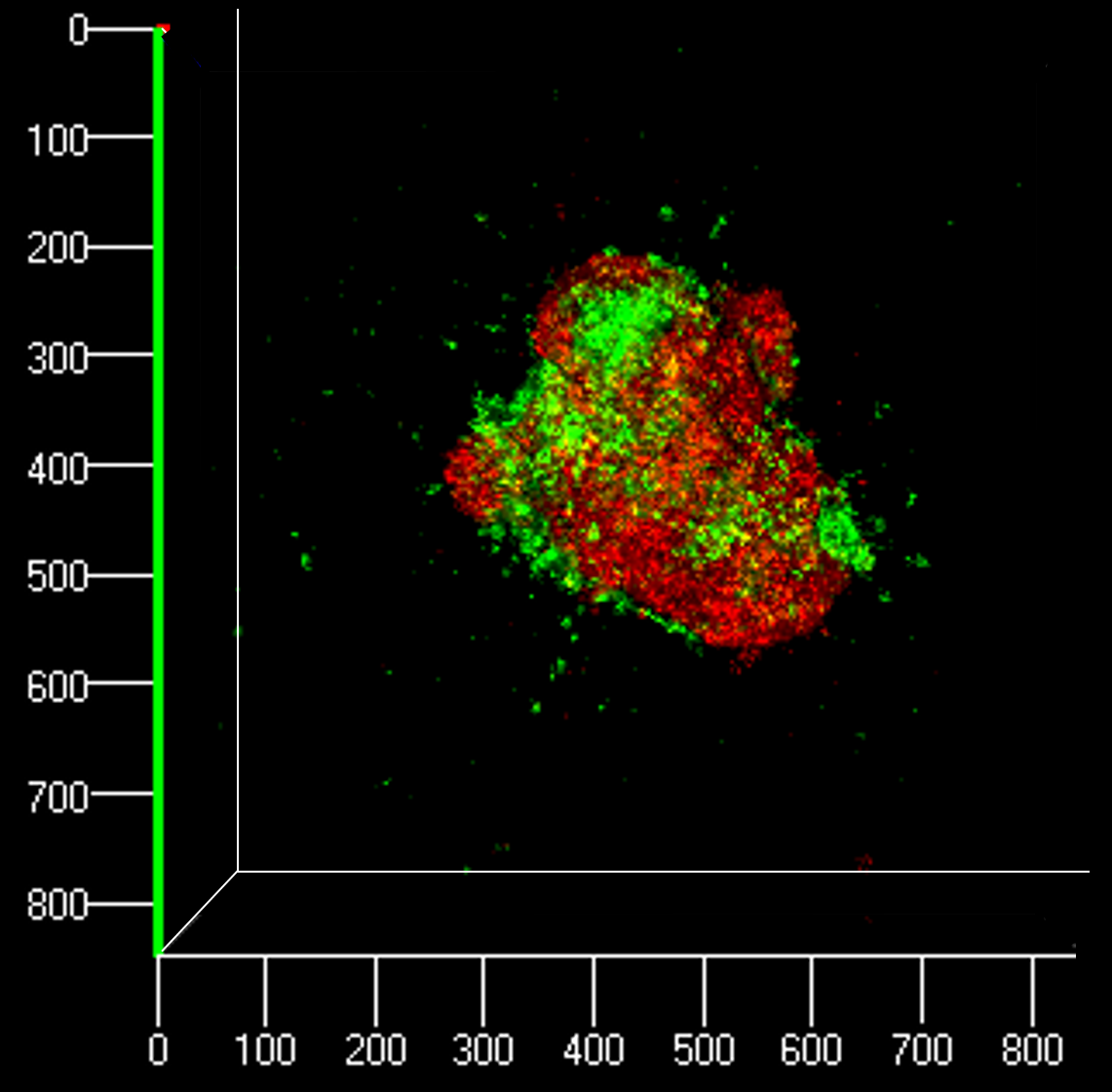

Supplement: Supplementary file 13 — Source Data for Figure 5 [file EMMM-12-e12010-s012.zip › Fig5I/FIG5I inv FAKWT fib 24h.tif]

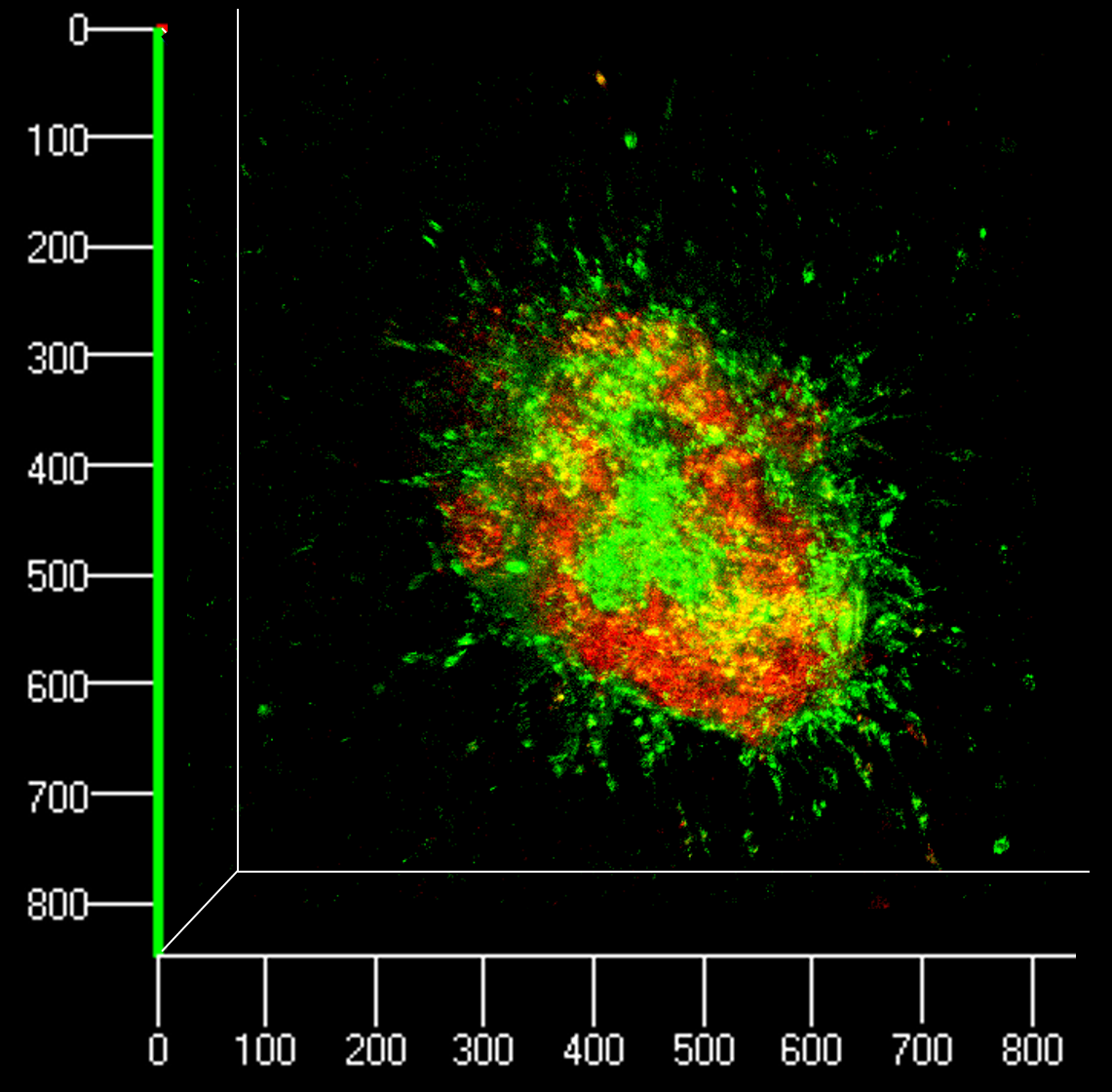

Supplement: Supplementary file 13 — Source Data for Figure 5 [file EMMM-12-e12010-s012.zip › Fig5I/FIG5I inv FAKWT fib 72h.tif]

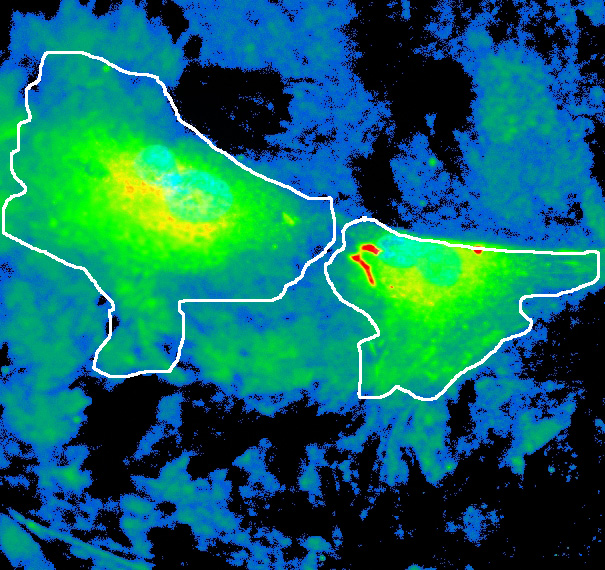

Supplement: Supplementary file 14 — Source Data for Figure 6 [file EMMM-12-e12010-s013.zip › 6E/coll 4 NT heatmap.jpg]

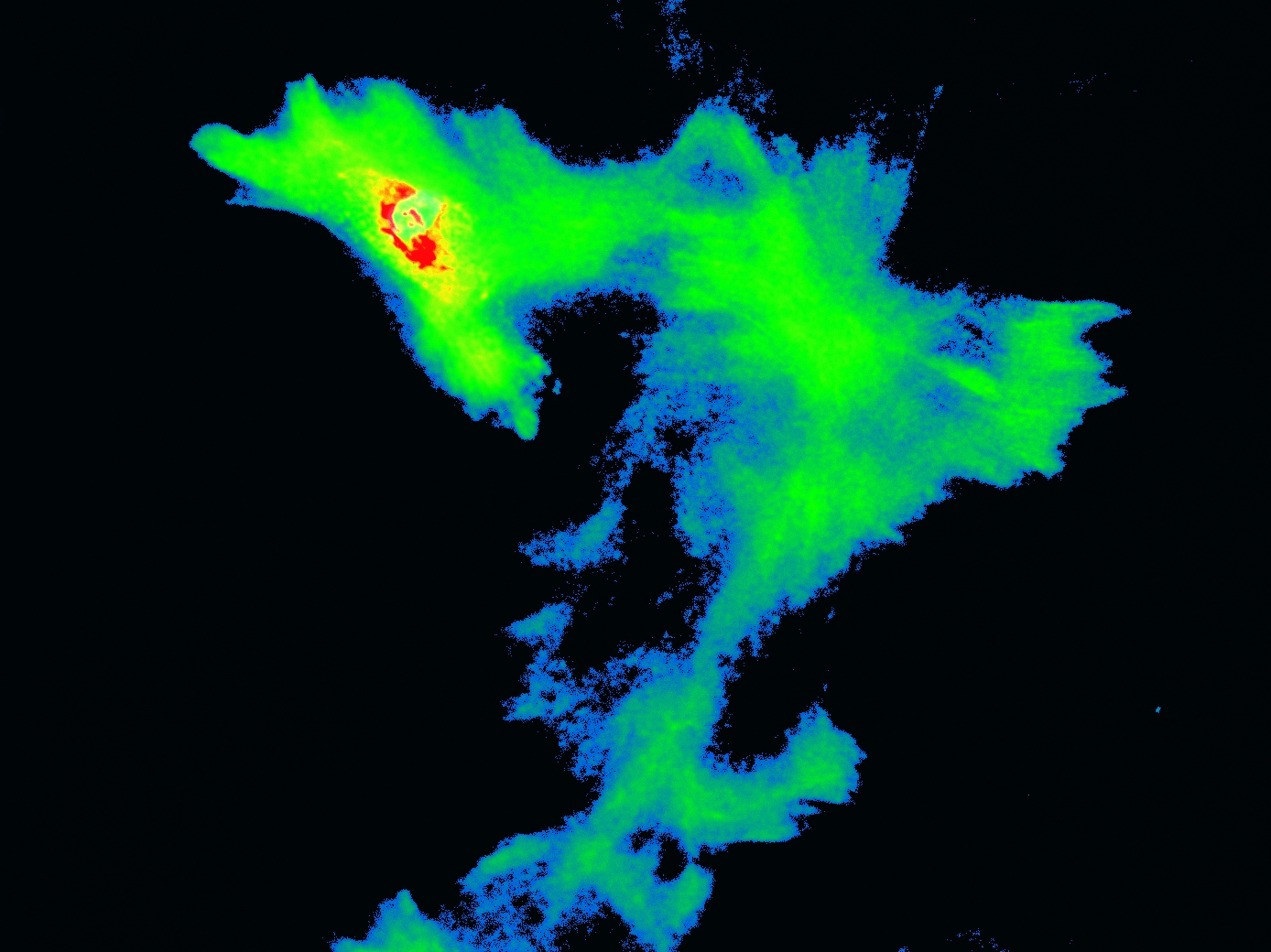

Supplement: Supplementary file 14 — Source Data for Figure 6 [file EMMM-12-e12010-s013.zip › 6E/Fig6E PERIO 647 dapi cafs 20x 4.jpeg]

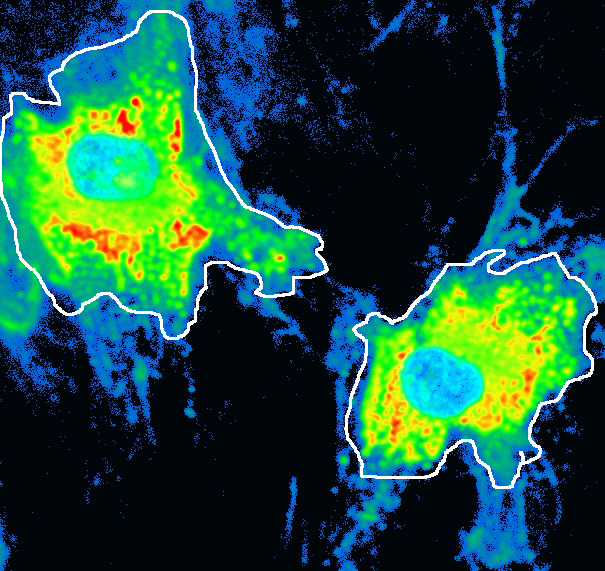

Supplement: Supplementary file 14 — Source Data for Figure 6 [file EMMM-12-e12010-s013.zip › 6E/Fig6E coll 1 NT heatmapr.jpg]

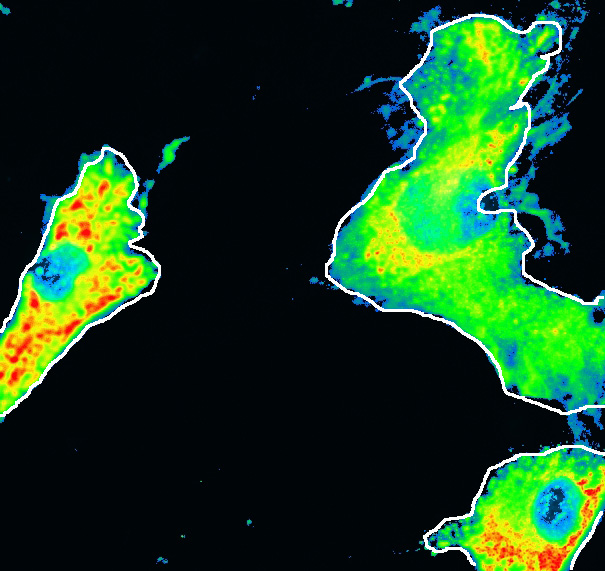

Supplement: Supplementary file 14 — Source Data for Figure 6 [file EMMM-12-e12010-s013.zip › 6E/Fig6E coll 1 FAKi heatmap.jpg]

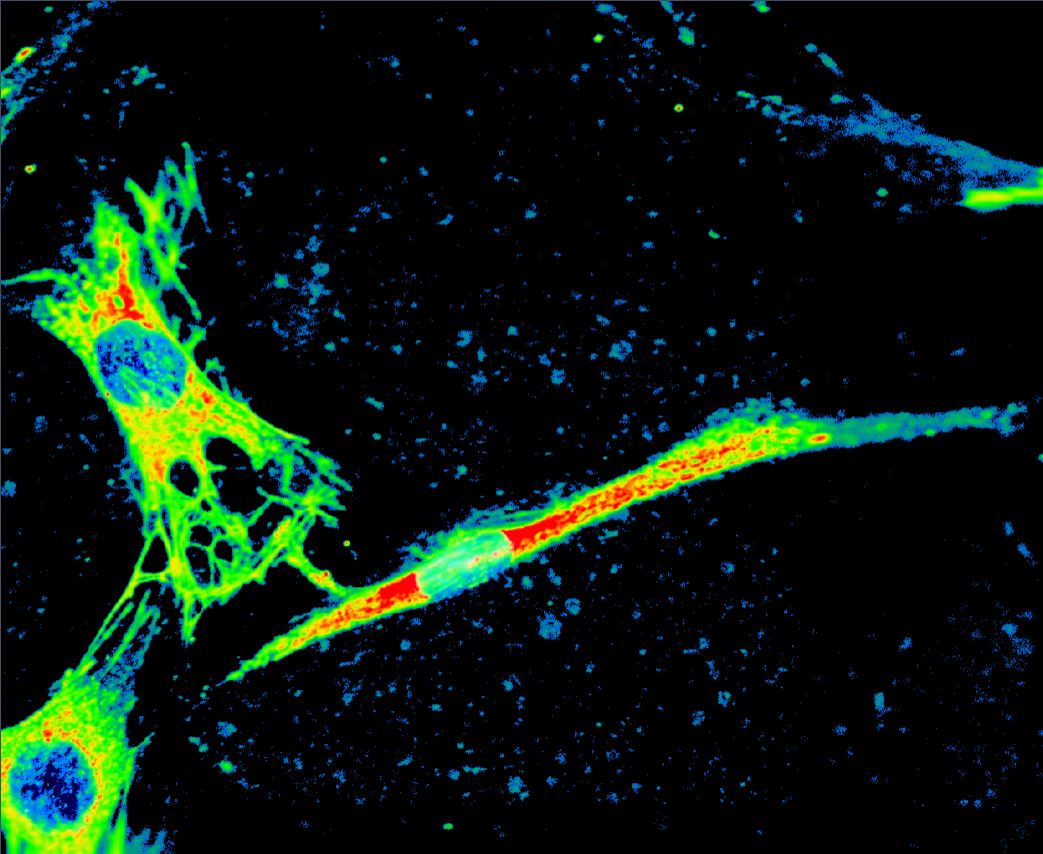

Supplement: Supplementary file 14 — Source Data for Figure 6 [file EMMM-12-e12010-s013.zip › 6E/Fig6E COLL 3 FAKi heatmap.JPG]

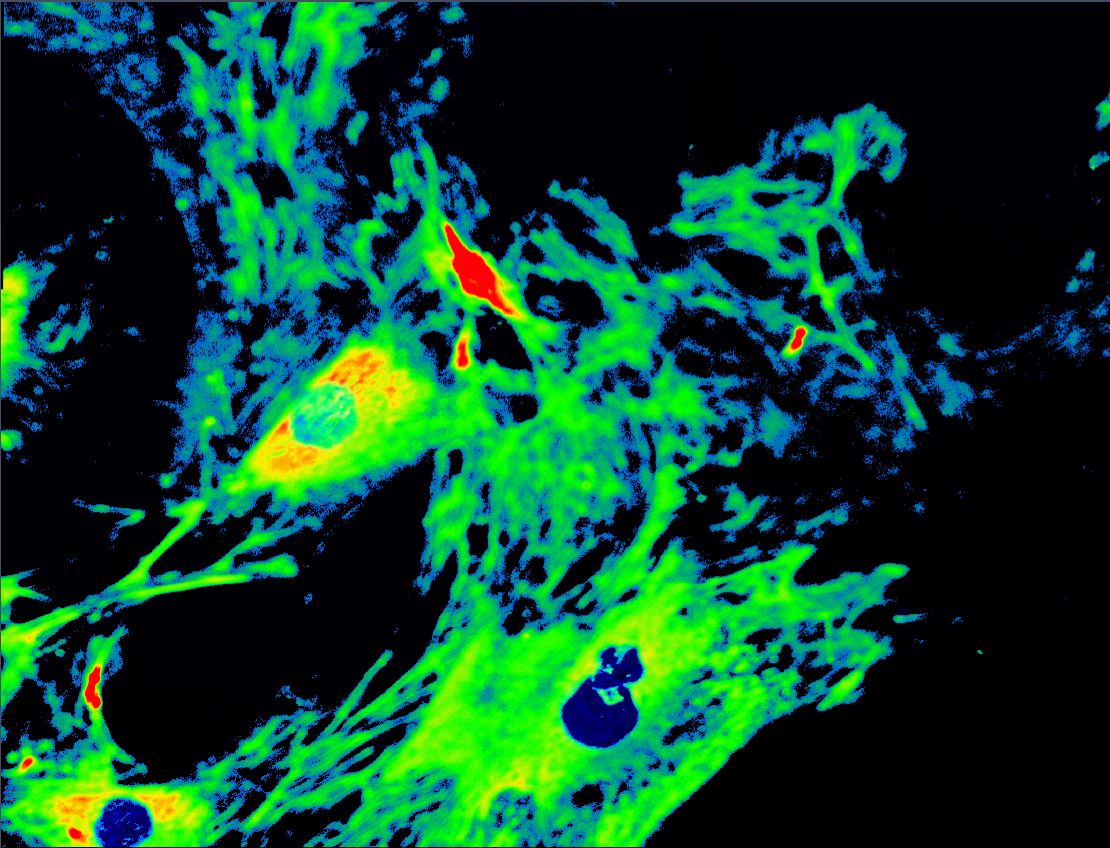

Supplement: Supplementary file 14 — Source Data for Figure 6 [file EMMM-12-e12010-s013.zip › 6E/Fig6E COLL 3 NT heatmap.JPG]

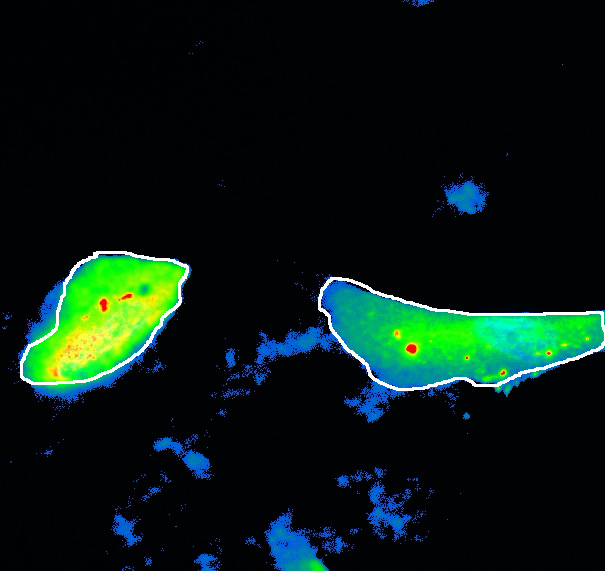

Supplement: Supplementary file 14 — Source Data for Figure 6 [file EMMM-12-e12010-s013.zip › 6E/Fig6E coll 4 FAKi heatmap.jpg]

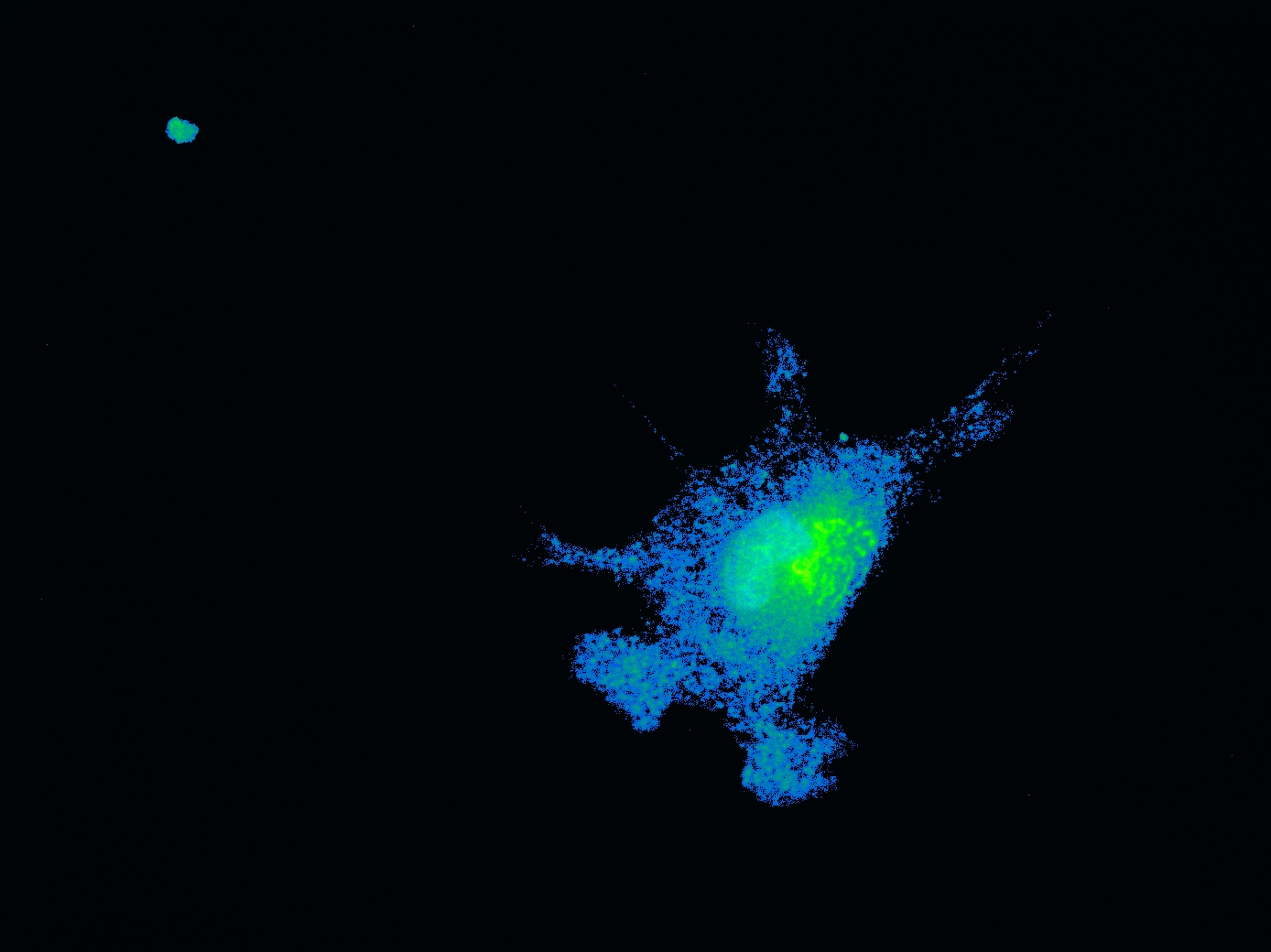

Supplement: Supplementary file 14 — Source Data for Figure 6 [file EMMM-12-e12010-s013.zip › 6E/Fig6E PERIO 647 dapi faki.jpeg]

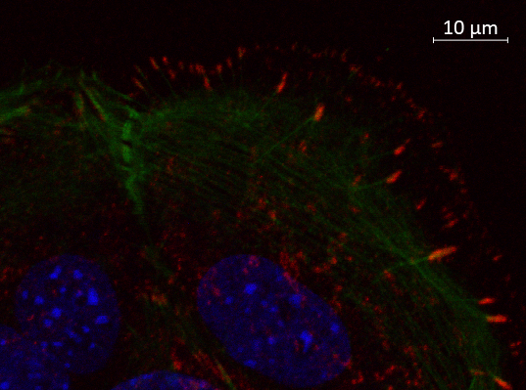

Supplement: Supplementary file 14 — Source Data for Figure 6 [file EMMM-12-e12010-s013.zip › 6L/Fig6EL b activ caf14 faki merge zoom.tif]

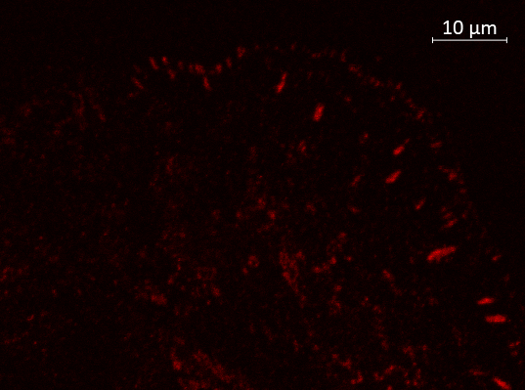

Supplement: Supplementary file 14 — Source Data for Figure 6 [file EMMM-12-e12010-s013.zip › 6L/Fig6EL b activ caf14 faki zoom.tif]

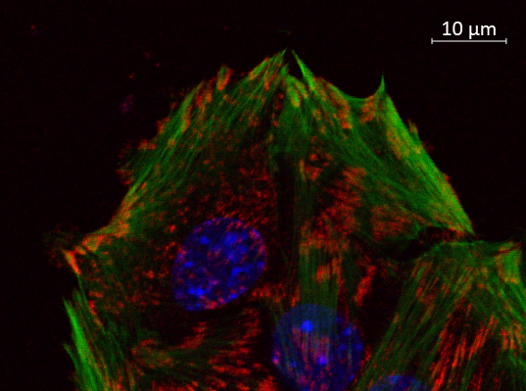

Supplement: Supplementary file 14 — Source Data for Figure 6 [file EMMM-12-e12010-s013.zip › 6L/Fig6EL b activ caf14 merge zoom.tif]

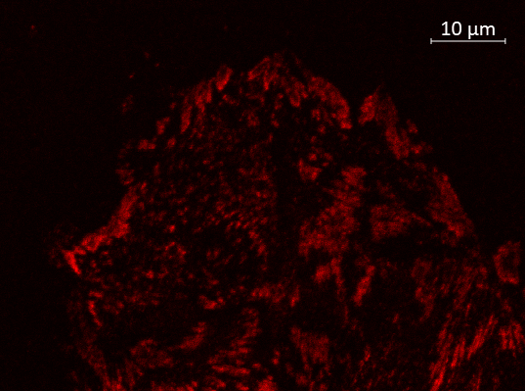

Supplement: Supplementary file 14 — Source Data for Figure 6 [file EMMM-12-e12010-s013.zip › 6L/Fig6EL b activ caf14 zoom.tif]

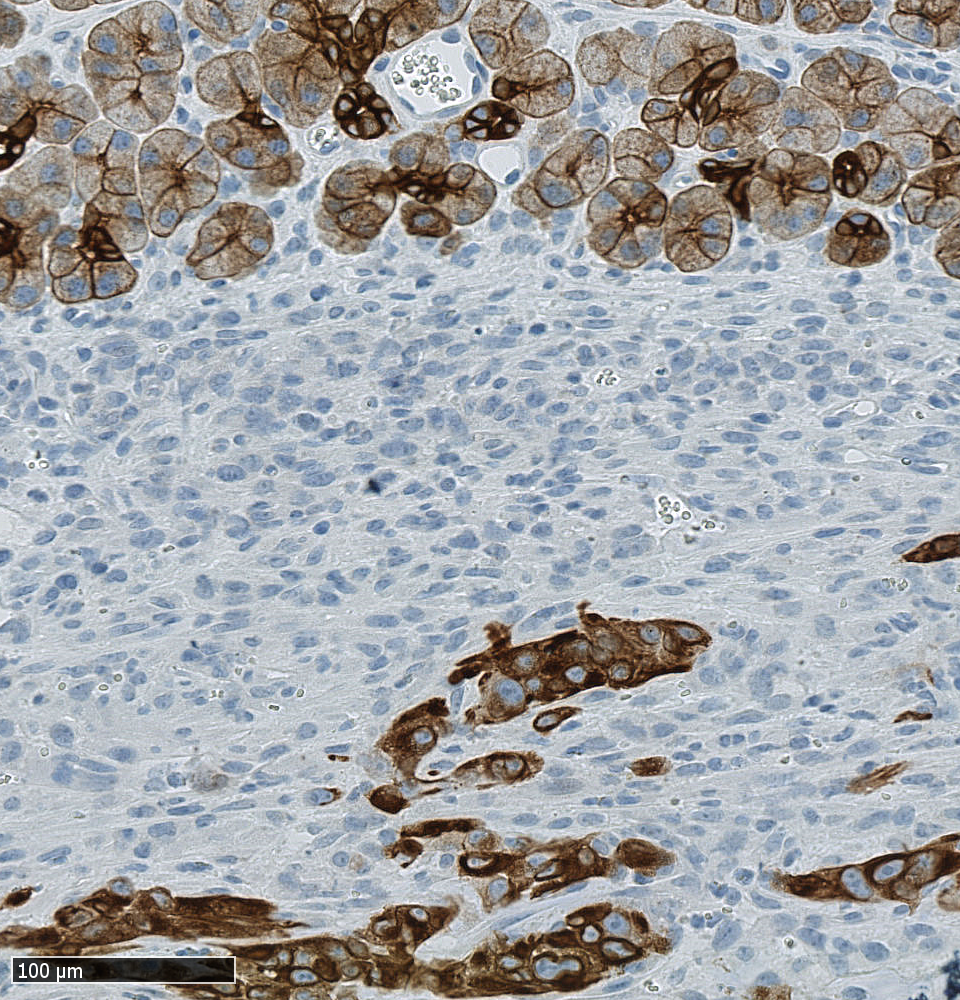

Supplement: Supplementary file 14 — Source Data for Figure 6 [file EMMM-12-e12010-s013.zip › fig6A/Fig6A _KD4PR CK20X.tif]

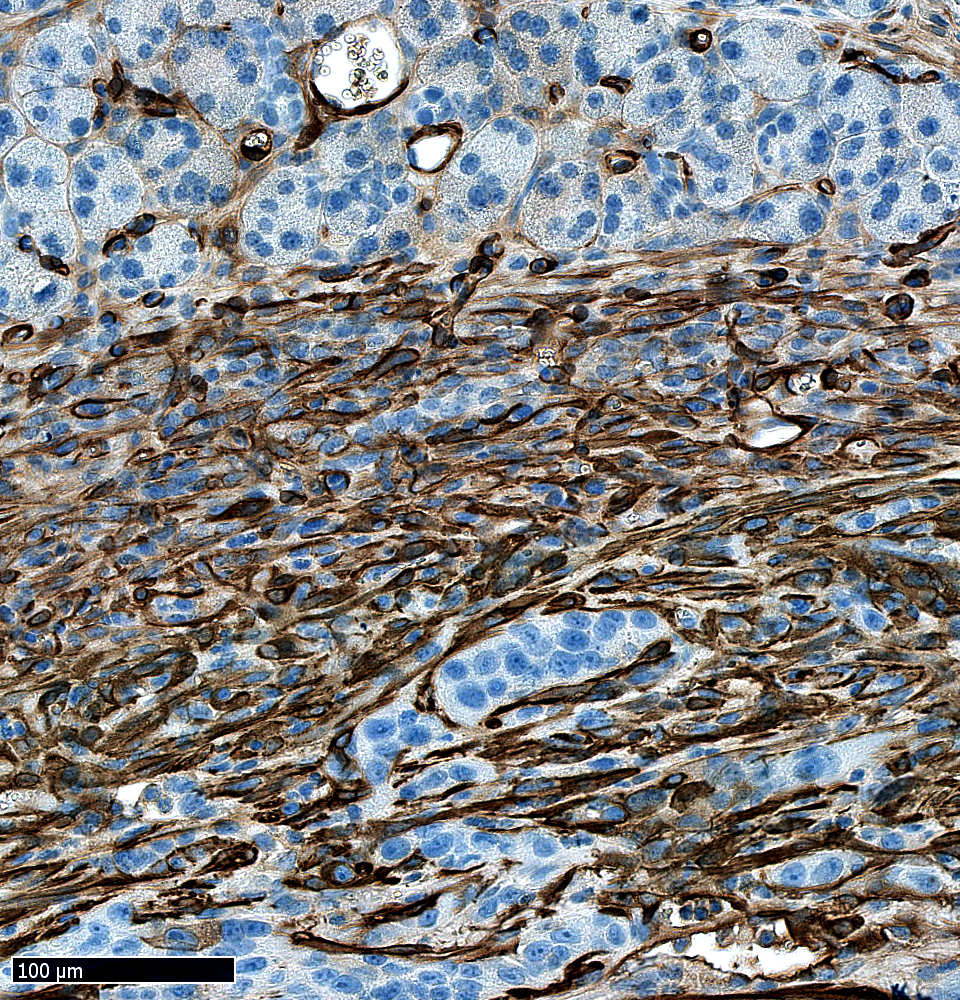

Supplement: Supplementary file 14 — Source Data for Figure 6 [file EMMM-12-e12010-s013.zip › fig6A/Fig6A _KD4PR SMA20X.tif]

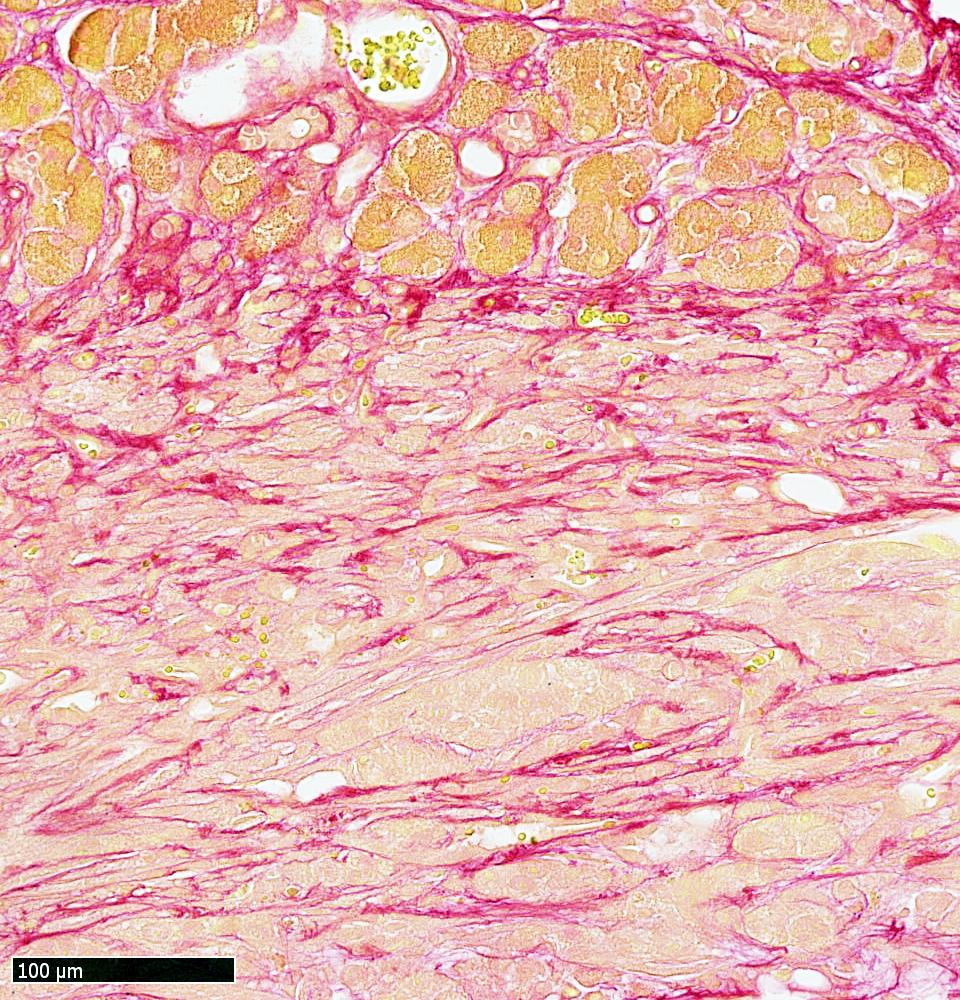

Supplement: Supplementary file 14 — Source Data for Figure 6 [file EMMM-12-e12010-s013.zip › fig6A/Fig6A _KD4PRS20X.tif]

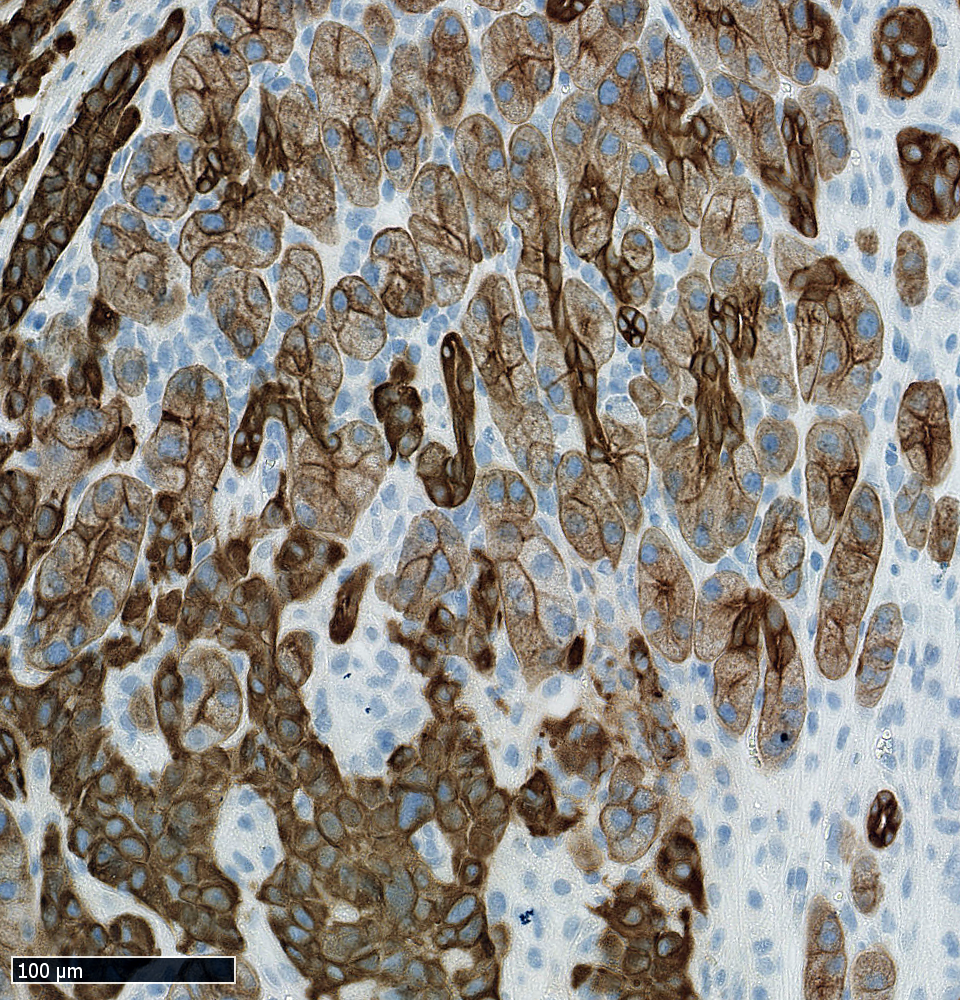

Supplement: Supplementary file 14 — Source Data for Figure 6 [file EMMM-12-e12010-s013.zip › fig6A/Fig6A _WT3PRCK20X.tif]

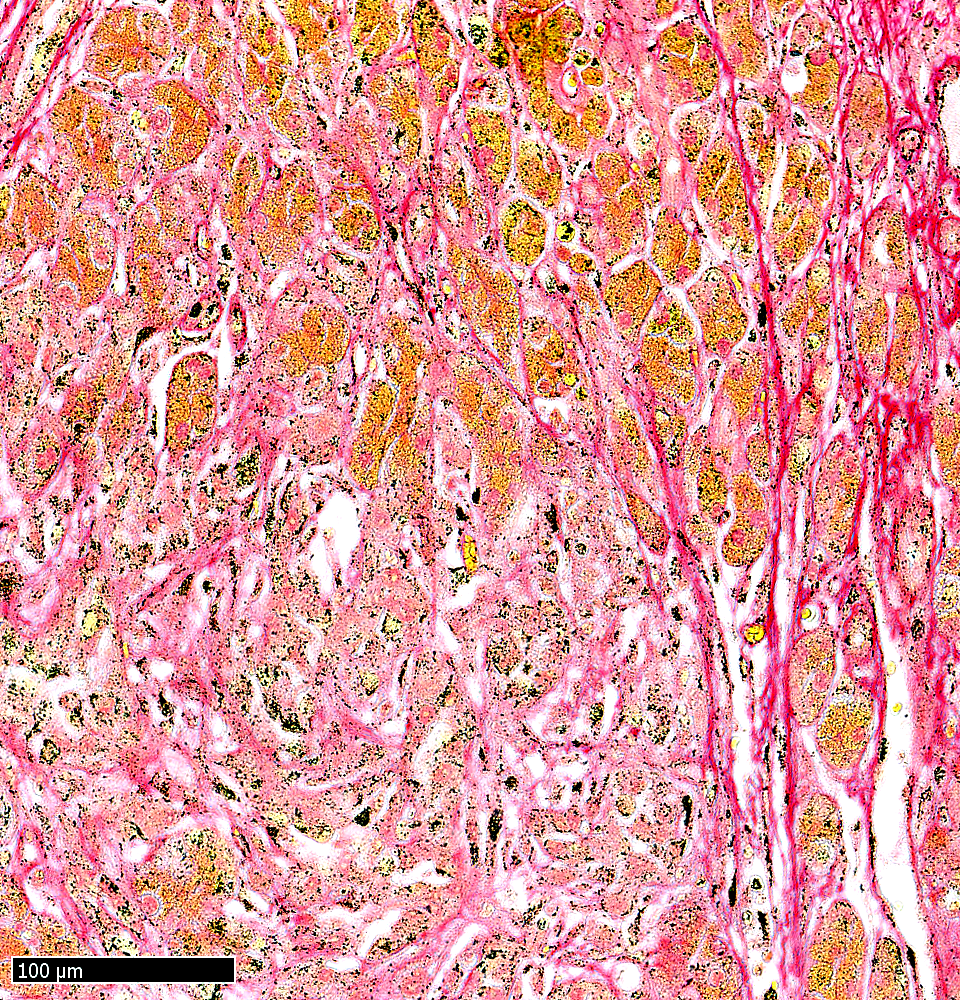

Supplement: Supplementary file 14 — Source Data for Figure 6 [file EMMM-12-e12010-s013.zip › fig6A/Fig6A _WT3PRRS20X.tif]

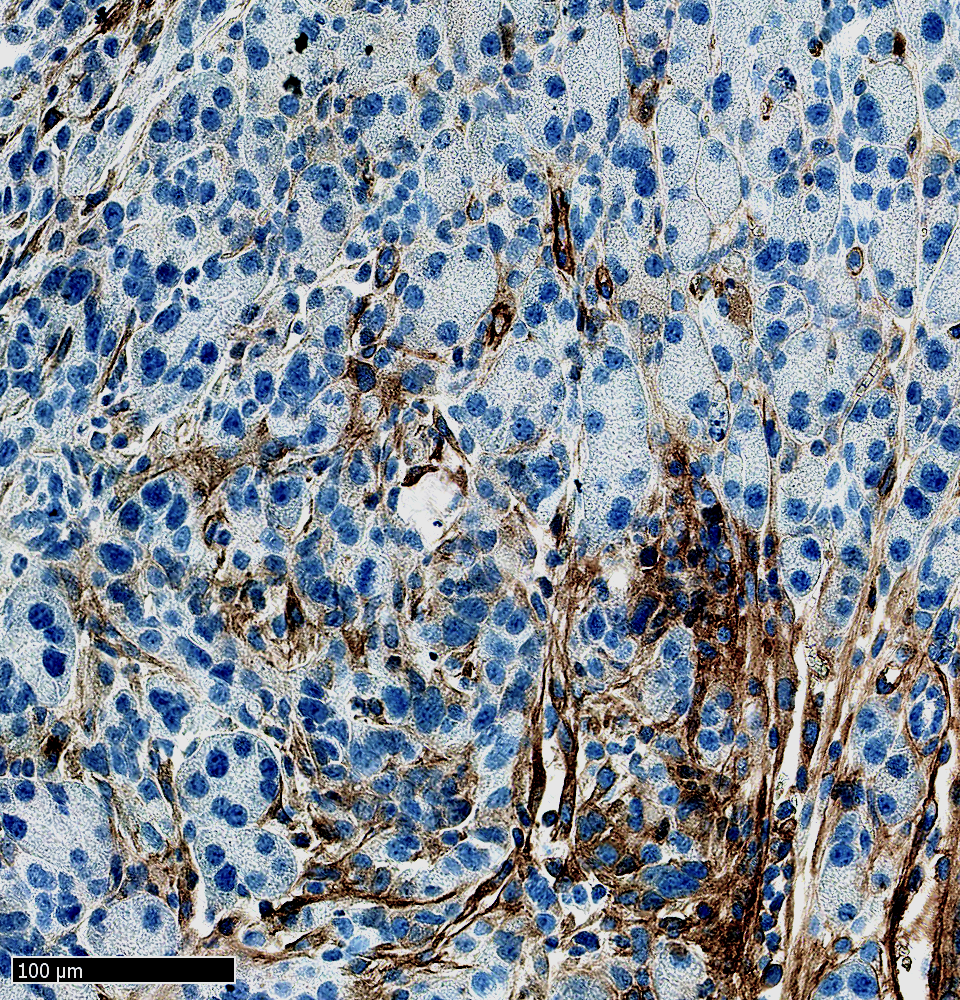

Supplement: Supplementary file 14 — Source Data for Figure 6 [file EMMM-12-e12010-s013.zip › fig6A/Fig6A _WT3PRSMA20X.tif]

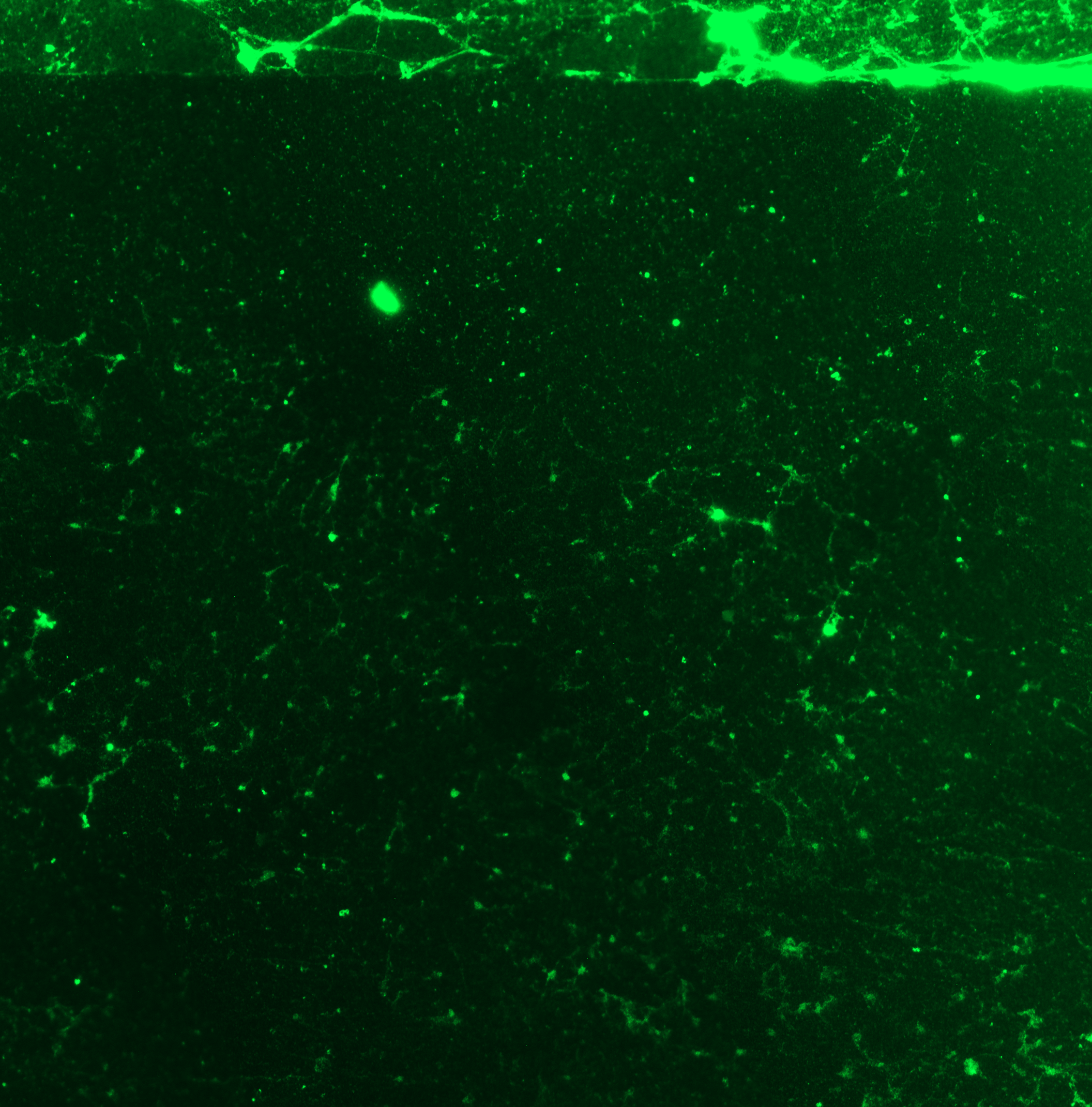

Supplement: Supplementary file 14 — Source Data for Figure 6 [file EMMM-12-e12010-s013.zip › fig6B/Fig6B KD ECM scratch crop3.tif]

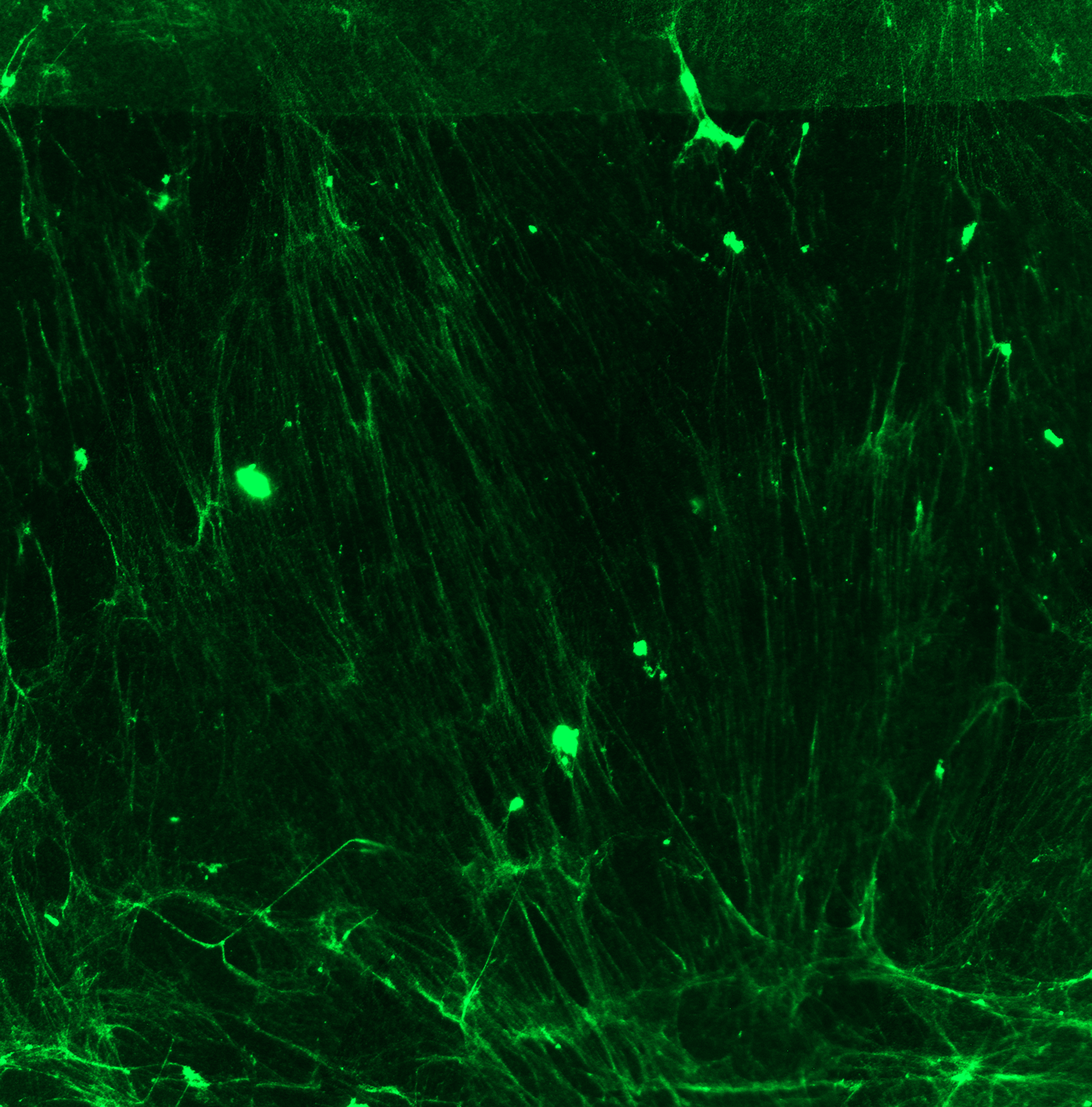

Supplement: Supplementary file 14 — Source Data for Figure 6 [file EMMM-12-e12010-s013.zip › fig6B/Fig6B WT ECM scratch crop3.tif]
